# Phosphorylation of the VAR2CSA extracellular region is associated with enhanced adhesive properties to the placental receptor CSA

**DOI:** 10.1371/journal.pbio.3000308

**Published:** 2019-06-10

**Authors:** Dominique Dorin-Semblat, Marilou Tétard, Aurélie Claës, Jean-Philippe Semblat, Sébastien Dechavanne, Zaineb Fourati, Romain Hamelin, Florence Armand, Graziella Matesic, Sofia Nunes-Silva, Anand Srivastava, Stéphane Gangnard, Jose-Juan Lopez-Rubio, Marc Moniatte, Christian Doerig, Artur Scherf, Benoît Gamain

**Affiliations:** 1 Université de Paris, Biologie Intégrée du Globule Rouge, UMR_S1134, BIGR, INSERM, Paris, France; 2 Institut National de la Transfusion Sanguine, Paris, France; 3 Unité de Biologie des Interactions Hôte-Parasite, Institut Pasteur, Paris, France; 4 INSERM U1201, Paris, France; 5 CNRS ERL9195, Paris, France; 6 Proteomics Core Facility, Ecole Polytechnique Fédérale de Lausanne, Lausanne, Switzerland; 7 CNRS5290, IRD224, University Montpellier, MIVEGEC, Montpellier, France; 8 Centre for Chronic Infectious and Inflammation Disease, Biomedical Sciences Cluster, School of Health and Biomedical Sciences, RMIT University, Bundoora, Victoria, Australia; University of Florida, UNITED STATES

## Abstract

*Plasmodium falciparum* is the main cause of disease and death from malaria. *P*. *falciparum* virulence resides in the ability of infected erythrocytes (IEs) to sequester in various tissues through the interaction between members of the polymorphic *P*. *falciparum* erythrocyte membrane protein 1 (PfEMP1) adhesin family to various host receptors. Here, we investigated the effect of phosphorylation of variant surface antigen 2-CSA (VAR2CSA), a member of the PfEMP1 family associated to placental sequestration, on its capacity to adhere to chondroitin sulfate A (CSA) present on the placental syncytium. We showed that phosphatase treatment of IEs impairs cytoadhesion to CSA. MS analysis of recombinant VAR2CSA phosphosites prior to and after phosphatase treatment, as well as of native VAR2CSA expressed on IEs, identified critical phosphoresidues associated with CSA binding. Site-directed mutagenesis on recombinant VAR2CSA of 3 phosphoresidues localised within the CSA-binding region confirmed in vitro their functional importance. Furthermore, using clustered regularly interspaced short palindromic repeats/CRISPR-associated protein-9 nuclease (CRISPR/Cas9), we generated a parasite line in which the phosphoresidue T934 is changed to alanine and showed that this mutation strongly impairs IEs cytoadhesion to CSA. Taken together, these results demonstrate that phosphorylation of the extracellular region of VAR2CSA plays a major role in IEs cytoadhesion to CSA and provide new molecular insights for strategies aiming to reduce the morbidity and mortality of PM.

## Abstract 2

Placental sequestration of malaria-infected erythrocytes is mediated by the interaction between parasite VAR2CSA and the placental receptor chondroitin sulfate A; this study shows that phosphorylation of VAR2CSA plays a major role in this process, with implications for protecting pregnant women and their babies against placental malaria.

## Introduction

The most severe forms of malaria are caused by the protozoan parasite *Plasmodium falciparum* [1], whose virulence is associated with an immune evasion strategy based on antigenic variation of parasite-encoded adhesins responsible for infected erythrocyte (IEs)- sequestration from blood circulation by binding to endothelial cells’ surface receptors within microvessels of various tissues [2,3]. Adhesion is mediated by members of the highly polymorphic *P*. *falciparum* erythrocyte membrane protein 1 (PfEMP1) encoded by the *var* gene family [4–6]. As a consequence of epigenetics-based allelic exclusion mechanisms, a single *var* gene is expressed at any given time, and the corresponding PfEMP1 is exported to the IEs surface [7,8]. Whereas the intracellular acidic terminal segment (ATS) of PfEMP1, also termed VARC, is highly conserved, the extracellular region displays an N-terminal segment (NTS) followed by various numbers of highly polymorphic Duffy-binding–like (DBL) and cysteine-rich interdomain region (CIDR) domains [9,10]. PfEMP1 proteins localise to electron-dense protrusions of the IEs’s membrane, known as ‘knobs’. The parasite knob-associated histidine-rich protein (KAHRP) is crucial in knob formation and in anchoring PfEMP1 through the binding to its cytoplasmic domain VARC [11].

Placental malaria (PM) is characterised by the massive accumulation of IEs and monocytes in the placental intervillous blood spaces and causes adverse birth outcomes, notably low birth weight and increased perinatal and maternal mortality [12,13]. Chondroitin sulfate A (CSA) is the primary receptor for IEs sequestration in the placenta [14,15]. Variant surface antigen 2-CSA (VAR2CSA) is the PfEMP1 family member responsible for IEs cytoadhesion to CSA. Indeed, *var2csa* is the only *var* gene expressed in CSA-binding–selected IEs [16–18], and *Δvar2csa* mutant clones lose the CSA-binding phenotype [19,20]. Furthermore, the full-length VAR2CSA extracellular region adheres with high specificity and affinity to CSA due to the presence of a CSA-binding site in the interdomain-1–interdomain-2a (ID1–ID2a) region [21–23]. However, mechanisms regulating the interaction of placental IEs to CSA have not been addressed so far. Phosphorylation of cell surface proteins is an important mechanism in the regulation of many physiological processes, including extracellular signaling, cell polarity, cell–cell interactions, and cell adhesion [24–26]. Phosphorylation of various extracellular domains by the Casein Kinase 2 (CK2) has been shown to modulate cell adhesion in various human cell lines [24,27]. Phosphorylation of the PfEMP1 cytoplasmic domain VARC by human CK2 increases its affinity for KAHRP and may play a role in IEs cytoadhesion [28]. However, neither the phosphorylation of PfEMP1 extracellular region nor its possible effect on PfEMP1 adhesive properties have been addressed.

Here, we report that the extracellular region of VAR2CSA is phosphorylated. Mass spectrometry (MS) analysis of recombinant and of native VAR2CSA expressed on IEs identified critical phosphoresidues associated with CSA binding. A significant reduction of full-length recombinant VAR2CSA adhesion to CSA was observed after mutation to alanine of several phosphoresidues located within the ID1-ID2a CSA-binding site. However, no decrease of CSA adhesion was observed when the conserved ID1-ID2a phosphorylated T934 residue was mutated to aspartic acid to mimic the phosphoresidue. Phosphatase treatment also strongly inhibited IEs adhesion to CSA and Human placenta choriocarcinoma (BeWo) cells in both static and flow conditions. Finally, using clustered regularly interspaced short palindromic repeats/ caspase 9 (CRISPR/Cas9), we generated a parasite line in which the T934 is substituted by alanine and showed that the mutation strongly impairs IEs cytoadhesion to CSA under both static and flow conditions.

## Results

### Phosphatase treatment inhibits cytoadhesion of IEs expressing VAR2CSA

**Fig 1:**
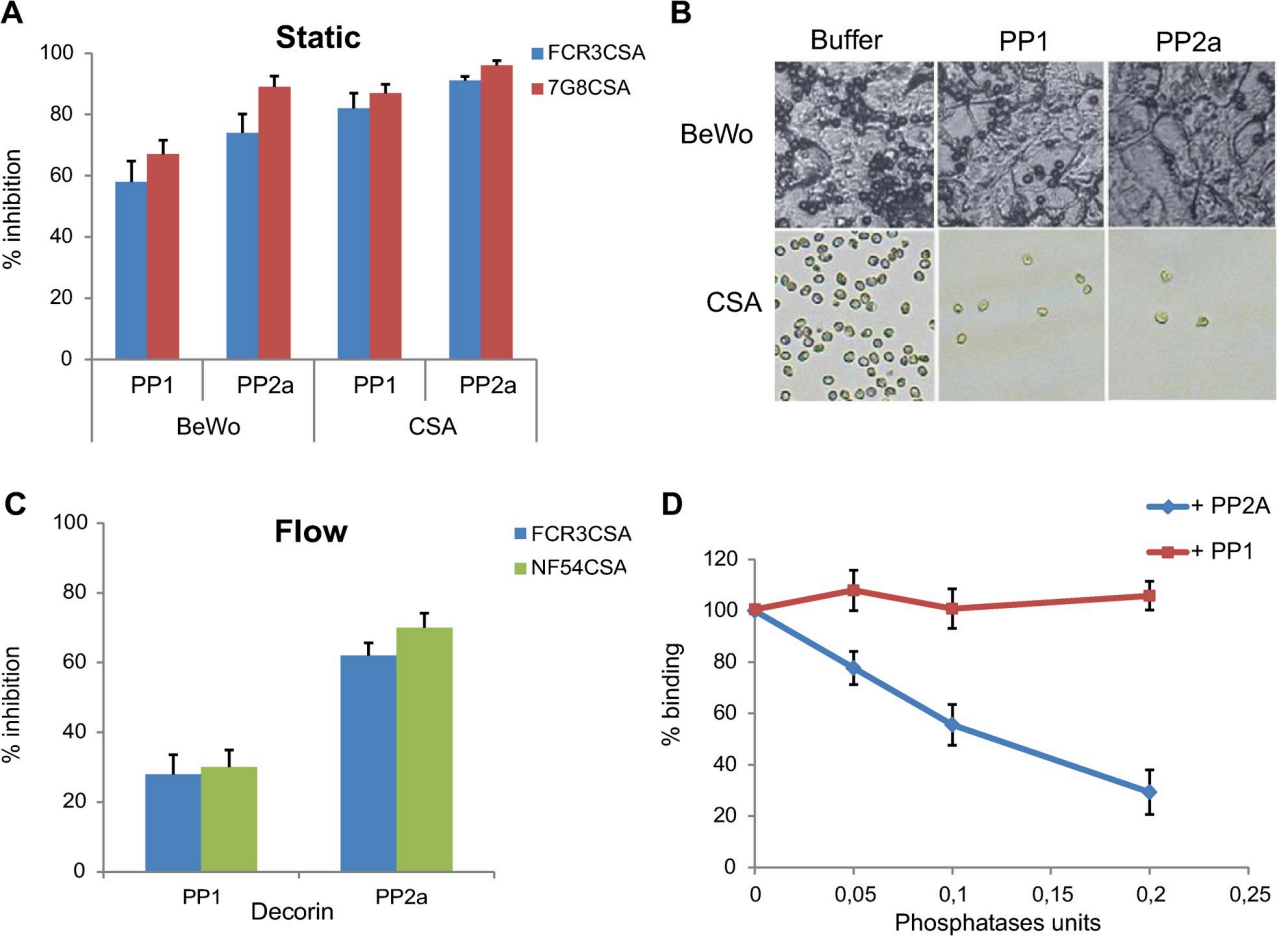
Phosphatases treatment decreases rVAR2CSA and IE cytoadhesion under static and flow conditions. (A) 7G8CSA or FCR3CSA IEs at trophozoite mid and late stages pretreated or not for 30 min with 0.1 units of PP1or PP2a were assayed for binding on a monolayer layer of BeWo cells or on CSA-coated plastic spots. Bound IEs were counted in 5 random fields, and results were expressed as percentage of inhibition compared to buffer-treated IEs (100% binding/0% inhibition). (B) Typical images of a field showing IEs bound to BeWo cells or to CSA-coated plastic. (C) NF54CSA or FCR3CSA IEs pretreated or not with both phosphatases were assayed for adhesion on decorin-coated Vena8 Endothelial+ biochips. A shear stress of 0.05 Pa was applied for 5 min. Numbers of adherent IEs were counted in 5 positions in the channel, and results were expressed as percentage inhibition compared with untreated IEs (100% binding). (D) Binding of recombinant VAR2CSA pretreated or not with increasing units of PP1 or PP2a was monitored using an ELISA-based direct binding assay with coated decorin. Error bars correspond to SD between 4 independent experiments. Each experiment was performed in duplicate. Numerical values that underline the graphs are shown in S1 Data. BeWo, human placenta choriocarcinoma; CSA, chondroitin sulfate A; ELISA, enzyme-linked immunosorbent assay; IEs, infected erythrocytes; PP1, protein phosphatase 1; PP2a, protein phosphatase 2a; rVAR2CSA, recombinant VAR2CSA. NF54, *Plasmodium falciparum* strain NF54; NF54CSA, *Plasmodium falciparum* strain NF54 selected for CSA adhesion; FCR3, *Plasmodium falciparum* strain FCR3; FCR3CSA, *Plasmodium falciparum* strain FCR3 selected for CSA adhesion; 7G8, *Plasmodium falciparum* strain 7G8; 7G8CSA, *Plasmodium falciparum* strain 7G8 selected for CSA adhesion.

To assess a possible functional role of phosphorylation in IEs cytoadhesion, we measured the effect of serine-threonine protein phosphatase 1 (PP1) and serine-threonine protein phosphatase 2a (PP2a) treatment on IEs adhesion. Using static binding assays on immobilised cells from the BeWo placental line, we showed that IEs adhesion of the CSA-selected parasite lines 7G8CSA and FCR3CSA was significantly reduced after treatment with either phosphatase, compared with the untreated IEs (Fig 1A). Phosphatase treatment also strongly inhibited IEs adhesion to CSA immobilised on plastic (Fig 1A). Representative photographs of bound IEs to BeWo cells or CSA are shown in Fig 1B. Flow-based cytoadhesion assays at a shear stress of 0.05 Pa, mimicking wall shear stress in the placenta [29], performed with NF54CSA and FCR3CSA, revealed that phosphatase pretreatment also inhibits IEs adherence to chips coated with decorin, a proteoglycan carrying CSA (Fig 1C). PP1 treatment has a less drastic effect on cytoadhesion than PP2a treatment.

### Phosphatase treatment inhibits rVAR2CSA adhesion in vitro

Because the central role of VAR2CSA is to mediate IEs cytoadhesion to CSA present on the syncytiotrophoblast layer, we next investigated whether PP1 or PP2a treatment of the 3D7 VAR2CSA full-length extracellular recombinant protein (rVAR2CSA) expressed in human embryonic kidney (HEK)293 cells could affect its binding to decorin. We observed a significant dose-dependent reduction of rVAR2CSA adhesion with increasing concentrations of PP2a, but no effect of PP1 pretreatment (Fig 1D). Incubation of PP2a with okadaic acid, a potent PP2a inhibitor [30] had no effect on rVAR2CSA adhesion to decorin (S1 Fig). No degradation of rVAR2CSA protein was observed after phosphatases treatments (S2 Fig).

### rVAR2CSA produced in HEK293 and endogenous VAR2CSA are phosphorylated

**Fig 2:**
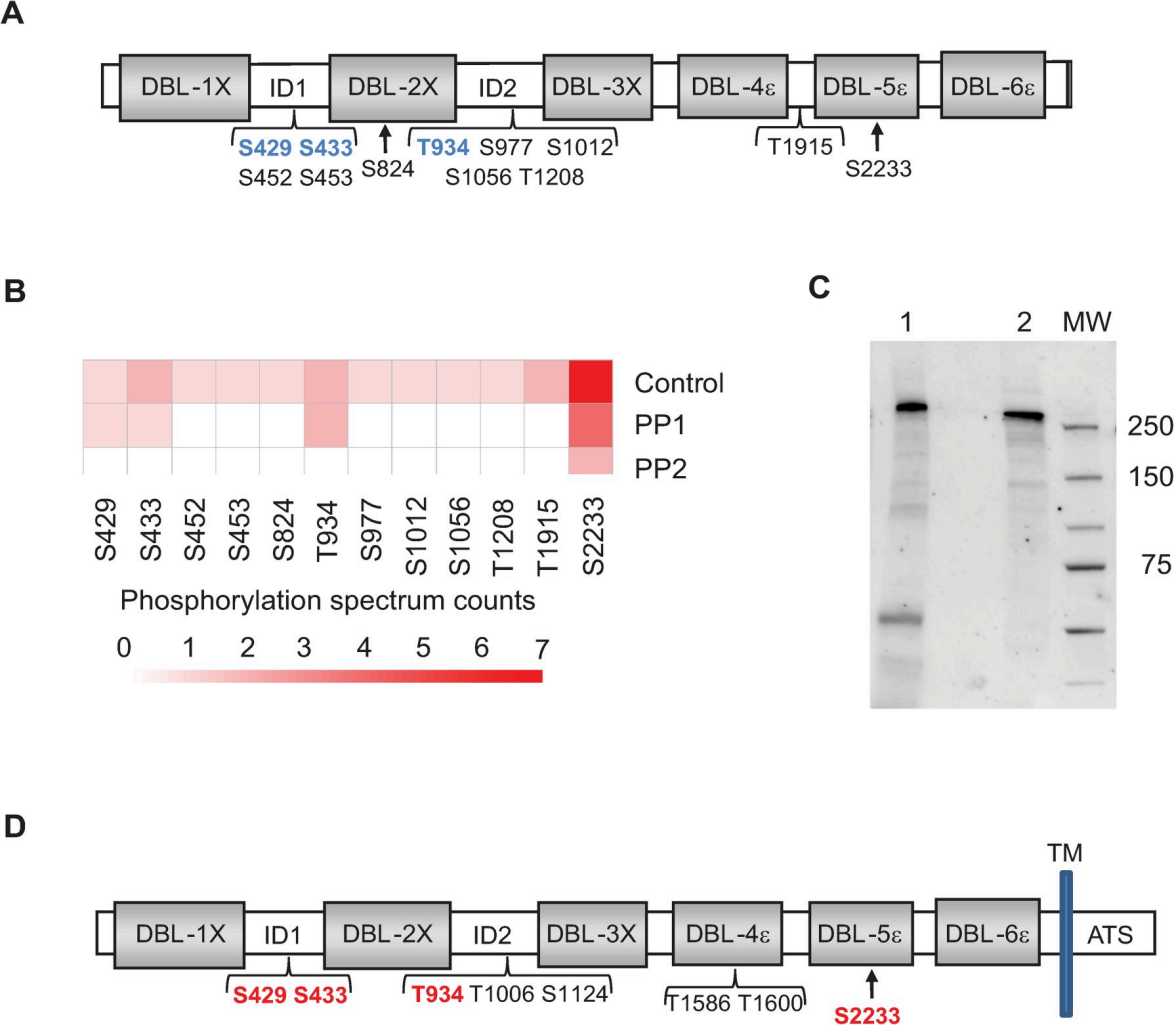
Identification by MS of VAR2CSA phosphosites. (A) Schematic view of rVAR2CSA identified phosphosites prior phosphatase treatment. Several phosphoresidues were identified in ID1, DBL2X, ID2, DBL4X, and ID4. Phosphoserine and phosphothreonine residues remaining after PP1 treatment but not after PP2a treatment are indicated in blue. (B) Spectral counting information related to the different phosphosites identified in the control rVAR2CSA, PP1-treated rVAR2CSA, and PP2a-treated rVAR2CSA are represented as a heat map. VAR2CSA was treated with 0.1 units PP1 or 0.1 units PP2a for 30 min. Reactions were stopped with phosphatases inhibitors, and phosphopeptides were analysed by MS. After PP1 treatment, 4 residual phosphosites were identified (S429, S433, T934, and S2233), whereas only 1 phosphosite could be detected (S2233) after PP2a treatment. Numerical values that underline the graph are shown in S3 Data. (C)Western blot analysis of the immunoprecipitated VAR2CSA protein. Lane 1: VAR2CSA immunoprecipitated protein from NF54 late-stage IEs. Lane 2: 100 ng of recombinant VAR2CSA was loaded as a positive control. (D) Schematic view of endogenous VAR2CSA-identified phosphosites. A total of 8 phosphoresidues were identified in ID1, ID2, DBL4X, and DBL5ε. Phosphoserine and phosphothreonine residues also present in the rVAR2CSA are indicated in red. DBL2X, Duffy-binding-like 2X; ID, interdomain; MS, mass spectrometry; MW, molecular weight; PP1, protein phosphatase 1; PP2a, protein phosphatase 2a; rVAR2CSA, recombinant VAR2CSA; VAR2CSA, variant surface antigen 2-CSA.

Having demonstrated that PP2a treatment decreases rVAR2CSA binding to CSA, we next assessed the phosphorylation status of rVAR2CSA produced in HEK293 cells. To identify VAR2CSA phosphosites, rVAR2CSA was resolved on a sodium dodecyl sulfate polyacrylamide gel electrophoresis (SDS-PAGE), and the band was excised for protein content analysis by liquid chromatography–tandem mass spectrometry (LC-MS/MS). This method allowed us to identify several phosphosites (Fig 2A, S1 Table). Phosphoserines and phosphothreonines were enriched within the interdomain regions and within the CSA-binding minimal region interdomain1-duffy binding like 2X-interdomain 2 (ID1-DBL2x-ID2) (Fig 2A), whereas no phosphotyrosine was detected.

In an attempt to identify phosphosites important for adhesion on CSA, we performed a MS analysis of rVAR2CSA phosphosites after PP1 or PP2a treatment. Integrity of rVAR2CSA protein has been verified after phosphatase treatment on an SDS-PAGE stained with coomassie blue (S2 Fig). After PP1 treatment, we identified 4 residual phosphoresidues (S429, S433, T934, and S2233), whereas only 1 phosphosite (S2233) was detected after PP2a treatment (Fig 2C).

No decrease of rVAR2CSA adhesion was observed after PP1 treatment, in contrast with the significant reduction after PP2a treatment (Fig 1D). Taken together, these results suggest that phosphoresidues S429, S433, and T934 may be important for adhesion. However, in contrast to S433 and T934 that could be unambiguously localised (Fig 2B), confidence in S429 localisation was poor due to low intensity spectra (S1 Table).

In order to confirm the presence of phosphoresidues in the endogenous native VAR2CSA expressed by the parasite, VAR2CSA was extracted from the membrane fraction of NF54 IEs at mid and late trophozoites stages and immunoprecipitated with an anti-VAR2CSA rabbit polyclonal antibody. Using an anti-VAR2CSA goat polyclonal antibody, western blot analysis performed on the immunoprecipitated material confirmed the presence of the endogenous VAR2CSA protein at around 350 kDa migrating slightly higher than the full-length extracellular rVAR2CSA (Fig 2C). After migration of the immunoprecipitated material on an SDS-PAGE gel and excision of the band followed by LC-MS/MS mass spectrometry, we confirmed that the endogenous parasite VAR2CSA is phosphorylated at 8 locations. Also, 4 phosphosites already identified in the rVAR2CSA were present in the endogenous VAR2CSA protein, including the phosphoserines S429 and S433 and the phosphothreonine T934 localised within the CSA-binding minimal region (ID1-DBL2x-ID2) that are potentially important for CSA adhesion (Fig 2D, S2 Table).

### Identification of rVAR2CSA phosphoresidues important for in vitro adhesion

**Fig 3:**
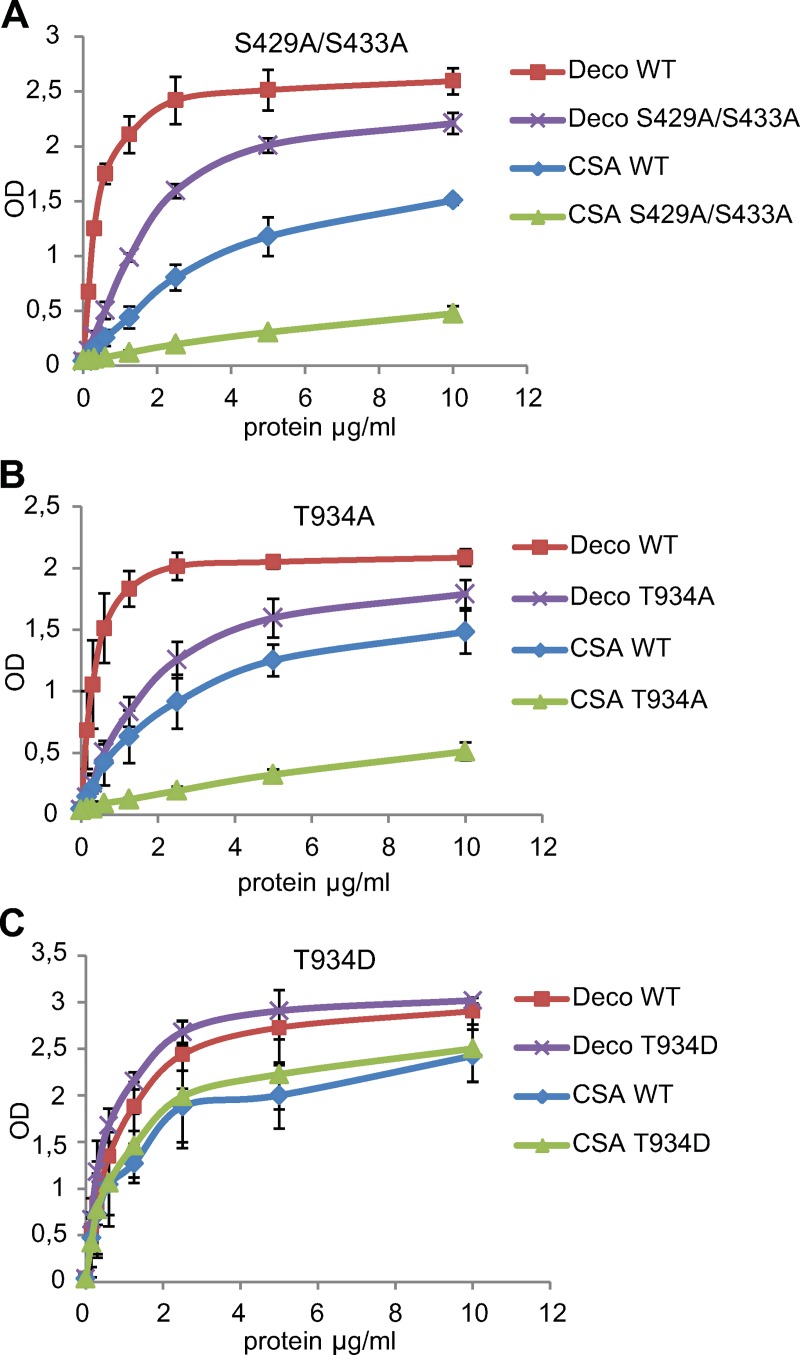
Adhesive properties of recombinant VAR2CSA WT, S429A/S433A, T934A, and T934D mutants. VAR2CSA WT, mutants S429A/S433A (A), T934A (B), and T934D (C) were assayed by ELISA at different protein concentrations for in vitro binding to CSA- or decorin-coated ELISA plates. Increasing concentrations of recombinant proteins at serial dilutions of 0.156 to 10 μg/mL were added to wells previously coated with BSA, CSA, or decorin. Error bars correspond to SD between 3 independent experiments. Each experiment was performed in triplicate. Numerical values that underline the graphs are shown in S4 Data. BSA, bovine serum albumin; CSA, chondroitin sulfate A; Deco, decorin; ELISA, enzyme-linked immunosorbent assay; OD, optical density; VAR2CSA, variant surface antigen 2-CSA; WT, wild type.

In order to investigate the role of phosphoresidues S429, S433, and T934 in VAR2CSA in vitro adhesion, we assessed the binding of VAR2CSA recombinant proteins (expressed in HEK293 cells and purified as described in the methods section) in which the potential phosphosites are substituted with an alanine residue. No significant difference in protein purity and size was observed on SDS-PAGE after coomassie staining between the mutant and wild-type proteins (S3 Fig). We then tested the functional consequences of the above mutations using an enzyme-linked immunosorbent assay (ELISA) performed on both decorin and CSA. A significant reduction in binding to both decorin and CSA was observed with the VAR2CSA double mutant S429A/S433A and T934A single mutant (Fig 3A and 3B). In order to verify that the binding reduction was due to the absence of a phosphorylated residue and not to an impairment of the overall 3D structure of VAR2CSA, we generated a new mutant recombinant protein in which T934 is substituted by aspartic acid that mimics phosphorylation at this position. Contrary to the alanine substitution (T934A), the binding to both decorin and CSA of the T934D mutant was similar and even slightly higher compared with that of the wild type (Fig 3C), indicating that this substitution is able to mimic the phosphothreonine and that phosphorylation of this residue is important for in vitro adhesion.

### Expression of VAR2CSA T934A on the IEs surface

**Fig 4:**
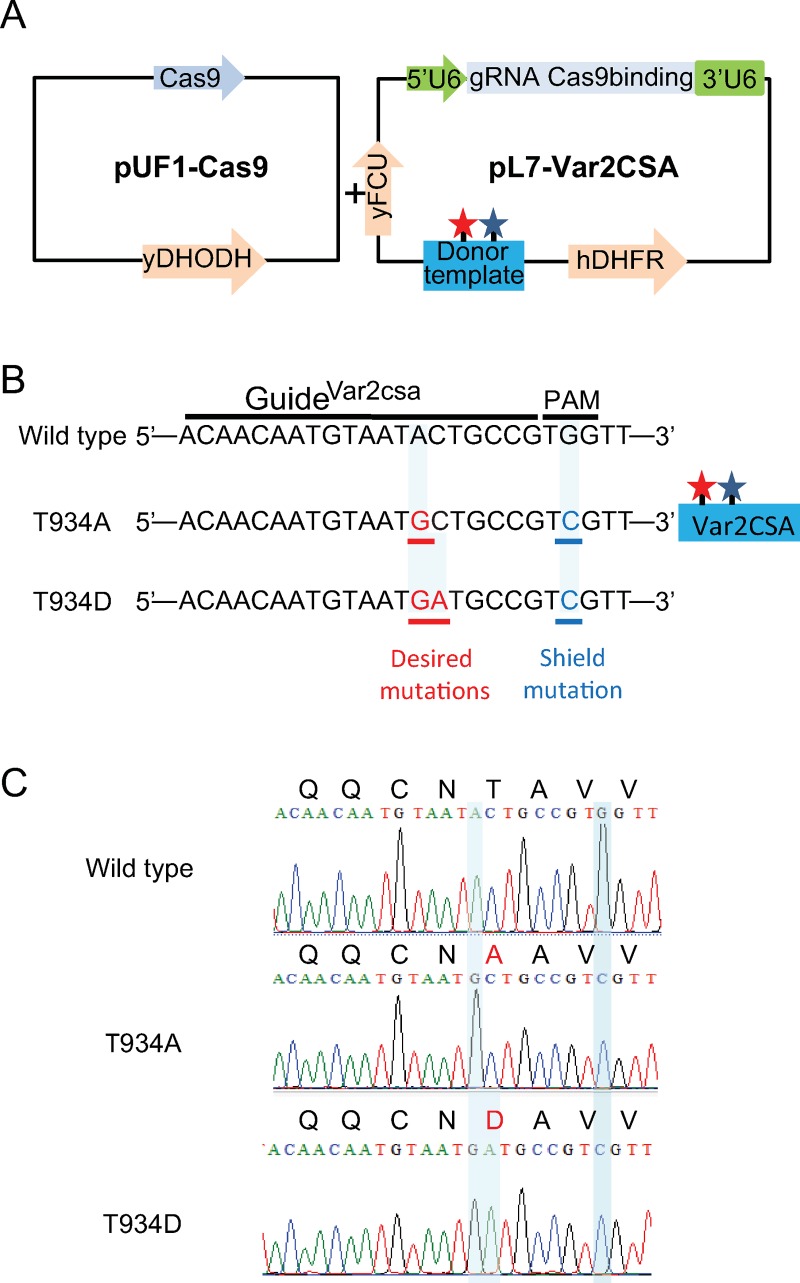
Strategy used for targeted *P*. *falciparum* VAR2CSA genome editing using sgRNA:Cas9 system. (A) pUF1-Cas9 and pL7Var2CSA were used for transfection in NF54 CSA strain. The Cas9 endonuclease bearing NLS is expressed in the pUF1-Cas9 vector carrying the ydHODH drug cassette. pL7-Var2CSA episome carries both sgRNA VAR2CSA and the donor DNA under the drug-selectable marker (hdhfr). sgRNA is expressed from the *P*. *falciparum* U6 snRNA polymerase III promoter (5ʹ U6). The donor DNA carries the desired mutations (red star) and shield mutation (blue star). (B) sgRNA VAR2CSA targeted sequences recognized by Cas9. The 20 nucleotides’ guide and PAM sequences are indicated. Modified locus to create the desired mutations T934A and T934D as well as the shield mutations are shown. (C) Chromatograms showing VAR2CSA locus in NF54CSA wild type and in transgenic NF54CSA T934A and T934D clones sequence analyses. Nucleotide substitutions and amino acids changes in VAR2CSA locus are highlighted. Cas9, CRISPR-associated protein-9 nuclease; CSA, chondroitin sulfate A; gRNA, guide RNA; hDHFR, human dihydrofolate reductase; NLS, nuclear localisation signal; PAM, protospacer-adjacent motif; pL7-Var2CSA, variant surface antigen 2-CSA; pUF1, RNA binding protein Pumilio; sgRNA, single guide RNA; snRNA, small nuclear RNA; VAR2CSA, variant surface antigen 2-CSA; ydHODH, yeast dihydroorotate dehydrogenase gene; yFCU, uridyl-phosphoribosyltransferase.

In order to confirm the importance of VAR2CSA phosphorylation in IEs cytoadhesion, targeted gene editing using CRISPR/Cas9 was used to generate transgenic parasites expressing endogenous VAR2CSA in which S429/S433 and T934 are replaced with alanine residues. Engineered *Streptococcus pyogenes* endonuclease Cas9 was expressed in the RNA binding protein pUF1-Cas9 episome that also carries a drug-selectable marker which gives resistance to (5-methyl[1,2,4]triazolo[1,5-a]pyrimidin-7-yl)naphthalen-2-ylamine (DSM1), a dihydroorotate dehydrogenase inhibitor (Fig 4A) [31]. We generated plasmids pl7-Var2CSA carrying the WR99210 drug-selectable marker hDHFR bearing a guide RNA targeting the NF54 VAR2CSA locus and a donor template containing the desired mutations and a silent shield mutation to protect the mutated locus from caspase 9 repeated breaks (Fig 4A and 4B and S4 Fig). Although parasites appeared under double DSM1 and WR99210 selection, no S429A/S433A transgenic parasites were obtained after 3 different transfection attempts. However, T934A transgenic parasites were obtained after 4 weeks under DSM1 and WR99210 double selection. Sequencing of genomic DNA from the uncloned T934A transgenic parasite population confirmed the presence of the desired nucleotide substitution at the *var2csa* endogenous locus. Several clones were obtained by limiting dilution, one of which (clone D1) was fully sequenced for VAR2CSA integrity and for the presence of the expected mutations (Fig 4C) prior to phenotypic characterisation.

**Fig 5:**
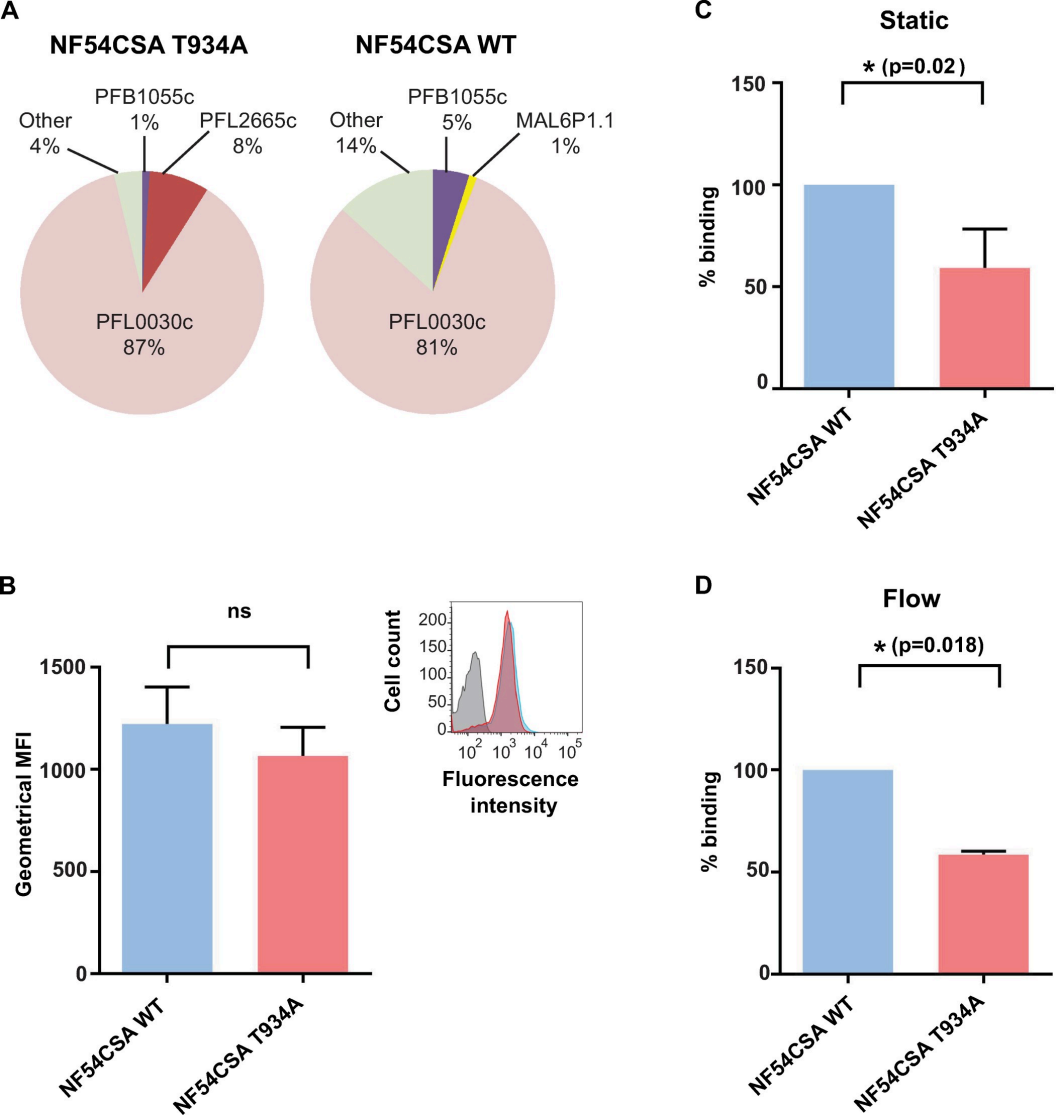
Characterisation of transgenic NF54CSA T934A parasites. (A) VAR2CSA transcription profile of wild-type and T934A NF54CSA IEs after reselection on VAR2CSA antibodies. Transcriptional levels of each *var* genes were normalised with the housekeeping gene, seryl-tRNAtransferase. (B) Flow cytometry analysis of wild-type and T934A NF54CSA IEs after panning with anti-VAR2CSA antibodies. CSA-selected IEs were labelled with anti-VAR2CSA antibodies. Geometric means of fluorescence intensities of 2 independent experiments and corresponding standard deviation are indicated. (C) Static cytoadhesion assay of wild-type and T934A NF54CSA IEs. The effect of T934A mutation on IEs binding was assessed in static assay on CSA-coated petri dishes. Numbers of NF54CSA T934A IEs attached were counted in 5 random fields, and results were expressed as percentage binding compared with wild-type NF54CSA IEs (100% binding) from 3 independent experiments. Blue and red histograms correspond, respectively, to the wild-type and transgenic parasite strains. (D) Effect of VAR2CSA T934A mutation on IEs binding was assessed in a flow-based adhesion assay. IEs were flowed over decorin-coated microslides. A shear stress of 0.05 Pa was applied for 10 min. Numbers of adherent IEs were counted in 6 positions, and results were expressed as percentage binding compared with wild-type NF54CSA IEs (100% binding). Blue and red histograms correspond, respectively, to the wild-type and transgenic parasite strains. Numerical values that underline the graphs are shown in S5 Data. CSA, chondroitin sulfate A; IEs, infected erythrocytes; MFI, mean fluorescence intensity; NF54CSA, *Plasmodium falciparum* isolate NF54 selected for CSA adhesion; ns, not significant; VAR2CSA, variant surface antigen 2-CSA; WT, wild type.

Erythrocytes infected with the parental NF54 wild-type clone or the T934A mutant clone D1 were selected for VAR2CSA expression by successive rounds of panning on anti-VAR2CSA polyclonal antibodies. The *var* gene transcription profile shows a progressive enrichment in *var2csa* transcript after each panning round (S5 Fig). Panning was pursued until the same *var2csa* transcription profile was observed in 2 consecutive panning rounds (around 85% of IEs expressing the *var2csa* transcript normalised with housekeeping genes; Fig 5A). No significant difference was observed in surface reactivity between both VAR2CSA-selected populations, as measured by flow cytometry using purified rabbit polyclonal anti-VAR2CSA immunoglobulin G (IgG) (Fig 5B). Taken together, these results indicate that VAR2CSA was expressed at similar levels on the surface of erythrocytes infected with the wild-type parental line and the transgenic T934A clone D1.

### Effect of the VAR2CSA T934A mutation on IEs cytoadhesion

The cytoadhesion behaviour of erythrocytes infected with the wild-type line or the T934A clone D1 was comparatively assessed under a static binding assay, in which CSA is immobilised on plastic, and a flow binding assay, in which decorin is coated on microfluidic chips. Static assay revealed that the VAR2CSA mutation T934A significantly decreases clone D1 IEs adhesion (*p* = 0.0206; Fig 5C). Under flow, at a shear stress of 0.05 Pa, erythrocytes infected with the T934A clone displayed a significantly weaker capacity to cytoadhere to decorin, compared with erythrocytes infected with wild-type parasites (*p* = 0.0179; Fig 5D). This demonstrates that the T934A mutation causes a decrease in the binding of IEs under static and flow conditions.

### Characterisation of the transgenic VAR2CSA T934D IEs

**Fig 6:**
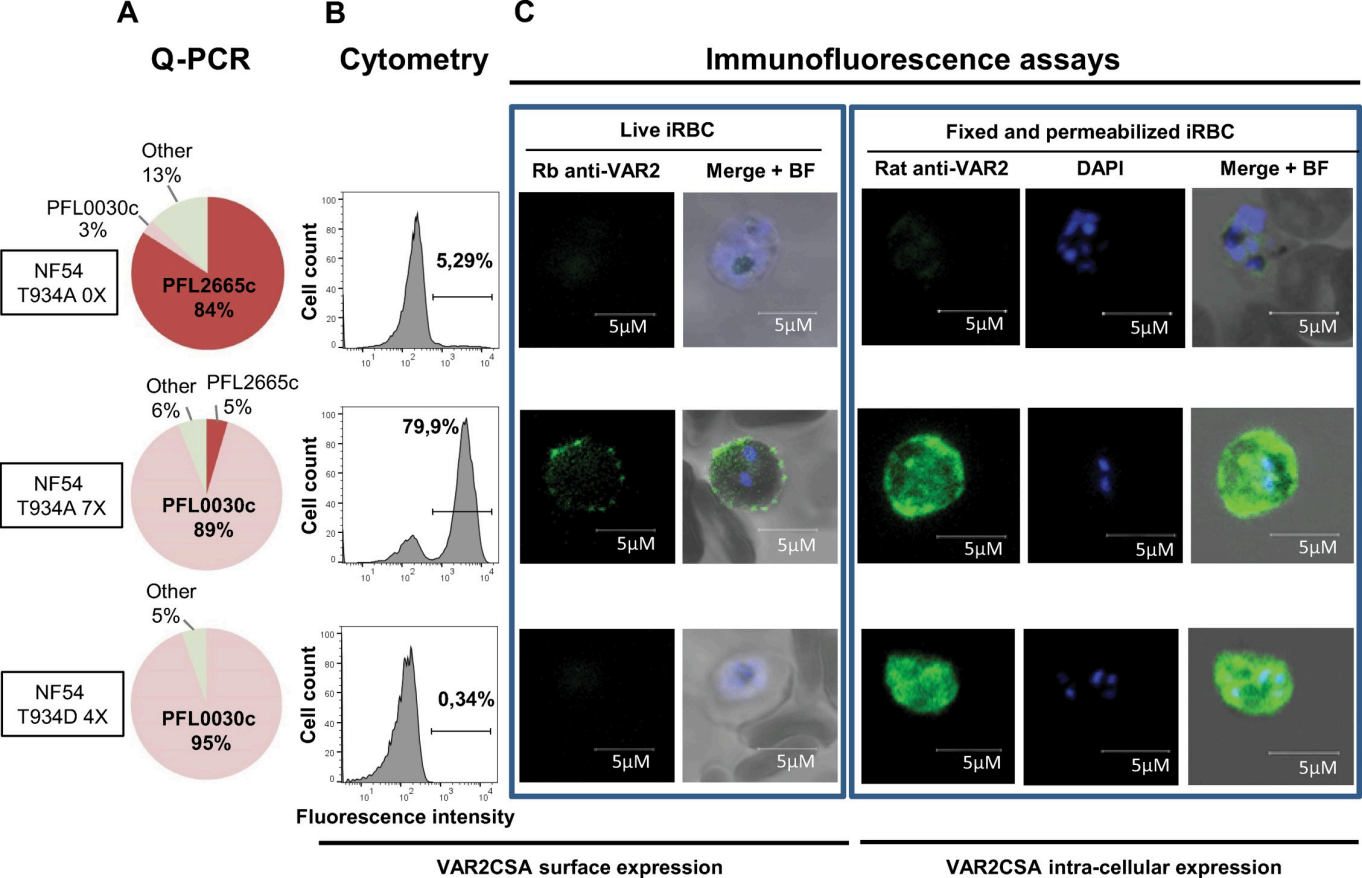
VAR2CSA expression and localisation of transgenic NF54CSA T934A and T934D parasites. (A) *Var2csa* transcription profile of NF54 T934A clone D1 before (NF54 T934A 0X) and after selection for VAR2CSA expression (NF54 T934A 7X) and NF54 T934D clone G10 after selection for VAR2CSA expression (NF54 T934D 4X). (B) VAR2CSA surface expression by flow cytometry analysis. (C, left panels) VAR2CSA cell surface staining of live parasites examined by immunofluorescence using a rabbit anti-VAR2CSA immunopurified antibody followed by a Goat Alexa Fluor 488 anti-rabbit. The nucleus was stained using Hoesch dye. (Right panels) VAR2CSA staining on fixed and permeabilised smears after labelling with a rat serum anti-VAR2CSA followed by a goat Alexa Fluor anti-rat. The nucleus was observed with DAPI. Scale bar represents 5 μM. BF, bright field; iRBC, infected red blood cell; NF54, *Plasmodium falciparum* strain NF54; NF54CSA, *Plasmodium falciparum* strain NF54 selected for CSA adhesion; Q-PCR, quantitative polymerase chain reaction; Rb, rabbit; DAPI, 4',6-diamidino-2-phénylindole; VAR2CSA, variant surface antigen 2-CSA.

We then decided to investigate the effect of the T934D mutation on IE cytoadhesion. Using the CRISPR/Cas9 targeted gene editing technology, we generated transgenic parasites expressing VAR2CSA in which T934 is replaced by aspartic acid. Using the same strategy as described above for generating the T934A mutant parasite, several clones were obtained by limiting dilution, and one clone (G10) was fully sequenced for VAR2CSA integrity and for the presence of the expected mutations (Fig 4C). Although the *var* gene transcription profile indicates that *var2CSA* is expressed at 95% in the T934D mutant parasite (Fig 6A), no VAR2CSA surface expression was detected by cytometry using a specific rabbit anti-VAR2CSA antibody (Fig 6B). Using the same antibody, immunofluorescence studies performed on live parasites confirm the absence of VAR2CSA on the surface of the transgenic T934D IEs panned 4 times for VAR2CSA surface expression in contrast to a punctuated surface staining on the surface of the T934A IEs panned 7 times for VAR2CSA surface expression (T934A 7X). The T934A before panning and not expressing VAR2CSA (Fig 6A) was negative for VAR2CSA surface expression as expected (Fig 6B and 6C). To further investigate the cellular localisation of VAR2CSA, parasites were fixed, permeabilised, and stained with a specific rat anti-VAR2CSA serum [32]. Although the T934A 7X used as a positive control showed an internal localisation as well as a peripheral labelling, the transgenic T934D IEs only revealed an intense intracellular labelling indicating that the protein is not exported to the red cell membrane surface.

## Discussion

In the context of PM, VAR2CSA exposed on the surface of IEs mediates adhesion to the CSA placental receptor and stands today as the leading vaccine candidate aiming to protect pregnant women living in malaria endemic areas against the severe clinical outcomes of PM [33,34]. Although recent progress has been achieved in both the biochemical and the biophysical characterisation of the extracellular region of VAR2CSA, notably with respect to the high affinity CSA-binding site in the ID1-ID2a encompassing region within the DBL1X-3X segment of VAR2CSA [21–23,35], no data on PTM that may regulate IEs cytoadhesion to CSA are available. Many lines of evidence concur that the phosphorylation of cell surface proteins is an important post-translational event in the regulation and modulation of many physiological processes, including cellular ligands interactions [24,26].

**Fig 7:**
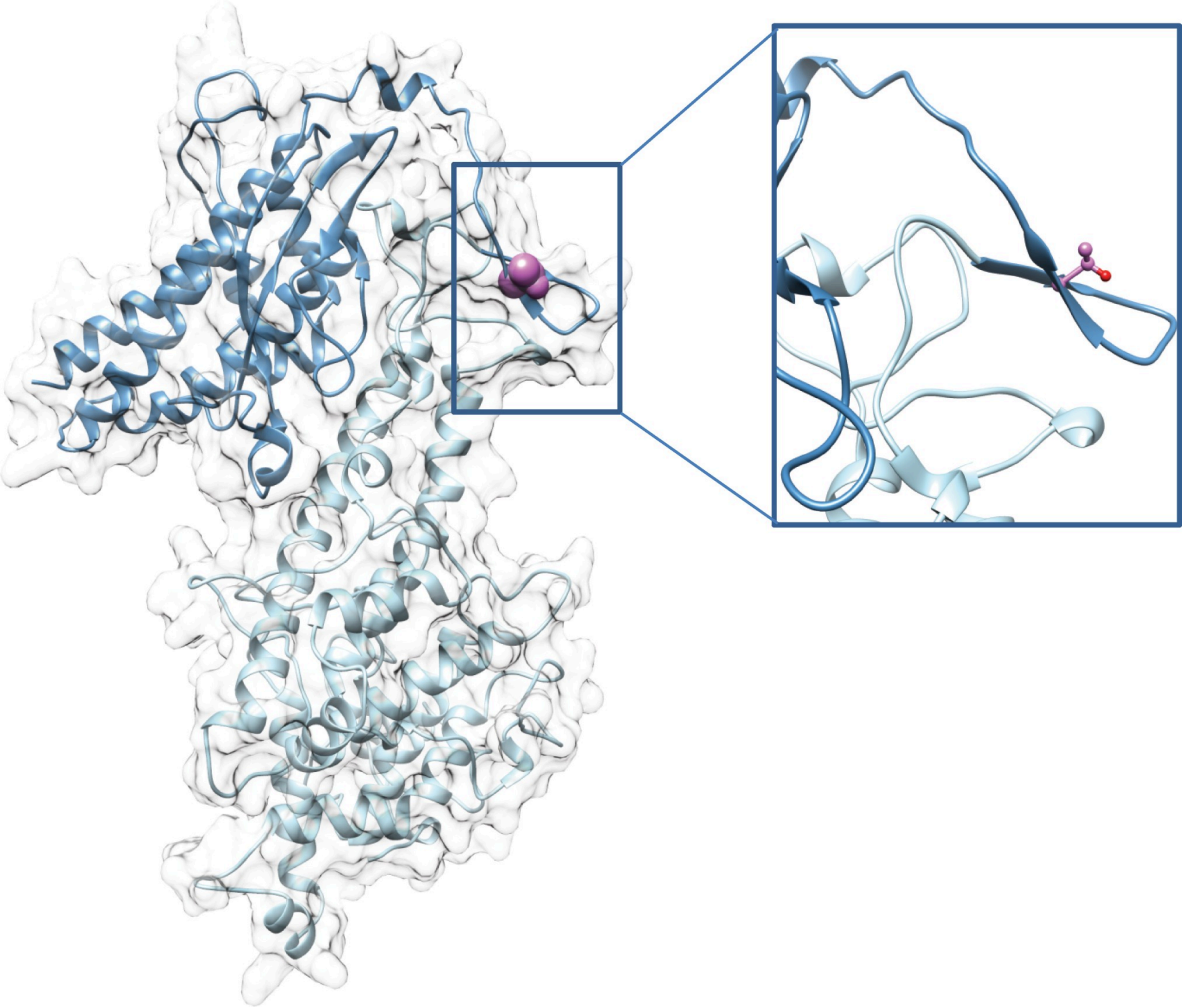
3D model of DBL2X-CIDR double domain of VAR2CSA. (Left) Global view of the DBL2X-CIDR model in ribbon representation. (Right) Zoom on the T934 residue. Light blue, DBL2X domain; dark blue, ID2 or CIDR; pink, T934 side-chain represented as spheres (left) or ball and stick (right). CIDR, cysteine-rich interdomain region; DBL2X-CIDR, Duffy-binding-like 2X-cystein-rich interdomain region; ID2, interdomain 2; VAR2CSA, variant surface antigen 2-CSA.

Here, we provide direct evidence that phosphorylation of VAR2CSA expressed at the IEs surface is linked to enhanced adhesive mechanisms associated to malaria pathogenesis. We showed that treatment with PP1 and PP2a phosphatases leads to a drastic reduction of IEs cytoadhesion to BeWo placental cells and to immobilised CSA, in both static and flow conditions, suggesting that VAR2CSA extracellular region is phosphorylated, and that the phosphorylation status influences binding properties of VAR2CSA. We further showed that recombinant VAR2CSA produced in HEK293 cells is indeed phosphorylated and that PP2a (but not PP1) treatment has a drastic effect on the ability of the recombinant protein to bind to CSA. Inactivity of the PP1 enzyme in this experiment was ruled out, because PP1 did in fact dephosphorylate several residues of rVAR2CSA, as assessed by MS analysis (Fig 2B). Phosphoproteomic studies on both recombinant and endogenous VAR2CSA allowed us to identify important phosphorylation sites located within the ID1-ID2a high affinity CSA-binding region. Indeed their substitution to alanine significantly reduced the interaction of recombinant VAR2CSA with CSA in vitro. In particular, the T934 mutation induces a drastic decrease in CSA binding, whereas a phosphomimetic mutant (T934D) exhibits a similar binding to that of the wild-type protein. The 3D structural model of the DBL2X-ID2 region confirms that T934 located within ID2 domain also called cysteine-rich interdomain region pregnancy associated malaria (CIDRpam), based on sequence homologies to CIDR domains of other PfEMP1 members, is solvent exposed, suggesting that mutating this position to an alanine or aspartic acid should not alter the overall structure of the VAR2CSA protein (Fig 7).

Furthermore, genome-wide analysis of 46 VAR2CSA sequences from isolates and strains originating from different parts of the world indicate that although S429 and S433 are highly conserved, T934 is present in all the VAR2CSA sequences (S6 Fig). Using the CRISPR/Cas9 gene editing tool, we confirmed that the T934A mutation strongly impairs IEs cytoadhesion to CSA in static as well as in flow conditions. We thus provide direct evidence that phosphorylation plays a major role in VAR2CSA interaction with CSA.

We were able to obtain the T934D mutant parasite, however, no VAR2CSA surface expression was detected by cytometry and live IFA while an intense intracellular labelling was observed, indicating that the protein is not exported to the red cell membrane surface. These results could imply that mimicking the T934 phosphorylation impacts the trafficking of VAR2CSA to the red cell surface. *P*. *falciparum* exports a number of proteins into the host cell and the parasitophorous vacuole, which separates the parasites from the host-cell cytoplasm. Many of the proteins exported in the parasitophorous vacuole and the host-cell cytoplasm are phosphorylated [36]. Our results then suggest that phosphorylation of T934 could occur after the protein reaches either the red cell surface or the host-cell cytoplasm; an early phosphorylation may thus modify the export of the protein.

Although PP1 treatment has no effect on the binding of recombinant VAR2CSA to CSA, an inhibitory effect was observed on the adhesion behaviour of various VAR2CSA-expressing strains to CSA and to BeWo cells. Several hypotheses can be proposed to explain this observation. First, phosphorylated parasite or red blood cells (RBC) proteins other than VAR2CSA could form a complex with VAR2CSA and contribute to CSA adhesion or stabilise the complex. It has been proposed that other malaria antigens are also found on the surface of CSA-binding IEs and might be implicated in IEs adhesion to placental cells [37,38]. Second, it is possible that additional VAR2CSA phosphoresidues important for CSA binding and targeted by PP1 were not phosphorylated in HEK293 cells but may be phosphorylated by RBC or *P*. *falciparum* kinases not present in HEK cells. Indeed, MS analysis of the endogenous VAR2CSA identified phosphosites not present in the rVAR2CSA (Fig 2). Also, it has to be noted that the identification of all the phosphorylation sites of native VAR2CSA is technically challenging due to the low amounts of PfEMP1on the IEs’ surface [39] and then to the difficulty to extract and recover enough material to obtain high sequence coverage of the protein. In line with this hypothesis, VAR2CSA as well as PFD0020c (a PfEMP1 variant distinct from VAR2CSA) were found to be phosphorylated in their extracellular region [40]. However, only one serine (S1518) localised at the end of the DBL3X domain of VAR2CSA and not found phosphorylated in our study was identified.

VAR2CSA is the leading vaccine candidate against PM. Furthermore, it was recently reported that the CSA-binding region of VAR2CSA conjugated with therapeutics can strongly inhibit in vivo tumour growth and efficiently retrieves circulating tumour cells by binding to CSA present on cancer cells [41,42]. Our findings demonstrate that phosphorylation of VAR2CSA is important for CSA adhesion and therefore may have a regulatory role in cell adhesion and placental sequestration.

These observations are opening novel therapeutic strategies aiming to target parasite or host kinases to inhibit IEs sequestration in the placenta. The next step is to identify the kinases phosphorylating those residues. Between 85 and 99 genes encoding protein kinase–related enzymes have been identified in the *P*. *falciparum* genome, and at least 2 parasite kinases (*P*. *falciparum* Casein Kinase 1 (PfCK1) and *P*. *falciparum* tyrosine kinase like 2 (PfTKL2)) have been shown to be secreted to the extracellular medium [43–45]. Furthermore, several members of a unique family of parasite putative kinases containing around 19 members, collectively called phenylalanine-isoleucine-lysine-lysine-motif-containing kinase (FIKK), have been shown to be exported to the RBC cytoplasm and have been implicated in IEs remodelling [46]. Indeed, erythrocytes infected with a line that does not express FIKK4.2 were significantly less rigid and less adhesive than those infected with wild-type parasites, despite similar levels of PfEMP1 on the red blood cell surface [47].

Using the NetPhos 3.1 online phosphorylation prediction tool [48], numerous putative VAR2CSA phosphosites, including S433 but neither S429 nor T934, are predicted to be phosphorylated (S3 Table). CK2 is one of the kinases predicted to phosphorylate S433. In addition to phosphorylating a variety of intracellular substrates, Human CK2 is an ectokinase able to phosphorylate the extracellular domains of various proteins, such as CD98, a transmembrane glycoprotein and regulator of multiple functions including extracellular signaling, cell adhesion, polarity, and cell–cell interactions [24]. VAR2CSA could be phosphorylated by Human CK2 or possibly by its *Plasmodium* orthologue (PfCK2), which is essential for the blood stage parasites [49] and known to control various cell parasite processes such as invasion and chromatin dynamics [50,51].

Further studies are needed to identify the kinases mediating VAR2CSA phosphorylation and to determine whether phosphorylation is also playing a role in cytoadhesion implicating other PfEMP1 variants, for example, those mediating interaction with ligands such as cluster of differentiation 36 (CD36), intercellular adhesion molecule-1 (ICAM-1), or endothelial protein C receptor (EPCR).

In conclusion, we provide direct evidence that the extracellular region of VAR2CSA is phosphorylated and that this phosphorylation plays a major role in VAR2CSA interaction with the placental receptor CSA. This clearly links VAR2CSA phosphorylation to PM pathogenesis and reveals a novel putative level of regulation of host–parasite interactions. These findings advance our understanding of how VAR2CSA adhere to CSA and have important implications not only for future strategies aiming to protect pregnant women and their babies against the morbidity of PM but also in designing better cancer diagnostic and therapeutic tools.

## Materials and methods

### *P*. *falciparum* in vitro culture

*P*. *falciparum* FCR3CSA, NF54CSA, and 7G8CSA strains expressing VAR2CSA were cultured under standard conditions in O+ RBC in RPMI 1640 containing L-glutamine (Invitrogen France) supplemented with 5% Albumax I, 5% Human plasma, 1× hypoxanthin, and 20 μg/mL gentamicin [52]. Parasitemia was routinely monitored by a thin blood smear fixed with 100% methanol for 1 min before staining in 10% vol/vol Giemsa (Sigma-Aldrich) in phosphate-buffered saline for 15 min. Genomic DNA extracted from parasite cultures was regularly tested for Mycoplasma contamination (look out Mycoplasm PCR detection kit; Sigma). Parasite cultures were routinely selected by gelatin flotation using Plasmion (Fresenius Kabi France) to maintain knob-positive parasites [53].

### Phosphatase assays

Phosphatase assays using PP1 and PP2a (Millipore France) were performed either with recombinant full-length VAR2CSA protein or with VAR2CSA-expressing IEs in phosphatase buffer (40mM Tris HCl [pH 8.4], 34mM MgCl2, 4mM EDTA, 2mM DTT, 0.05mg/ml BSA) for 30 min at 30°C [54].

### *P*. *falciparum* cytoadhesion assays

CSA-binding IEs phenotypes were verified on CSA and CD36 receptors immobilised on plastic petri dishes as previously described [55]. Static IE cytoadhesion to the human trophoblastic BeWo cell line was assessed as previously described [55]. BeWo cells were grown at 37°C in F12 medium (Gibco) supplemented with 10% FBS and L-glutamine on 8-wells culture slides precoated with Poly L-lysine solution 0.01% in PBS (10 μl/ well). Media was changed every day until cells grew to confluence. After 48 h, cells were gently washed with PBS and blocked with PBS BSA 1% 1 h at RT. 5 × 10^7^ VarioMACS (Miltenyi Biotec France) purified IEs at mid and/or late trophozoite stages treated for 30 min at 30°C with PP1 or PP2a phosphatases (0.1 units) or the enzymes buffer as a control were applied at RT on BeWo cells for 1 h. Unbound IEs were washed off 3 times with PBS, and bound cells were fixed with glutaraldehyde 2% and counted microscopically using a microscope Nikon Eclipse Ti microscope with a 10 X objective. Adherent IEs were counted on 5 different fields. IEs incubated with enzyme buffer were used as positive control in these experiments to represent 100% binding.

Static IE cytoadhesion assays on CSA (Sigma France, C8529) were performed as previously described [55]. Briefly, 10 μl of CSA (1mg/ml) in PBS or 1% BSA in PBS (negative control) were spotted on a petri dish overnight at 4°C in a humidified chamber. The spots were washed twice with PBS and blocked with PBS BSA 1% for 1 h at RT. VarioMACS purified mid and late stages IEs were added to the spots and incubated at RT for 1 h. Unbound cells were gently washed away with PBS. Adherent IEs were fixed with 2% glutaraldehyde and counted from 5 fields in duplicate spots with the same microscope as above. Results are expressed as the percent of inhibition compared to 100% binding of positive control.

Flow cytoadhesion assays were performed using Vena8 Endothelial+ biochips (Cellix Ltd, Dublin, Ireland) precoated in each channel with 10 μl of CSA-carrying decorin proteoglycan (Sigma, D8428) at 50 μg/ml in PBS overnight at 4°C in a humid chamber. The channels were blocked with PBS 2% BSA for 1 h at RT. VarioMACS purified mid and/or late-stage IEs treated or not with PP1 or PP2a phosphatases were then flowed over receptor-coated slides at a shear stress of 0.05 Pa mimicking wall shear stress in placenta [29]. After washing, serial pictures of adherent cells in different positions in the channel were taken with an Axio Observer Z1 microscope, X 10 objective.

### Expression and purification of full-length VAR2CSA recombinant proteins

Full-length 3D7 DBL1X-6ε extracellular region wild type, S429A/S433A, T934A, and T934D mutants were expressed in HEK293F (embryonic human kidney from Life Technologies France) as soluble proteins secreted in the culture medium and purified on a His Trap Ni affinity column, followed by a ion exchange chromatography (SP Sepharose) and a gel filtration chromatography (Superdex 200) as previously described [23]. Site-directed mutagenesis to introduce the required mutations were carried out using the Quick change II XL kit (Agilent France) according to the manufacturer’s protocol. Presence of mutations was verified by sequencing before protein expression (GATC). All oligonucleotides used in the present study are listed in S4 Table.

### ELISA binding assay

96-wells ELISA plates were coated overnight at 4°C with 100 μL per well of 5 μg/mL decorin (Sigma France, Ref D8428) or 50 μg∕mL of CSA (Sigma France, Ref C8529) in PBS (Gibco, NaCl 150 mM [pH 7.2]).

Wells were blocked with 150 μL of dilution buffer per well (PBS 1% BSA) 2 h at 37°C. After removal of the blocking solution, serial dilutions of the recombinant VAR2CSA proteins (wild type and mutated) in PBS 1% BSA, 0.05% Tween20 from 10 μg/ml to 0.156 μg/ml were added per well and incubated for 2 h at 37° C. For phosphatases studies, 2.5 μg/ml of VAR2CSA was incubated with various concentrations of PP1, PP2a, or phosphatase buffer for 30 min at 30°C prior adding to the wells. After washing 3 times with PBST (PBS plus 0.05% Tween 20), 100 μL anti-His HRP conjugated antibody (diluted 1/2000 in PBST) was added to each well and incubated for 1 h at 37°C. After washing 3 times with PBST, the reaction was revealed with 100 μL per well of substrate (TMB (3,3',5,5'-tetramethylbenzidin; Biorad France)) until saturation was reached. Interaction was related to the absorbance monitored at 655 nm.

### CRISPR/Cas9 genome editing

T934A mutation effect on adhesion was assessed in vivo by introducing this mutation into the parental NF54 line using the previously described CRISPR/Cas9 system [31]. Plasmids pL7-Var2CSA and pUF1-Cas9 were cotransfected into the parental cell line. The Cas9 protein is expressed in the pUF1-Cas9 episome continuously maintained using the yDHODH drug-selectable marker. pL7-Var2CSA episome is continuously maintained using the hdhfr selection. The plasmid pL7-Var2CSA was obtained by insertion of the homology region of Var2CSA into the pl7 vector (donor) and the 20 nucleotides guide DNA sequence (sgRNA). The sgRNA targeting sequence is followed by the PAM, TGG used by the Cas9 enzyme to provoke a specific DNA double strand break. Using primers (P1) and (P2), a PCR product encoding the var2CSA gRNA targeting sequence for Cas9 was cloned in the pL7 vector previously digested by BtgZ1. The VAR2CSA donor fragment of approximatively 400 bp containing the Sac2/Afl2 ends plus the 15 bp necessary for infusion cloning (Clontech) in the pL7 vector was amplified by PCR using the external primers P3 and P4. Primers P5 and P6 were used in combination with the P3 and P4 primers to introduce the desired T934A mutation plus the shield mutation in the donor. Primers P7 and P8 were used in combination with the same external primers (P3 and P4) to generate the T934D mutation plus the shield mutation that avoids repeated cleavages by Cas9 enzyme. All PCR products were amplified with high fidelity polymerase Pfu Ultra II Fusion DNA polymerase (Agilent Technologies) and with genomic DNA from NF54 parental line. To avoid potential off target mutations, we performed a DNA motif pattern search (PlasmoDB) by blasting genomic regions flanking the protospacer-adjacent motif (PAM) on the whole *P*. *falciparum* genome. We did not find any homologous sequences beside the gene of interest. Primers for sgRNA and the donor templates are indicated in S4 Table.

NF54CSA parasites were transfected at the ring stages by electroporation. For transfection, 50 μg of each plasmid pL7-Var2CSA and pUF1 Cas9 were used, ethanol precipitated and resuspended in sterile TE. Both drugs: WR99210 (2.6 nM) and DSM1, 5-methyl[1,2,4]triazolo[1,5-a]pyrimidin-7-yl)naphthalen-2-ylamine (2 μM) were added 20 h post-transfection and applied every day. Appearance of resistant parasites was monitored daily.

### VAR2CSA selection by successive panning on anti VAR2CSA polyclonal antibodies

VAR2CSA-expressing IEs were selected on purified rabbit anti-VAR2CSA antibodies. One hundred microliters of Dynabeads protein G (Life Technologies France) were coated with 20 μg of polyclonal anti-VAR2CSA purified IgG for 10 min, washed twice with PBS, and blocked with PBS 1% BSA for 10 min at RT. VarioMACS purified IEs at trophozoites stages were allowed to bind to the coated beads for 20 min at 37°C. Unbound IEs were washed out with PBS. Bound IEs were recovered using a magnetic device and were brought back into culture. The day after panning, the beads were removed from the cultures, and RNA was extracted for *var* gene transcription profile study. Five rounds of panning were needed to obtain a population in which the *var* gene transcription profile did not vary anymore reflecting a complete selection of *var2CSA* expressing IEs.

### Var gene expression analysis by qPCR

RNA from NF54 wild-type and transgenic synchronized ring stages parasites was extracted with Trizol (Invitrogen France) following the manufacturer’s instructions (RNeasy Minikit, Qiagen France). cDNA synthesis was performed by random primers after DNase I treatment (TURBO DNase, Ambion France) using the Super Script III First Stand Synthetis system (Invitrogen France). Primers pairs to assess *var* gene expression have been described previously [9]. Quantitative real time PCR reactions were performed on a CFX 96 thermocycler (Biorad France). Transcriptional level of each *var* gene was normalised using the housekeeping control gene seryl tRNA transferase (PlasmoDB: PF07_ 0073).

### VAR2CSA surface expression analysis by flow cytometry analysis

VarioMACS (Miltenyi Biotec France) purified IEs at mid and late trophozoites stages were resuspended in PBS 0.2% BSA and counted. For each assay, 0.5 × 10^6^ IEs were washed in PBS and incubated with 50 μl of purified rabbit polyclonal anti-VAR2CSA IgG diluted 1/100 in PBS 0.2% BSA for 1 h at RT. IEs were washed twice with PBS and resuspended in 100μl of PE conjugated goat anti-rabbit IgG diluted 1/100 in PBS for 30 min at RT. After washing twice in PBS 0.2% BSA, IEs were resuspended in paraformaldehyde 2% in PBS and kept at 4°C overnight in darkness. Cells were then washed twice with PBS and analysed by flow cytometry using a BD FACScanto II flow cytometer (Becton Dickinson France). with the Flow Jo 10.0 software. Parasite nuclei was stained with Topro3 (1/10,000 dilution). The results are expressed as the geometric mean fluorescence intensities.

### Extraction and immunoprecipitation of native VAR2CSA from the IEs surface

VarioMACS (Miltenyi Biotec) purified NF54 IEs at mid and late trophozoites stages expressing VAR2CSA were lysed in cold NETT buffer (NaCl 150mM, EDTA 5mM, Tris 50mM [pH 8], Triton X-100 1%). After centrifugation at 4°C for 30 min, the pellet containing the membrane proteins was resuspended in Tris saline buffer containing 2% of SDS. The supernatant containing the solubilised native VAR2CSA was then incubated with a polyclonal rabbit anti-VAR2CSA [56] coupled to magnetic protein G Dynabeads (Invitrogen France). After several washes, the beads were resuspended in Laemli buffer containing DTT (Invitrogen France). All buffers were supplemented with phosphatase inhibitors (Phos Stop, Roche France) and protease inhibitors (complete EDTA free cocktail; Roche France)

### Western blot

Ten percent of the immunoprecipitated material as well as 100 ng of the recombinant full-length recombinant VAR2CSA were loaded and migrated on a 4% to 15% gradient gel to assess for the presence of immunoprecipitated VAR2CSA prior MS analysis. After turbo transfert (Biorad France), the membrane was blocked with Tris buffer saline 0.1% Tween 20, 3% BSA (TBST-B) for 1 h and then incubated with a goat polyclonal anti-VAR2CSA (gift from M. Wahlgren) diluted 1/1000 in TBST-B for 2 h. After washes, a secondary anti-goat HRP conjugated antibody (Abliance France) diluted 1/1000 in TBST-B was added. The antigen-antibody complex was then detected using Clarity ECL Substrate (Biorad France) according to the manufacturer’s recommendations and monitored using the ChemiDoc MP Imaging System (Biorad France). Protein quantification was performed using Image Lab Software.

### MS analysis

Purified or immunoprecipitated native VAR2CSA were separated by SDS-PAGE on a 10% polyacrylamide gel (Biorad France), which was then stained with Biosafe coomassie (Invitrogen France) and destained in H_2_O. Gel band corresponding to VAR2CSA proteins were entirely extracted and proteins were in-gel digested using trypsin. After peptide extraction from gel slices, phosphopeptide enrichments were performed on homemade titania tips [57]. For LC-MS/MS analysis, peptides were resuspended in 2% Acetonitrile; 0.1% Formic Acid. MS analysis was performed using an Orbitrap Elite (Thermo Fischer Scientific Switzerland) or an Orbitrap Fusion Lumos coupled to an Ultimate 3000 RSLC nano-UPLC system (Dionex USA). Database search was performed using Mascot (Matrix Science) and SEQUEST in Proteome Discoverer version 1.3 against a home database consisting of the VARCSA sequence inserted into the Uniprot *Escherichia coli* background proteome. Immunoprecipitated endogenous VAR2CSA data analysis was performed using SEQUEST HT in Proteome Discoverer version 2.2 against the *P*. *falciparum* reference proteome. Searches were performed with trypsin cleavage specificity and ion mass tolerance of 10 ppm for the precursor and 0.5 Da for the fragments. Carbamidomethylation was set as a fixed modification, whereas oxidation (M), acetylation (Protein N-term), and phosphorylation (STY) were considered as variable modifications. Data were further processed and inspected in the Scaffold 3 software (Proteome Software). Phosphorylation site localisation and post-translational modifications (PTM) spectral counting was investigated using Scaffold PTM software (Proteome Software) for the purified recombinant VAR2CSA. Phosphorylation site localisation of the immunoprecipitated endogenous VAR2CSA was computed using phosphoRS [58] in Proteome Discoverer version 2.2.

### 3D structure modelling

The structure of the region spanning DBL2X-CIDR (ID2) double domain of VAR2CSA has been modelled with 90% coverage (645 amino acids) and 100% confidence using Phyre2 programme [59]. The model was generated based on the X-ray structure of nts-DBL1α-CIDRγ domain of PfEMP1 from varo3 strain (PDB 2YKO) sharing 23.6% sequence identity with DBL2X-CIDR sequence.

### Immunofluorescence

Live mature-stage parasites were incubated 45 min with a rabbit anti-VAR2CSA immunopurified antibody 1/500. After 2 washes in PBS/1% BSA, the parasites were labelled 45 min on ice with a goat anti-Rabbit (H+L) Alexa Fluor 488 (Invitrogen France; Ref: A11034) diluted 1/1000 on ice. After Hoesch (Invitrogen France) staining, the parasites were observed with a LSM 700 Zeiss confocal microscope under a 63X immersion oil objective.

Immunofluorescence was performed on fixed asexual mature stages parasites. Briefly, smeared parasites were fixed in cold acetone/methanol (90/10) for 15 min. After blocking with 1% BSA in PBS for 1 h, the slides were incubated with a rat anti-VAR2CSA serum (dilution 1:500) followed by incubation with goat anti-Rat (H+L) Alexa Fluor 488 (Invitrogen; Ref: A11006) diluted 1/1000. After washes, the slides were mounted with Fluoromount plus DAPI and observed with a LSM 700 Zeiss confocal microscope under a 63X immersion oil objective.

## Supporting information

S1 Fig Effect of okadaic acid, a PP2a specific inhibitor, on binding of rVAR2CSA. rVAR2CSA was pretreated with PP2a or with PP2a + okadaic acid (500nM), and then binding to decorin was assayed as described in Materials and methods. Numerical values that underline the graphs are shown in S2 Data. PP2a, protein phosphatase 2a; rVAR2CSA, recombinant VAR2CSA.
(TIF)

S2 Fig VAR2CSA phosphatases treated samples for MS analysis. rVAR2CSA and rVAR2CSA PP1 or PP2a treated purified proteins were loaded on a SDS-PAGE in reducing conditions and stained with coomassie. MS, mass spectrometry; PP1, protein phosphatase 1; PP2a, protein phosphatase 2a; SDS-PAGE, sodium dodecyl sulfate polyacrylamide gel electrophoresis; rVAR2CSA, recombinant VAR2CSA; VAR2CSA, variant surface antigen 2-CSA.
(TIF)

S3 Fig Purified WT and mutant recombinant proteins. Purified VAR2CSA WT, S429A/S433A, T934A, and T934D recombinant proteins expressed in HEK293 cells were loaded on a SDS-PAGE in reducing conditions and coomassie stained. HEK293, human embryonic kidney 293; SDS-PAGE, sodium dodecyl sulfate polyacrylamide gel electrophoresis; VAR2CSA, variant surface antigen 2-CSA; WT, wild type.
(TIF)

S4 Fig Strategy used for targeted *P. falciparum* VAR2CSA genome editing using sgRNA:Cas9 system. (A) pUF1‐Cas9 and pL7Var2CSA were used for transfection in NF54 CSA strain. The Cas9 endonuclease bearing NLSs is expressed in the pUF1‐Cas9 vector carrying the ydHODH drug cassette. pL7‐Var2CSA episome carries both sgRNA VAR2CSA and the donor DNA under the drug‐selectable marker (hdhfr). sgRNA is expressed from the *P*. *falciparum* U6 snRNA polymerase III promoter (5′ U6). The donor DNA carries the desired mutations (red star) and shield mutation (blue star). (B) sgRNA VAR2CSA targeted sequences recognized by Cas9. The 20 nucleotide guides and PAM sequences are indicated. Modified locus to create the desired mutations S429A/S433A as well as the shield mutations are shown. Cas9, CRISPR-associated protein-9 nuclease; NF54, *Plasmodium falciparum* isolate NF54; NLS, nuclear localisation signal; PAM, protospacer-adjacent motif; pL7-Var2CSA, variant surface antigen 2-CSA; pUF1, RNA binding protein Pumilio; sgRNA, single guide RNA; snRNA, small nuclear RNA; VAR2CSA, variant surface antigen 2-CSA; ydHODH, yeast dihydroorotate dehydrogenase gene; hdhfr, human dihydrofolate reductase.
(TIF)

S5 Fig NF54CSA T934A var gene expression profiling after 1, 3, and 5 pannings. NF54CSA T934A clone was selected for VAR2CSA expression by successive pannings on anti‐VAR2CSA polyclonal antibodies. The *var* genes expression profile shows a progressive enrichment in VAR2CSA transcripts after 1, 3, and 5 panning rounds. NF54CSA, *Plasmodium falciparum* isolate NF54 selected for CSA adhesion; VAR2CSA, variant surface antigen 2-CSA; T934A, threonine934 alanine.
(TIF)

S6 Fig Alignment of 46 NTS-ID2a VAR2CSA sequences from isolates and strains originating from different parts of the world. Ser429, Ser433, and Thr934 are boxed in red. Genome-wide analysis of 46 NTS-ID2a VAR2CSA sequences from isolates and strains originating from different parts of the world indicate that although Ser429 and Ser433 are highly conserved, T934 is present in all the VAR2CSA sequences. NTS-ID2a, N terminal sequence–interdomain 2a; Ser429, serine429; Ser433, serine433; Thr934, threonine934; VAR2CSA, variant surface antigen 2-CSA.
(PDF)

S1 Table Summary of all phosphorylation sites identified in rVAR2CSA produced in HEK293 cells. Identified phosphopeptides are reported together with their localisation score. HEK293, human embryonic kidney 293; rVAR2CSA, recombinant VAR2CSA.
(XLSX)

S2 Table Summary of all phosphorylation sites identified in endogenous NF54 VAR2CSA by MS. Identified phosphopeptides are reported together with their localisation score. MS, mass spectrometry; NF54, *Plasmodium falciparum* isolate NF54; VAR2CSA, variant surface antigen 2-CSA.
(XLSX)

S3 Table VAR2CSA NetPhos 3.1 online phosphorylation prediction results. Putative VAR2CSA phosphosites predicted to be phosphorylated. VAR2CSA, variant surface antigen 2-CSA.
(XLSX)

S4 Table Oligonucleotides used in the present study. Primers for site-directed mutagenesis and for CRISPR/Cas9 genome editing are listed. CRISPR/Cas9, clustered regularly interspaced short palindromic repeats/ caspase 9.
(XLSX)

S1 Data Excel spreadsheet containing, in separate sheets, the underlying numerical data for Fig 1A, 1C and 1D. (XLSX)

S2 Data Excel file containing the underlying numerical data for S1 Fig. (XLSX)

S3 Data Excel file containing the underlying numerical data for Fig 2B. (XLSX)

S4 Data Excel spreadsheet containing, in separate sheets, the underlying numerical data for Fig 3A, 3B and 3C. (XLSX)

S5 Data Excel spreadsheet containing, in separate sheets, the underlying numerical data for Fig 5B, 5C and 5D. (XLSX)

## Abbreviations

ATS: acidic terminal segment
BeWo: Human placenta choriocarcinoma cells
CD36: cluster of differentiation 36
CIDR: cysteine-rich interdomain region
CIDRpam: cysteine-rich interdomain region pregnancy associated malaria
CK2: Casein Kinase 2
PfCK1: P. falciparum Casein Kinase 1
PfTKL2: P. falciparum tyrosine kinase like 2
CRISPR/Cas9: clustered regularly interspaced short palindromic repeats/CRISPR-associated protein-9 nuclease
CSA: chondroitin sulfate A
DBL: Duffy-binding-like
DSM1: (5-methyl[1,2,4]triazolo[1,5-a]pyrimidin-7-yl)naphthalen-2-ylamine
ELISA: enzyme-linked immunosorbent assay
EPCR: endothelial protein C receptor
FIKK: phenylalanine-isoleucine-lysine-lysine-motif-containing kinase
HEK: Human embryonic kidney
ICAM-1: intercellular adhesion molecule-1
ID: interdomain
IEs: infected erythrocytes
IgG: immunoglobulin G
KAHRP: knob-associated histidine-rich protein
LC-MS/MS: liquid chromatography tandem mass spectrometry
MS: mass spectrometry
NTS: N-terminal segment
PAM: protospacer-adjacent motif; hdhfr, human dihydrofolate reductase
sgRNA: single guide RNA
snRNA: small nuclear RNA
PfEMP1: P. falciparum erythrocyte membrane protein 1
PM: placental malaria
PP1: protein phosphatase 1
PP2a: protein phosphatase 2a
PTM: post-translational modification
pUF1: RNA-binding protein Pumilio
RBC: red blood cell
rVAR2CSA: recombinant VAR2CSA
SDS-PAGE: sodium dodecyl sulfate polyacrylamide gel electrophoresis
VARC: ATS of PfEMP1
VAR2CSA: variant surface antigen 2-CSA
yDHODH: yeast dihydroorotate dehydrogenase
BSA: bovine serum albumin
OD: optical density
deco: decorin
WT: wild type
MFI: mean fluorescence intensity
Q-PCR: quantitative polymerase chain reaction
BF: bright field
Rb: rabbit
DAPI: 4',6-diamidino-2-phénylindole
yFCU: uridyl-phosphoribosyltransferase
NF54: Plasmodium falciparum strain NF54
NF54CSA: Plasmodium falciparum strain NF54 selected for CSA adhesion
FCR3: Plasmodium falciparum strain FCR3
FCR3CSA: Plasmodium falciparum strain FCR3 selected for CSA adhesion
7G8: Plasmodium falciparum strain 7G8
7G8CSA: Plasmodium falciparum strain 7G8 selected for CSA adhesion
