# Supplementary figures and images for: Phosphorylation of the VAR2CSA extracellular region is associated with enhanced adhesive properties to the placental receptor CSA
Source: PLoS Biol. 2019 Jun 10;17(6):e3000308. doi: 10.1371/journal.pbio.3000308 (PMC6586358; doi:10.1371/journal.pbio.3000308)

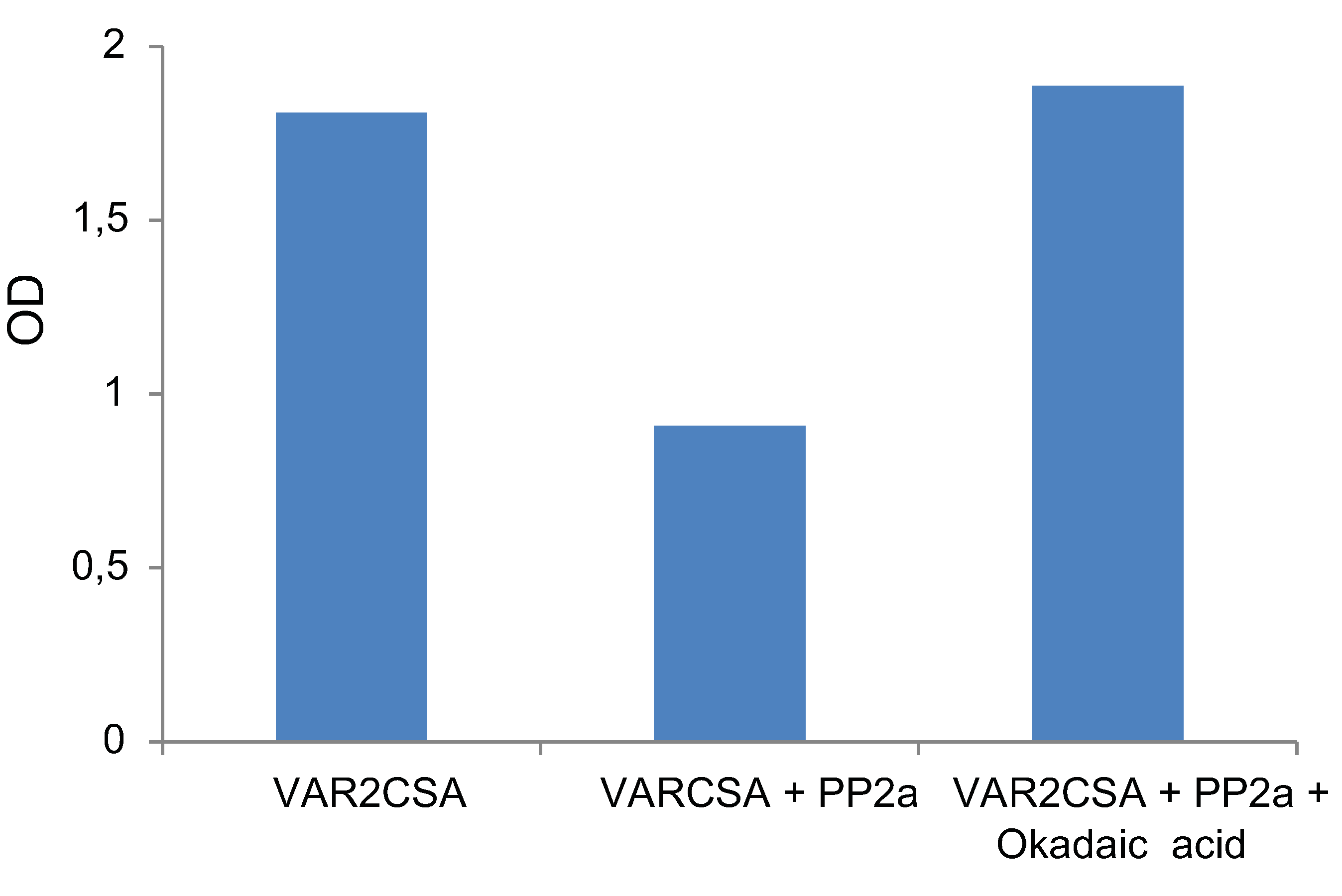

Supplement: S1 Fig — rVAR2CSA was pretreated with PP2a or with PP2a + okadaic acid (500nM), and then binding to decorin was assayed as described in Materials and methods. Numerical values that underline the graphs are shown in S2 Data. PP2a, protein phosphatase 2a; rVAR2CSA, recombinant VAR2CSA. (TIF) [file pbio.3000308.s001.tif]

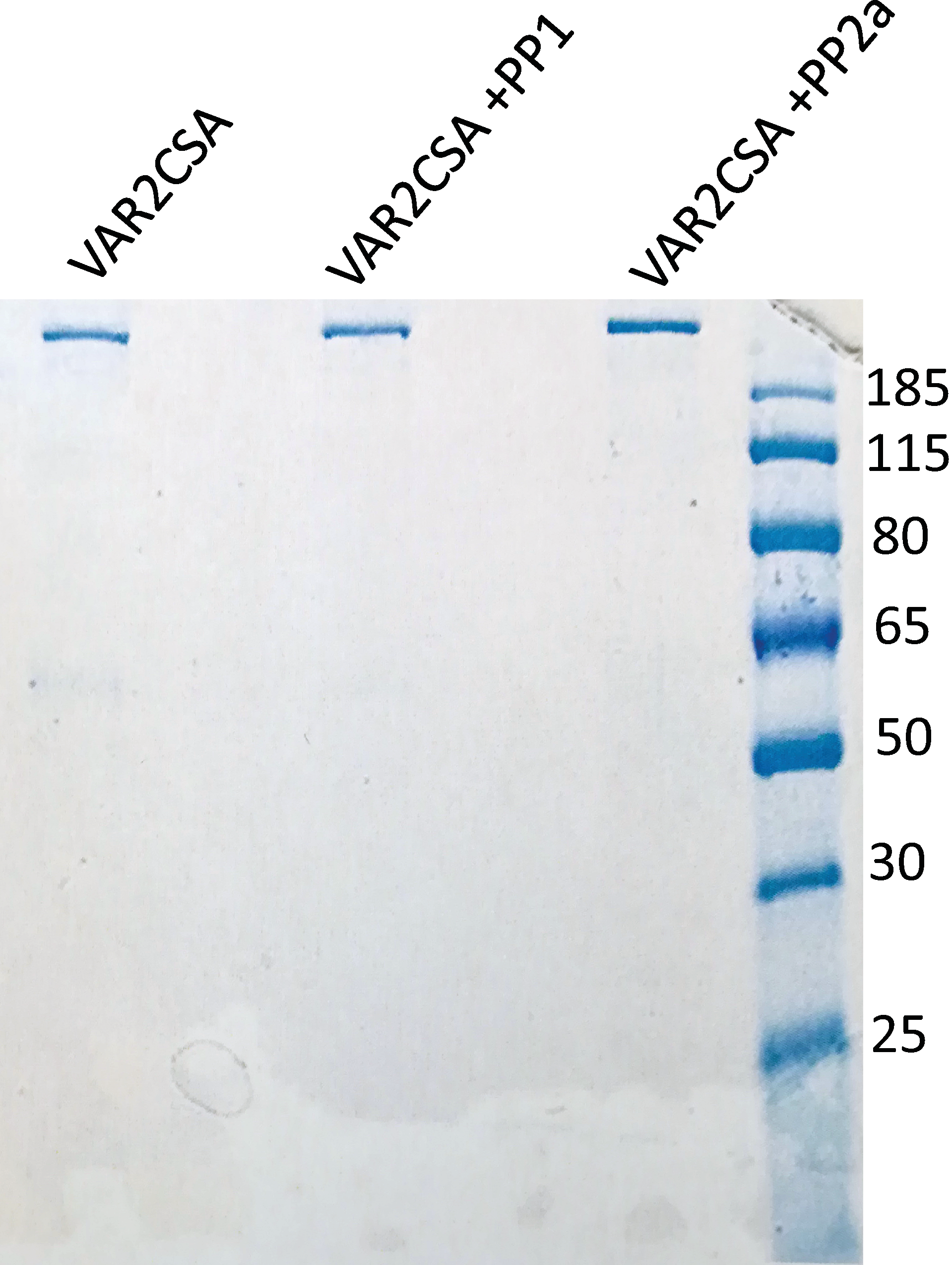

Supplement: S2 Fig — rVAR2CSA and rVAR2CSA PP1 or PP2a treated purified proteins were loaded on a SDS-PAGE in reducing conditions and stained with coomassie. MS, mass spectrometry; PP1, protein phosphatase 1; PP2a, protein phosphatase 2a; SDS-PAGE, sodium dodecyl sulfate polyacrylamide gel electrophoresis; rVAR2CSA, recombinant VAR2CSA; VAR2CSA, variant surface antigen 2-CSA. (TIF) [file pbio.3000308.s002.tif]

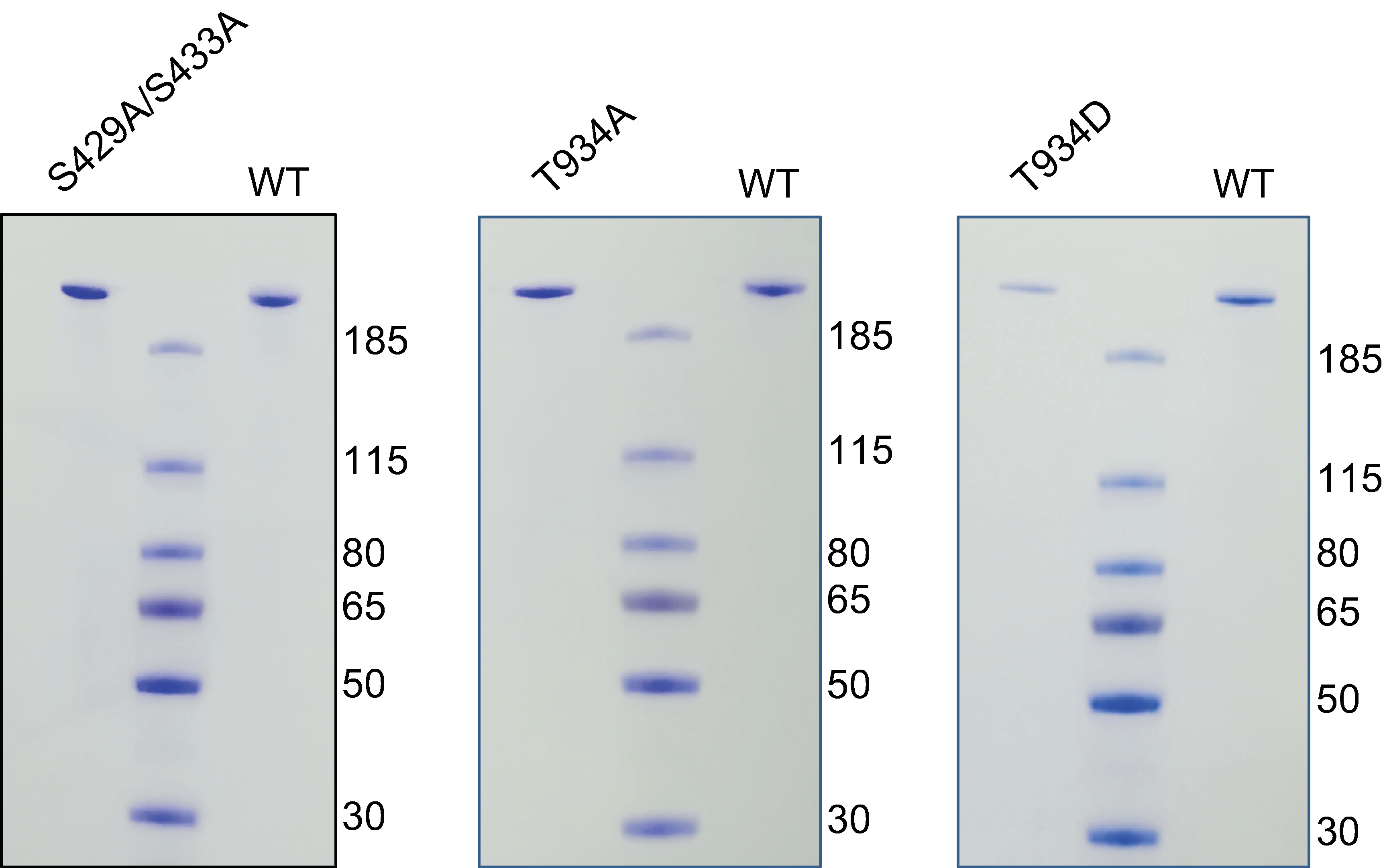

Supplement: S3 Fig — Purified VAR2CSA WT, S429A/S433A, T934A, and T934D recombinant proteins expressed in HEK293 cells were loaded on a SDS-PAGE in reducing conditions and coomassie stained. HEK293, human embryonic kidney 293; SDS-PAGE, sodium dodecyl sulfate polyacrylamide gel electrophoresis; VAR2CSA, variant surface antigen 2-CSA; WT, wild type. (TIF) [file pbio.3000308.s003.tif]

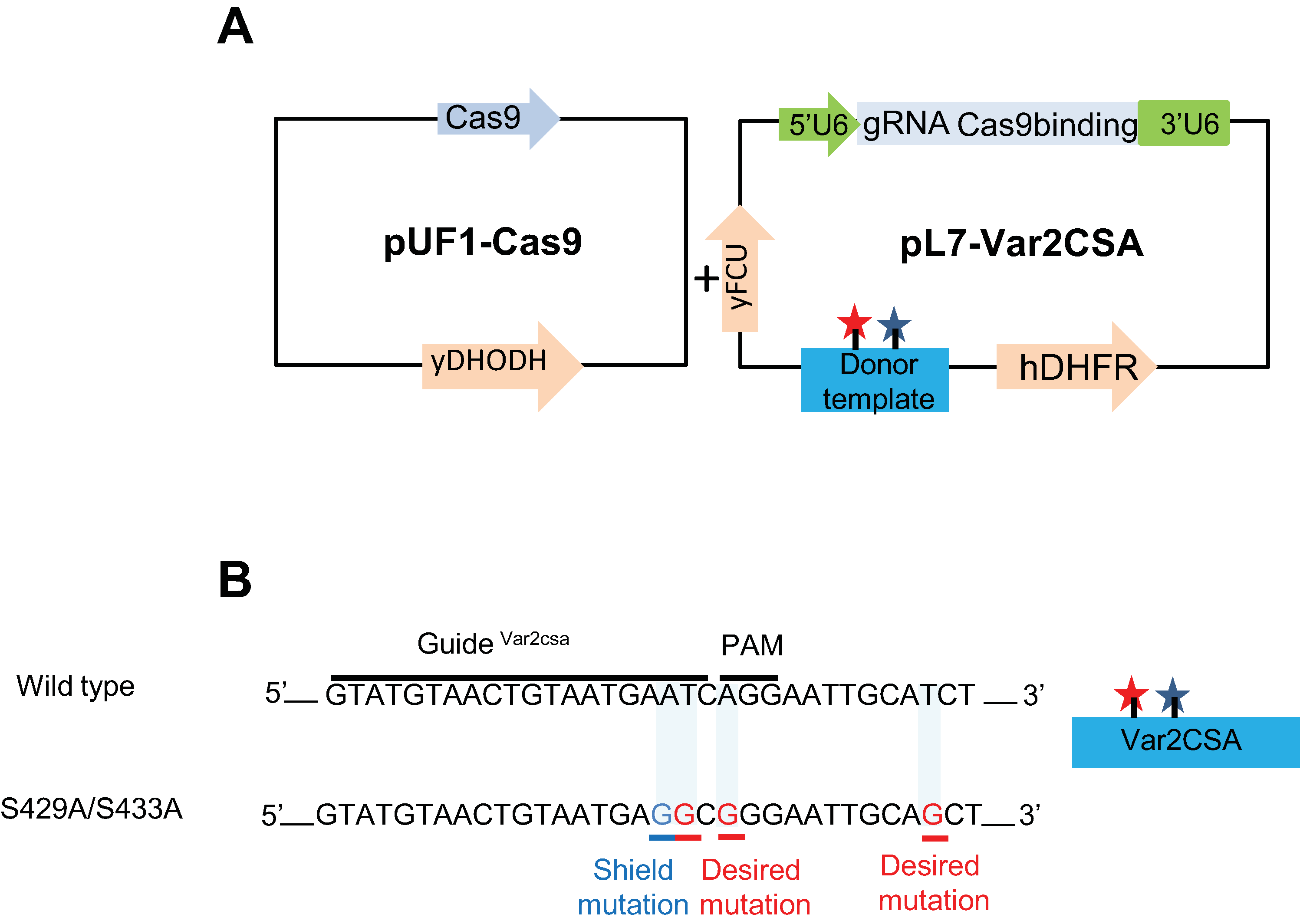

Supplement: S4 Fig — (A) pUF1‐Cas9 and pL7Var2CSA were used for transfection in NF54 CSA strain. The Cas9 endonuclease bearing NLSs is expressed in the pUF1‐Cas9 vector carrying the ydHODH drug cassette. pL7‐Var2CSA episome carries both sgRNA VAR2CSA and the donor DNA under the drug‐selectable marker (hdhfr). sgRNA is expressed from the P. falciparum U6 snRNA polymerase III promoter (5′ U6). The donor DNA carries the desired mutations (red star) and shield mutation (blue star). (B) sgRNA VAR2CSA targeted sequences recognized by Cas9. The 20 nucleotide guides and PAM sequences are indicated. Modified locus to create the desired mutations S429A/S433A as well as the shield mutations are shown. Cas9, CRISPR-associated protein-9 nuclease; NF54, Plasmodium falciparum isolate NF54; NLS, nuclear localisation signal; PAM, protospacer-adjacent motif; pL7-Var2CSA, variant surface antigen 2-CSA; pUF1, RNA binding protein Pumilio; sgRNA, single guide RNA; snRNA, small nuclear RNA; VAR2CSA, variant surface antigen 2-CSA; ydHODH, yeast dihydroorotate dehydrogenase gene; hdhfr, human dihydrofolate reductase. (TIF) [file pbio.3000308.s004.tif]

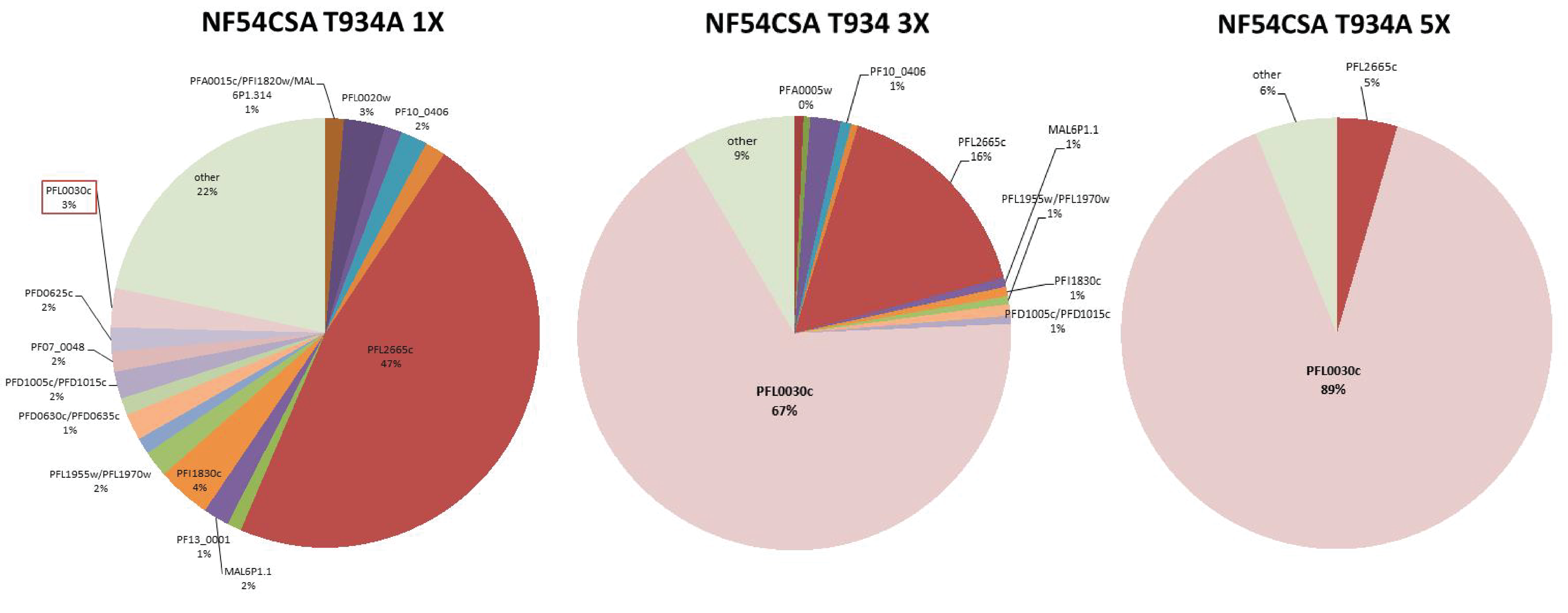

Supplement: S5 Fig — NF54CSA T934A clone was selected for VAR2CSA expression by successive pannings on anti‐VAR2CSA polyclonal antibodies. The var genes expression profile shows a progressive enrichment in VAR2CSA transcripts after 1, 3, and 5 panning rounds. NF54CSA, Plasmodium falciparum isolate NF54 selected for CSA adhesion; VAR2CSA, variant surface antigen 2-CSA; T934A, threonine934 alanine. (TIF) [file pbio.3000308.s005.tif]
