# Supplementary material for: Phosphorylation of the VAR2CSA extracellular region is associated with enhanced adhesive properties to the placental receptor CSA
Source: PLoS Biol. 2019 Jun 10;17(6):e3000308. doi: 10.1371/journal.pbio.3000308 (PMC6586358; doi:10.1371/journal.pbio.3000308)
Supplement: S6 Fig — Ser429, Ser433, and Thr934 are boxed in red. Genome-wide analysis of 46 NTS-ID2a VAR2CSA sequences from isolates and strains originating from different parts of the world indicate that although Ser429 and Ser433 are highly conserved, T934 is present in all the VAR2CSA sequences. NTS-ID2a, N terminal sequence–interdomain 2a; Ser429, serine429; Ser433, serine433; Thr934, threonine934; VAR2CSA, variant surface antigen 2-CSA. (PDF) [file pbio.3000308.s006.pdf]

```
                                   MDSKSTIADKIEAYLGAKSDDSKIDQSLKADPSEVQYYGSGGDGYYLKNNICKITVNHSDSGTNDPCDRKPPPYGDNDQWKCDENLSKXSEKPENIXVPPRRQRMCIKNLEXLNVDKIRDKHAFLADVLL
                                            10        20        30        40        50        60        70        80        90       100       110       120       130

ALB01044.1                         MD-KSSIANKIEAYLGAKSDDSKIDQSLKADPSEVQYYGSGGDGYYLRKNICKITVNHSDSGTNDPCDRKELSYGDIDEWKCAIILSKVSEKPENVFVPPRRQRMCIKNLENLKFDKIRDKHAFLADVLL 129
ALB01053.1                         MD-KSSIANKIEAYLGAKSDDSKIDQSLKADPSEVQYYGSGGDGYYLRKNICKITVNHSDSGTNDPCDRKELSYGDIDEWKCAIILSKVSEKPENVFVPPRRQRMCIKNLENLKFDKIRDKHAFLADVLL 129
ETW56275.1                         MDSTSTIANKIEAYLGAKSDDSKIDELLKADPSEVQYYRSGGDGYYLKNNICKITVNHSDSGTNDPCDSIPPPYGDNDQWKCAIILSKVSEKPENVFVPPRRQHMCIKNLEKLNVDKIRDKHAFLADVLL 130
ALB01048.1                         MDSTSTIANKIEAYLGAKSNDSKIDQSLKADPSEVQYYGSGGDGYYLKNNICKITVNHSDSGKYDPCDRKELSYGDIDEWKCQQNSSDGSEKPENVFVPPRRQHMCIKNLENLKFDKIRDKHAFLADVLL 130
ALB01041.1                         MDSTSTIGDKIEEYLGAKSDESNIEQSLKADPSKVQYYGSGGDGYYLKNNICKITVNHSDSGKYEPCDSIPPPYGDNDQWKCDENLYKVSEKPENVFVPPRRQRMCIKNLEKLNVDKIRDKHAFLADVLL 130
ALB01039.1                         MDSTSTIGDKIEEYLGAKSDESNIEQSLKADPSKVQYYGSGGDGYYLKNNICKITVNHSDSGKYEPCDSIPPPYGDNDQWKCDENLYKVSEKPENVFVPPRRQRMCIKNLEKLNVDKIRDKHAFLADVLL 130
ALB01056.1                         MDSTSTIANKIEEYLGAKSDESNIDQSLKADPSEVQYYRSGGDGYYLKNNICKITVNHSDSGKYDPCDRKELSYGDIDQWKCAIILSKASEKPENVFVPPRRQRMCIKNLEKLNVDKIRDKHAFLADVLL 130
KNC35427.1                         MDSTSTIANKIEEYLGAKSDDSKIDELLKADPSEVEYYRSGGDGDYLKNNICKITVNHSDSGKYDPCEKKLPPYGDNDQWKCDENLYKASENNKNIFVPPRRQRMCINNLENLKFDKIRDKHAFLADVLL 130
ALB01046.1                         MDSTSTIANKIEAYLKEKSNESKIDQSLKADPSEVQYYGSGGDGYYLKNNICKITVNHSDSGTNDPCENKLLPYGDNDQWKCDENLYKASEKPENICVPPRRQRMCINNLEKLNVEKIRDKHAFLADVLL 130
ALB01037.1                         MDSTSTIANKIEEYLGAKSNESNIDQSLKADPSEVQYYRSGGDGYYLKNNICKITVNHSDSGTNDPCDSIPPPYGDNDQWKCDENLYKASEKPENVFVPPRRQRMCIKNLEKLNVDKIRDKHAFLADVLL 130
ALB01055.1                         MDSKTTIAEKIEAYLEKKSNDSKIEQSLKADPSEVQYYRSGGDGYYLKNNICKITVNHSDSGKYDPCDRKELSYGDNDQWKCQQNSSDGSGKPENICVPPRRQRMCIKNLENLKFDKIRDKHAFLADVLL 130
AAQ73924.1                         MD-KSSIGDKIEAYLEKKSDESDIEQSLKADPSEVEYYRSGGDGDYLRKNICKITVNHSDSGKYEPCDRKELSYGDIDEWKCQQNSSDGSEKPENICVPPRRQRMCIKNLENLKFDKIRDNNAFLADVLL 129
ALB01042.1                         MD-KSSIGDKIEEYLKEKSNDSKIDQSLKADPSEVQYYGSGGDGYYLKNNICKITVNHSDSGKYEPCDSIPPPYGDNDQWKCDENLSKVSEKPENICVPPRRQRMCIKNLENLKFDKIRDKHAFLADVLL 129
AJD77410.1                         MDSKTTIAEKIEEYLKEKSNDSKIDQSLKADPSEVQYYGSGGDGYYLKNNICKITVNHSDSGTNDPCEKKLLPYGDNDQWKCDENLFKASGNHKNICVPPRRQHMCINNLENLKFDKIRDKHAFLADVLL 130
EWC75615.1                         MDSTSTIGDKIEEYLEKKSDESNIDQSLKADPSKVQYYRSGGDGYYLKNNICKITVNHSDSGKYEPCDSIPPPYGDNDQWKCDENLYKASGNHKNIFVPPRRQHMCIKNLEKLNVEKIRDKHAFLADVLL 130
ALB01054.1                         MDSTSTIAEKIEAYLEKKSNEPNIDQSLKADPSKVQYYRSGGDGYYLKNNICKITVNHSDSGKYEPCQNKEP-YGTNQTWKCEITDGKEKIKNKYICVPHRRERLCTYNLENLNVDKIRDKHAFLADVLL 129
ALB01057.1                         MDSTSTIANKIEAYLEKKSNESNIEQSLKADPSEVQYYGSGGDGYYLKNNICKITVNHSDSGTNDPCDSIPPPYGDIDEWKCAIILSKVSEKPENVFVPPRRQRMCIKNLEKLNVDKIRDKHAFLADVLL 130
ALB01040.1                         MDSTSTIGDKIEEYLEKKSDESKIDQSLKADPSEVQYYGSGGDGYYLKNNICKITVNHSDSGTNDPCDSIPPPYGDNDQWKCDENLFKASGNHKNICVPPRRQHMCIKNLEKLNVDKIRDKHAFLADVLL 130
ETW41644.1                         MDSTSTIGDKIEAYLGAKSDDSKIDQSLKADPSEVQYYRSGGDGYYLKNNICKITVNHSDSGTNDPCDRIPPPYDDNDQWKCDENLSKVSGKPENICVPPRRQRMCINNLEKLNVDKIRDKHAFLADVLI 130
ALB01043.1                         MDSTSTIGDKIEEYLEKKSDESKIDQSLKADPSEVQYYGSGGDGYYLKNNICKITVNHSDSGTNDPCDSIPPPYGDNDQWKCDENLFKASGNHKNICVPPRRQHMCIKNLEKLNVDKIRDKHAFLADVLL 130
EUT82883.1                         MD-KSSIGDKIEAYLEKKSDESNIEQSLKADPSEVQYYGSGGDGYYLRKNICKITVNHSDSGTNDPCDRKELSYGDIDEWKCAIILSKVSEKPENVFVPPRRQRMCINNLENLKFDKIRDKHAFLADVLL 129
ALB01038.1                         MD-KSSIGDKIEAYLEKKSDESNIEQSLKADPSEVQYYRSGGDGDYLRKNICKITVNHSDSGTNDPCDSIPPPYGDNDQWKCQQNSSDGSGKPENICVPPRRQRMCIKNLENLNVERIRDKHAFLADVLL 129
ALB01058.1                         MD-KSSIGDKIEAYLEKKSDESNIDQSLKADPSEVQYYRSGGDGDYLRKNICKITVNHSDSGTNDPCDSIPPPYGDNDQWKCQQNSSDGSGKPENICVPPRRQRMCIKNLENLNVERIRDKHAFLADVLL 129
ALB01059.1                         MDSTSTIGDKIEEYLGAKSDESKIEQSLKADPSKVQYYGSGGDGYYLKNNICKITVNHSDSGTNDPCEKKLPPYGDNDQWKCQQNSSDGSGNHKNICVPPRRQRMCINNLENLKFDKIRDKHAFLADVLL 130
KNG76633.1                         MDSKTTIADKIEEYLGAKSDDSKIEQSLKADPSEVQYYGSGGDGYYLKNNICKITVNHSDSGTNDPCEKKLLPYGDNDQWKCDENLYKASENNKNICVPPRRQRMCINNLENLKFDKIRDKHAFLADVLL 130
ALB01051.1                         MDSTSTIGDKIEEYLGAKSDESNIEQSLKADPSEVQYYGSGGDGYYLKNNICKITVNHSDSGKYEPCDSIPPPYGDNDQWKCDENLYKVSEKPENVFVPPRRQRMCIKNLEKLNVEKIRDKHAFLADVLL 130
ALB01049.1                         MD-KSSIGDKIEEYLKEKSDDSKIDELLKADPSEVQYYRSGGDGDYLKNNICKITVNHSDSGKYDPCDRIPPPYDDNDQWKCDENLYKASENNKNIFVPPRRQRMCINNLENLKFDKIRDKHAFLADVLL 129
EWC75604.1                         MD-KSSIGDKIEAYLEKKSDESNIDQSLKADPSEVQYYRSGGDGDYLRKNICKITVNHSDSGTNDPCDSIPPPYGDNDQWKCQQNSSDGSGKPENICVPPRRQRMCIKNLENLNVEKIRDKHAFLADVLL 129
ALB01045.1                         MD-KSSIANKIEEYLKEKSDDSKIDQSLKADPSEVQYYGSGGDGYYLKNNICKITVNHSDSGTNDPCEKKLLPYGDNDQWKCDENLSKVSGKPENICVPPRRQRMCINNLEKLKFDKIRDKHAFLADVLL 129
ALB01047.1                         MDSKTTIAEKIEEYLKEKSNDSKIDQSLKADPSEVQYYGSGGDGYYLKNNICKITVNHSDSGKYEPCDSIPPPYGDNDQWKCDENLFKASGNHKNICVPPRRQHMCINNLEKLKFDKIRDKHAFLADVLL 130
7G8.pro                            MD-KSSIGDKIEEYLKEKSDDSKIDELLKADPSEVEYYRSGGDGDYLKNNICKITVNHSDSGKYEPCDRIPPPYGDNDQWKCQQNLYKASENNKNVFVPPRRQRMCINNLENLNVERIRDKHAFLADVLL 129
124_8.pro                          MDSTSTIANKIEAYLEKKSNESNIDQSLKADPSEIDYYNFGGDGYYLRKNICKITVNHSDSGTNDPCDRKELSYGDNDQWKCQQNSSDGSENNKNICVPPRRQRMCIKNLEKLNVDKIRDKHAFLADVLL 130
indo.pro                           MDSKTTIAEKIEAYLGAKSNDSKIDQSLKADPSEVQYYRSGGDGDYLKNNICKITVNHSDSGTNDPCDRKELSYGDIDEWKCDENLYKASENNKNIFVPPRRQRMCINNLEKLNVDKIRDKHAFLADVLL 130
KMWII.pro                          MD-KSSIGDKIEAYLEKKSNESNIEQSLKADPSEVEYYRSGGDGDYLRKNICKITVNHSDSGKYDPCDRIPPPYGDNDQWKCQQNSSDGSEKPENICVPPRRERLCTYNLEKLNVEKIRDKHAFLADVLL 129
M24.pro                            MDSKTTIADKIEAYLGAKSDDSKIDQSLKADPSEVQYYGSGGDGYYLKNNICKITVNHSDSGTNDPCEKKLLPYGDNDQWKCDENLYKASENNKNICVPPRRQRMCINNLENLKFDKIRDKHAFLADVLL 130
MTS1.pro                           MDSKTTIAEKIEAYLVAKSDDSKIDELLKADPSEIDYYNFGGDGDYLKNNICKITVNHSDSGKYEPCDSIPPPYGDNDQWKCQQNSSDGSGKPENVFVPPRRQRMCINNLENLKFDKIRDNNAFLADVLL 130
P13.pro                            MDSTSTIADKIEAYLKEKSNDSKIEQSLKADPSEVQYYGSGGDGYYLKNNICKITVNHSDSGTNDPCDRKLPPYGDNDQWKCAIILSKVSEKPENVFVPPRRQRMCIKNLENLNVEKIRDKHAFLADVLL 130
T2C6.pro                           MDSKTTIADKIEEYLKEKSDDSKIDQSLKADPSEVQYYRSGGDGDYLKNNICKITVNHSDSGKYDPCDRKELSYGDIDEWKCAIILSKVSEKPENVFVPPRRQRMCINNLENLNVERIRDKHAFLADVLL 130
V1_S.pro                           MDSKTTIANKIEAYLEKKSNESDIDQSLKADPSEVEYYRSGGDGDYLRKNICKITVNHSDSGMYEPCDRKELSYGDIDEWKCDENLFKASGNHKNICVPPRRQHMCIKNLEKLNVEKIRDNNAFLADVLL 130
WR80.pro                           MDSKTTIAEKIEEYLKEKSNESNIEQSLKADPSEVQYYGSGGDGYYLKNNICKITVNHSDSGTNDPCDRIPPPYDDNDQWKCAIILSKVSEKPENVFVPPRRQRMCINNLEKLKFDKIRDKHAFLADVLL 130
PFL0030cexon1+2.pro                MD-KSSIANKIEAYLGAKSDDSKIDQSLKADPSEVQYYGSGGDGYYLRKNICKITVNHSDSGTNDPCDRIPPPYGDNDQWKCAIILSKVSEKPENVFVPPRRQRMCINNLEKLNVDKIRDKHAFLADVLL 129
A4PFL0030c exon1+2.pro             MDSTSTIANKIEEYLGAKSDDSKIDELLKADPSEVEYYRSGGDGDYLKNNICKITVNHSDSGKYDPCEKKLPPYDDNDQWKCQQNSSDGSGKPENICVPPRRERLCTYNLENLKFDKIRDNNAFLADVLL 130
Newdd2var2CSAsequence.pro          MD-KSSIGDKIEAYLEKKSDESDIEQSLKADPSEVEYYRSGGDGDYLRKNICKITVNHSDSGKYEPCDRKELSYGDIDEWKCQQNSSDGSEKPENICVPPRRQRMCIKNLENLKFDKIRDNNAFLADVLL 129
McPFL0030c.pro                     MDSTSTIANKIEAYLGAKSDDSKIDELLKADPSEVQYYRSGGDGYYLKNNICKITVNHSDSGTNDPCDSIPPPYGDNDQWKCAIILSKVSEKPENVFVPPRRQHMCIKNLEKLNVDKIRDKHAFLADVLL 130
HB3-1var2CSA.pro                   MDSTSTIGDKIEAYLGAKSNDSKIDQSLKADPSKVQYYGSGGDGYYLKNNICKITVNHSDSGTNDPCEKKELSYGDNDQWKCQQNSSDGSENNKNIFVPPRRQRMCIKNLEKLNVDKIRDKHAFLADVLL 130
HB3-2var2CSA.pro                   MDSKTTIAEKIEEYLKEKSNDSKIDQSLKADPSEIDYYNFGGDGYYLKNNICKITVNHSDSGTNDPCEKKLLPYGDNDQWKCDENLFKASGNHKNICVPPRRQHMCINNLEKLKFDKIRDKHAFLADVLL 130
```

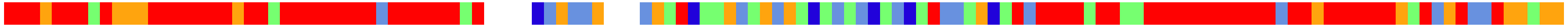

```
                              TARNEGERIVQNHPDTNSSNVCVALERSFADIADIIRGTD----QWKGTN---SNLEQNLKQMFAKILEKDKTLKDKYPNDDQNYTKLREDWWNANRQKVWEVITCGARSNDLLIKRRWTTSKESNGENK
                                        140       150       160       170       180       190       200       210       220       230       240       250       260

ALB01044.1                    TARNEGEKIVQNHPDTNSSNVCVALERSFADLADIIRGTD----QWKGTN---SNLEQNLKQMFAKIREKDSTLKNNYSKKDQNYTKLREDWWTKNRQKVWEVITCGARSNDLLIKRRWTTSGKSNGENK 252
ALB01053.1                    TARNEGEKIVQNHPDTNSSNVCVALERSFADLADIIRGTD----QWKGTN---SNLEQNLKQMFAKIREKDSTLKNNYSKKDQNYTKLREDWWTKNRQKVWEVITCGARSNDLLIKRRWTTSGKSNGENK 252
ETW56275.1                    TARKEGERIVQNHPDTNSSNVCNALERSFADIADIIRGTD----LWKGTN---SNLEKNLKQMFKNILEKGSTIQSNYSKD-QNYRKLREDWWNANRQKVWEVITCGARSNDLLIKRGWTTSGKSNGDNK 252
ALB01048.1                    TARNEGERIVQNHPDTNSSNVCVALERSFADIADIIRGTD----QWKGTN---SNLEKNLKQMFAKIREKDSTLKNNYSK-DQNYTKLREDWWTKNRQKVWEVITCGARSNDLLIKRRWRTSGKSNGENK 252
ALB01041.1                    TARNEGERIVQNHPDTNSSNVCNALERSFADIADIIRGRDENKCETKSPN----NVEELIKNFLGKNYRSNEEYRKKYQNDDENYKKLREAWWNANRQKVWEVITCSARSNDLLIKRRWRTSKESNGENK 256
ALB01039.1                    TARNEGERIVQNHPDTNSSNVCNALERSFADIADIIRGRDENKCETKSPN----NVEELIKNFFGKNYRSNEEYRKKYQNDDENYKKLREAWWNANRQKVWEVITCSARSNDLLIKRRWRTSKESNGENK 256
ALB01056.1                    TARNEGERIVQNHPDTNSSNVCVALERSFADIADIIRGRDENKCETKSPN----NVEELIKNFFGKNYRENKEYKRKYPNDDPKYTKLREAWWNANRQKVWEVITCSARSNDLLIKRGWRTSKESNGENK 256
KNC35427.1                    TARNEGERIVQNHPDTNSSNVCVALERSFADIADIIRGRDGNKCETKSPN----NVEELIKKFFEKNYRSNEEYKRKYQNDDENYKKLREDWWTKNRQKVWEVITCGARSNDLLIKRRWTTSKESNGDNK 256
ALB01046.1                    TARNEGEKIVQNHPDTNSSNVCVALERSFADLADIIRGTD----LWKGTN---SNLEKNLKQMFKKILEKDSTLKNNYSNDDENYTKLREAWWNANRQKVWEVITCSARSNDLLIKRRWRTSKESNGENK 253
ALB01037.1                    TARNEGERIVQNHPDTNSSNVCVALERSFADIADIIRGRDENKCETKSPN----NVEELIKNFFGKNYRENKEYKRKYRNDDPKYTKLREAWWNANRQKVWEVITCGARSNDLLIKRGWRTSGKSNGDNK 256
ALB01055.1                    TARNEGERIILYHPDTNSSNVCNALERSFADIADIIRGTD----LWKGTN---SNLEKNLKQMFAEILEKDSTLKNNYSKKDQNYRKLREDWWNANRQKVWEVITCSARSNDLLIKRRWTTSGKSNGENK 253
AAQ73924.1                    TARNEGERIVQNHPDTNSSNVCVALERSFADLADIIRGTDQWKGTN--------KFEPCLKQMFAKIRENDKVLQDKYPNDDPKYTKLREDWWNANRQKVWEVITCGARSNDLLIKLGWTSSKESEGDNK 251
ALB01042.1                    TARNEGERIILYHPDTNSSNVCNALERSFADIADIIRGTD----QWKGTN---SNLEQNLKQMFAKIRENDKVLQDKYPNDDENYKKLREDWWNANRQKVWEVITCGARSNDLLIKRGWTTSKESNGENK 252
AJD77410.1                    TARNEGERIVQNHPDTNSSNVCVALERSFADLADIIRGRDENKCETKSTN----NVEELIKNFFGKNYRSNEEYKRKYRNDDPKYTKLREAWWNANRQKVWEVITCGARSNDLLIKRRWTTSKESNGENK 256
EWC75615.1                    TARNEGERIVQNHPDTNSSNVCVALERSFADLADIIRGTD----QLKGSN----ILEQNLRRMFKKILEKDKVLQDKYQNDDENYKKLREDWWNANRQKVWEVITCSARSNDLLIKRRWTTSGKSNGENK 252
ALB01054.1                    TARNEGEKIVQNHPDTNSSNVCVALERSFADLADIIRGTD----LWKGTN---SNLEKNLKQMFKNILEKDSTLKNNYSNDDENYKKLREAWWNANRQKVWEVITCSARSNDLLIKRRWTTSGKSNGENK 252
ALB01057.1                    TARNEGEKIVQNHPDTNSSNVCNALERSFADIADIIRGTDQWKGTN---S----NLEKNLKQMFARILEKDSTLKNNYSKDQ-NYRKLREDWWNANRQKVWEVITCSARSNDLLIKRGWRTSGKSDRKKN 252
ALB01040.1                    TARNEGERIVQNHPDTNSSNVCNALERSFADIADIIRGTD----LWKGTN---SNLEQNLRRMFAKIRENDKVLQDKYPK-DQNYTKLREDWWNANRQKVWEVITCGARSNDLLIKRGWTTSGKSNGENK 252
ETW41644.1                    TARNEGERIILYHPDTNSSNVCVALERSFADLADIIRGTDLWKGTN---S----NLEQNLKQMFAKILEKDKVLQDKYPKD-QNYTKLREDWWTKNRQKVWEVITCGARSNDLLIKRRWTTSKESNGDNK 252
ALB01043.1                    TARNEGERIVQNHPDTNSSNVCNALERSFADIADIIRGTD----LWKGTN---SNLEQNLRRMFAKIRENDKVLQDKYPK-DQNYTKLREDWWNANRQKVWEVITCGARSNDLLIKRGWTTSGKSNGENK 252
EUT82883.1                    TARNEGEKIVQNHPDTNSSNVCVALERSFADLADIIRGTD----QWKGTN---SNLEQNLKQMFAKILEKDSTLKNNYSKKDQNYTKLRESWWTKNRQKVWEVITCGARSNDLLIKRRWTTSGKSNGENK 252
ALB01038.1                    TARNEGERIVQNHPDTNSSNVCVALERSFADIADIIRGRDGNKCETKSPN----NVEELIKKFFGKNYRENKEYKRKYPNDDPKYTKLREDWWTKNRQKVWEVITCSARSNDLLIKRGWRTSGKSNGENK 255
ALB01058.1                    TARNEGERIVQNHPDTNSSNVCVALERSFADIADIIRGRDGNKCETKSPN----NVEELIKKFFRKNYRENKEYKRKYPNDDPKYTKLREDWWTKNRQKVWEVITCSARSNDLLIKRGWRTSGKSNGENK 255
ALB01059.1                    TARNEGERIVQNHPDTNSSNVCVALERSFADIADIIRGTD----QLKGSN----ILEQNLKQMFKKILEKDSTLKNNYSNDDENYKKLREAWWNANRQKVWEVITCSARSNDLLIKRRWTTSKESNGENK 252
KNG76633.1                    TARNEGEKIVQNHPDTNSSNVCVALERSFADIADIIRGTD----QWKGTN---SNLEQNLKQMFAKILEKDSTLKNNYSKKDQNYTKLREDWWTKNRQKVWEVITCGARSNDLLIKRRWRTSKESNGENK 253
ALB01051.1                    TARNEGERIILYHPDTNSSNVCVALERSFADLADIIRGRD----GNKCETKSTNNVEELIKKFFEKNYRSNEEYKRKYPNDDQNYRKLREAWWTKNRQKVWEVITCSARSNDLLIKRRWTTSGKSNGENK 256
ALB01049.1                    TARNEGERIILYHPDTNSSNVCIALERSFADLADIIRGRD----GNKCETKSTNNVEELIKKFFEKNYRSNEEYKRKYPNDDPKYTKLREDWWTKNRQKVWEVITCSARSNDLLIKRRWTTSGKSNGDNK 255
EWC75604.1                    TARNEGEKIVQNHPDTNSSNVCVALERSFADIADIIRGTD----LWKGTN---SNLEQNLKQMFKNILEKGSTIQSNYSKDQ-NYRKLREAWWNANRQKVWEVITCGARSNDLLIKRRWTTSKESNGDNK 251
ALB01045.1                    TARNEGEKIVQNHPDTNSSNVCVALERSFADLADIIRGTD----QWKGTN---SNLEQNLRRMFAKILEKDSTLKNNYSKKDQNYTKLREAWWTKNRQKVWEVITCSARSNDLLIKRRWRTSKESNGENK 252
ALB01047.1                    TARNEGERIVQNHPDTNSSNVCVALERSFADLADIIRGRD----ENKCETKSTNNVEELIKNFFGKNYRSNEEYKRKYRNDDPKYTKLREAWWNANRQKVWEVITCGARSNDLLIKRRWRTSKESNGDNK 256
7G8.pro                       TARNEGERIVQNHPDTNSSNVCIALERSFADIADIIRGRDGNKCETKSTN----NVEELIKKFFEKNYRENKEYKRKYPNDDPKYTKLREDWWTKNRQKVWEVITCGARSNDLLIKRGWTTSKESEGENK 255
124_8.pro                     TARNEGEKIVQNHPDTNSSNVCVALERSFADIADIIRGTD----LWKGTN---SNLEKNLKQMFAKILEKDKVLQDKYPNDDENYKKLREDWWNANRQKVWEVITCSARSNDLLIKRRWRTSKESNGENK 253
indo.pro                      TARNEGERIILYHPDTNSSNVCVALERSFADLADIIRGTD----QWKGTN---SNLEKNLKQMFAKIRENDKVLQDKYPNDDENYKKLREDWWTKNRQKVWEVITCSARSNDLLIKRRWRTSKESEGENK 253
KMWII.pro                     TARNEGERIVQNHPDTNSSNVCVALERSFADLADIIRGTD----LWKGTN----KFEPYLKQMFAKILEKDSTLKNNYSKKDPKYTKLREDWWTKNRQKVWEVITCSARSNDLLIKRRWTTSGKSNGENK 251
M24.pro                       TARNEGEKIVQNHPDTNSSNVCVALERSFADIADIIRGTD----QWKGTN---SNLEQNLKQMFAKIREKDSTLKNNYSK-DQNYRKLREDWWTKNRQKVWEVITCGARSNDLLIKRRWRTSKESNGENK 252
MTS1.pro                      TARNEGERIVQNHPDTNSSNVCIALERSFADLADIIRGRDGNKCETKSPN----NVEELIKKFFEKNYRSNEEYKRKYPNDDENYKKLREDWWTKNRQKVWEVITCGARSNDLLIKRGWTTSGKSNGENK 256
P13.pro                       TARNEGERIILYHPDTNSSNVCVALERSFADLADIIRGTDQWKGTN---S----NLEQNLKQMFKNILEKDKVLQDKYQNDDENYKKLRESWWTKNRQKVWEVITCSARSNDLLIKRGWRTSKESEGENK 253
T2C6.pro                      TARNEGERIVQNHPDTNSSNVCVALERSFADLADIIRGTD----QWKGTN---SNLEKNLKQMFAKIRENDKVLQDKYPNDDENYKKLREDWWTKNRQKVWEVITCGARNNDLLIKRRWRTSKESNGENK 253
V1_S.pro                      TARNEGERIILYHPDTNSSDVCNALERSFADLADIIRGRDENKCETKSPN----NVEELIKNFFGKNYRENKEYRKKYQNDDENYKKLREAWWNANRQKVWEVITCSARSNDLLIKRRWRTSKESNGDNK 256
WR80.pro                      TARNEGERIVQNHPDTNSSNVCVALERSFADIADIIRGTD----LWKGTN---SNLEQNLKQMFAKILEKDKVLQDKYPKDQ-NYTKLREAWWNANRQKVWEVITCSARSNDLLIKRRWTTSGKSNGDNK 252
PFL0030cexon1+2.pro           TARNEGERIVQNHPDTNSSNVCNALERSFADIADIIRGTD----LWKGTN---SNLEQNLKQMFAKIRENDKVLQDKYPK-DQNYRKLREDWWNANRQKVWEVITCGARSNDLLIKRGWRTSGKSNGDNK 251
A4PFL0030c exon1+2.pro        TARNEGEKIVQNHPDTNSSNVCNALERSFADLADIIRGTD----QWKGTN---SNLEKNLKQMFAKIRENDKVLQDKYPK-DQKYTKLREAWWNANRQKVWEVITCGARSNDLLIKRGWRTSGKSDRKKN 252
Newdd2var2CSAsequence.pro     TARNEGERIVQNHPDTNSSNVCVALERSFADLADIIRGTDQWKGTN--------KFEPCLKQMFAKIRENDKVLQDKYPNDDPKYTKLREDWWNANRQKVWEVITCGARSNDLLIKLGWTSSKESEGDNK 251
McPFL0030c.pro                TARKEGERIVQNHPDTNSSNVCNALERSFADIADIIRGTD----LWKGTN---SNLEKNLKQMFKNILEKGSTIQSNYSKD-QNYRKLREDWWNANRQKVWEVITCGARSNDLLIKRGWTTSGKSNGDNK 252
HB3-1var2CSA.pro              TARNEGERIILYHPDTNSSNVCVALERSFADIADIIRGTD----QLKGSN----ILEQNLKQMFAKIREKDKVLQDKYPNDDENYKKLREDWWNANRQKVWEVITCSARSNDLLIKRRWTTSKESEGENK 252
HB3-2var2CSA.pro              TARNEGERIVQNHPDTNSSNVCVALERSFADIADIIRGRDENKCETKSTN----NVEELIKNFFGKNYRSNEEYKRKYRNDDPKYTKLREAWWNANRQKVWEVITCGARSNDLLIKRRWRTSKESNGDNK 256
```

```
                            LELCRKCGHYEEKVPTKLDYVPQFLRWLTEWIEDXYREKQNLIDDMERHREECTREDDKSKEGTSYCNMCKDKCKKYCECVKKWKSEWENQKNK-YKDLYQQ-KNETSSSSKNTSRYDDYVKEFFXKLKG
                                      270       280       290       300       310       320       330       340       350       360       370       380       390

ALB01044.1                  LELCRKCGHYEEKVPTKLDYVPQFLRWLTEWIEDFYREKQNLIDGMERHREECTREDDKCKEGTSYCNMCKEKHTKYCECVKKWKTEWENQKNK-YKDLYQQ-ENETSQ--KNTSRYDDYVKEFFKNFKG 378
ALB01053.1                  LELCRKCGHYEEKVPTKLDYVPQFLRWLTEWIEDFYREKQNLIDGMERHREECTREDDKCKEGTSYCNMCKEKHTKYCECVKKWKTEWENQKNK-YKDLYQQ-ENETSQ--KNTSRYDDYVKEFFKNFKG 378
ETW56275.1                  LELCRKCGHYEKEVPTKLDYVPQFLRWLTEWIEDLYREKQNLIDDMERHREECTRKNVDSKEGTSYCSTCKDKCKKYCECVKKWKTEWENQENK-YKDLYKKDKRKKSS-AKHASRYDDYVKDFFEKLNE 380
ALB01048.1                  LELCRKCGHYEKEVPTKLDYVPQFLRWLTEWIEDFYREKQNLIDDMERHREECTSEDHKCKEGTSYCSTCKDKCKKYCECVKKWKTEWENQKNK-YTELYEQ-ENETSSSSKKKSRYDDYVKDFFKKLNE 380
ALB01041.1                  LELFRKCGHYEEKVPTKLDYVPQFLRWLTEWIEDLYREKQNLIDDMERHREECTREDDKCKEVTSYCNMCKDKCKKYCECVKKWKSEWENQKNK-YKDLYQQNKNETSSSSKNASRYDDYVKDFFKKLEA 385
ALB01039.1                  LELFRKCGHYEEKVPTKLDYVPQFLRWLTEWIEDLYREKQNLIDDMERHREECTREDDKCKEVTSYCNMCKDKCKKYCECVKKWKSEWENQKNK-YKDLYQQNKNETSSSSKNASRYDDYVKDFFKKLEA 385
ALB01056.1                  LELCRKCGHYEEKVPTKLDYVPQFLRWLTEWIEDLYREEQNLIDDMERHREECTREDDKCKEVTSYCNMCKEKHTKYCEFVNKWKTEWENQKNK-YKDLYQQ-ENETSSSPKKKSRYDDYVKEFFEKLKG 384
KNC35427.1                  LELCRKCGHYEGKVPTYLDYVPQFLRWLTEWIEDFYREKQNLIDDMERHREECTRKNVESKEGTSYCSTCKDKCKKYCECVKKWKTEWENQKNK-YNDLYQQE-NETSSSSKKKSRYDDYVKEFFKNFKG 384
ALB01046.1                  LELCRKCGHYEKEVPTKLDYVPQFLRWLTEWIEDFYREKQNLIDDMERHREECTSEDHKCKEVTSYCTNCKEKCKKYCECVKKWKSEWKNQENK-YKELYEQ-ENKTSSSSKKKSRYDDYVKDFFKKL-E 380
ALB01037.1                  LELCRKCGHYEGKVPTKLDYVPQFLRWLTEWIEDLYREKQNLIDDMERHREECTSEDHKSKEVTSYCSTCKDKCKKYCECVKKWKTEWENQKNK-YTELYEQ-ENETS--QKNTSRYDNYVKEFFKNFKG 382
ALB01055.1                  LELCRKCGHYEEKVPTYLDYVPQFLRWLTEWIEDFYREKQNLIDDMERHREECTREDHKSKEVTSYCNMCKEKHTKYCECVKKWKSEWKNQENK-YKDLYEQNKNKTSSSSKNTSRYDDYVKEFFEKL-E 381
AAQ73924.1                  LELCRKCGHYEEKVPTKLDYVPQFLRWLTEWIEDFYREKQNLIDGMERHREECTRKNVESKEGTSYCTNCKEKCKKYCEYVNKWKSEWENQKNK-YNDLYQQE-NKTSSSSKKKSRYDDYVKEFFKNFKG 379
ALB01042.1                  LELCRKCGHYEEKVPTQLDYVPQFLRWLTEWIEDFYREKQNLIDDMERHREECTREYDKCKEGASYCNMCKDKCKKYCECVKKWKSEWENQKNK-YKDLYQQNKNKTSSSSKNTSRYDNYVKEFFEKLNE 381
AJD77410.1                  LELCRKCGHYEEKVPTKLDYVPQFLRWMDEWVEGFCRKRQNIIDRLEYYCEKCNQGKDNCVNSTTENSACKDKCKKYCECVKKWKSEWENQKNK-YTELYEQNKNKTSSSPKKKSRYDDYVKEFFEKLNE 385
EWC75615.1                  LELFRKCGHYEEKVPTKLDYVPQFLRWLTEWIEDLYREEQNLIDDMERHREECTREYDKSKEGTSYCSTCKEKCKKYCEFVNKWKSEWENLKNK-YKELYEQ-ENKTSSSSKNTSRYDDYVKEFFKKLKG 380
ALB01054.1                  LELCRKCGHYEEKVPTKLDYVPQFLRWLTEWIEDLYREEQNLIDDMERHREECTSEDHKCKEVTSYCSTCKEKHTKYCEYVNKWKSEWKNQKNK-YKELYEQNKNKTSSSAKHASRYDDYVKDFFEKLNE 381
ALB01057.1                  FELCRKCGHYEGKVPTKLDYVPQFLRWLTEWIEDLYREKQNLIDDMERHREECTRKYVESKEVTSYCNMCKDKCKKYCECVKKWKSEWENQENK-YKDLYQQNKNKTSSSSKNTSRYDDYVKEFFEKLNE 381
ALB01040.1                  LELCRKCGHYEKEVPTKLDYVPQFLRWLTEWIEDLYRGKQNLIDDMERHREECTRKNVESKEGTSYCNMCKEKHTKYCECVKKWKSEWENQKNK-YKDLYQQ---ENETSQKNTSRYDNYVKEFFKNFKG 378
ETW41644.1                  LELCRKCGHYEKEVPTKLDYVPQFLRWLTEWIADLYREKQNLIDDMERHREECTSEDDKCKEGTSYCNMCKEKHTKYCECVKKWKTEWENQENK-YKDLYQQE-NETSSSSKNTSRYDDYVKEFFKNFKG 380
ALB01043.1                  LELCRKCGHYEKEVPTKLDYVPQFLRWLTEWIEDLYRGKQNLIDDMERHREECTRKNVESKEGTSYCNMCKEKHTKYCECVKKWKSEWENQKNK-YKDLYQQ---ENETSQKNTSRYDNYVKEFFKNFKG 378
EUT82883.1                  LELCRKCGHYEEKVPTKLDYVPQFLRWLTEWIEDLYREKQNLIDGMERHREECTREDDKSKEVTSYCNMCKDKCKKYCECVKKWKTEWENQKNK-YKDLYQQNKNKTSSSSKNTSRYDDYVKEFFEKLNE 381
ALB01038.1                  LELCRKCGHYEKEVPTKLDYVPQFLRWLTEWIEDLYREKQNLIDDMERHREECTSEDHKSKEVTSYCSTCKDKCKKYCECVKKWKSEWENQKNK-YKDLYEQ-ENETSSSSKKKSRYDDYVKDFFENFKG 383
ALB01058.1                  LELCRKCGHYEKEVPTKLDYVPQFLRWLTEWIEDLYREKQNLIDDMERHREECTSEDHKSKEVTSYCSTCKDKCKKYCECVKKWKSEWENQKNK-YKDLYEQ-ENETSSSSKKKSRYDDYVKDFFENFKG 383
ALB01059.1                  LELCRKCGHYEEKVPTKLDYVPQFLRWLTEWIEDLYREEQNLIDGMERHREECTSQDDKSNEGTSYCTNCKDKCKKYCECVKKWKTEWENQKNK-YNDLYQQKKNETSSSSKKKSRYDDYVKEFFEKLEV 381
KNG76633.1                  LELFRKCGHYEKEVPTKLDYVPQFLRWLTEWIEDFYREKQNLIDGMERHREECTSEDHKSKEGTSYCNMCKDKCKKYCECVKKWKSEWENQENK-YKDLYEQNKNKTSSSSKNTSRYDDYVKEFFEKLKG 382
ALB01051.1                  LELCRKCGHYEEKVPTKLDYVPQFLRWLTEWIEDLYREKQNLIDDMERHREECTRKYVESNEGTSYCNMCKEKHTKYCECVKKWKSEWENQKNK-YKDLYQQENETSSSSKNT-SRYDNYVKEFFKNFKG 384
ALB01049.1                  LELFRKCGHYEKEVPTKLDYVPQFLRWVDEWVEGFCRKRQNIIDRLEENCKKYKEGNNNCVNGTTENSACKDQCVKYCTFIRNRKNHWEEQKKKKYKSLYQDAVGSSDSSLYVPSSVDDNVKEFFEKLKG 385
EWC75604.1                  LELCRKCGHYEKEVPTKLDYVPQFLRWMDEWVEGFCRKRQNIIDRLEYYCEKCNQGKDNCVNGTTENSACKDQYVKYCTFIRNRKNHWEEKKEK-YDYLYKSANESSDSSLYVPSSVDDNVKEFFEKLKG 380
ALB01045.1                  LELCRKCGHYEKEVPTKLDYVPQFLRWLTEWIEDFYREKQNLIDDMERHREECTREDDKCKEGTSYCNMCKDKCKKYCECVKKWKTEWENQKNK-YTELYEQNKNKTS--QKNTSRYDDNVKEFFEKLKG 379
ALB01047.1                  LELCRKCGHYEEKVPTKLDYVPQFLRWMDEWVEGFCRKRQNIIDRLEYYCEKCNQGKDNCVNSTKENSACKVQCGKYLTFIKNKNKEWEQQKEK-YDYLYKSANESSDSSLYINSSVDDNVTDFLKQLKG 385
7G8.pro                     LELCRKCGHYEEKVPTKLDYVPQFLRWLTEWIEDLYREKQNLIDDMERHREECTSEDHKSKEGASYCNMCKDKCKKYCECVKKWKSEWENQKNK-YKDLYQQ-ENETSSSSKKKSRYDDYVKEFFKKLNE 383
124_8.pro                   LELCRKCGHYEGKVPTKLDYVPQFLRWLTEWIEDLYREKQNLIDDMERHREECTREYDKCKEVTSYCNMCKEKCKKYCECVKKWKTEWENQENK-YNDLYKKD-KRKKSSAKHASRYDNYVKEFFEKLKG 381
indo.pro                    LELCRKCGHYEGKVPTYLDYVPQFLRWLTEWIEDFYREKQNLIDDMERHREECTSEDHKSKEVTSYCSTCKEKHTKYCECVKKWKSEWENQKNK-YTELYEQ-ENETSSSSKNTSRYDDYVKEFFKNFKG 381
KMWII.pro                   LELCRKCGHYEGKVPTKLDYVPQFLRWLTEWIADLYREKQNLIDDMERHREECTREDHKSKEGASYCNMCKEKHTKYCECVKKWKTEWENQKNK-YNDLYQQE-NETSSSSKKKSRYDDYVKDFFENFKG 379
M24.pro                     LELFRKCGHYEKEVPTKLDYVPQFLRWLTEWIEDFYREKQNLIDGMERHREECTSEDDKSKEVTSYCNMCKDKCKKYCECVKKWKSEWENQKNK-YKDLYEQNKNKTSSSSKKKSRYDDYVKEFFKNFKG 381
MTS1.pro                    LELCRKCGHYEGKVPTKLDYVPQFLRWLTEWIEDFYREKQNLIDDMERHREECTSEDDKSKEGASYCNMCKDKCKKYCECVKKWKSEWENQKNK-YKDLYQQ-ENETSSSSKKKSRYDNYVKDFFENFKG 384
P13.pro                     LELCRKCGHYEGKVPTKLDYVPQFLRWLTEWIEDFYREKQNLIDDMERHREECTRKYVECKEGTSYCNMCKEKHTKYCECVKKWKTEWKNQKNK-YKDLYEQNKNETSSSPKKKSRYDDYVKEFFKKLNE 382
T2C6.pro                    LELFRKCGHYEKEVPTKLDYVPQFLRWLTEWIEDLYREKQNLIDDMERHREECTREDDKCKEGTSYCSTCKDKCKKYCECVKKWKSEWENQKNK-YKDLYEQNKNKTSSSSKKKSRYDNYVKEFFEKLNE 382
V1_S.pro                    LELFRKCGHYEGKVPTKLDYVPQFLRWLTEWIVDFYREKQNLIDDMERHREECTREDHKSKEGTSYCSTCKEKHTKYCEFVKKWKSEWENQKNK-YTELYEQ--NKNKTSQKNTSRYDDYVKEFFKNFKG 383
WR80.pro                    LELCRKCGHYEKEVPTKLDYVPQFLRWMDEWVEGFCRKRQSIFDRLEYYCEKCNQGKDNCVNSTTENSACKVQCGKYCTFIKNKNKEWEQQKEK-YDYLYKSANESSDSSLYINSSVDDNVTDFLKQLKG 381
PFL0030cexon1+2.pro         LELCRKCGHYEEKVPTKLDYVPQFLRWLTEWIEDFYREKQNLIDDMERHREECTSEDHKSKEGTSYCSTCKDKCKKYCECVKKWKSEWENQKNK-YTELYQQNKNETSQ--KNTSRYDDYVKDFFKKLEA 378
A4PFL0030c exon1+2.pro      FELCRKCGHYEKEVPTKLDYVPQFLRWLTEWIEDFYREKQNLIDDMERHREECTREDHKSKEGTSYCSTCKDKCKKYCECVKKWKTEWENQENK-YKDLYEQ--NKNKTSQKNTSRYDDYVKDFFEKLEA 379
Newdd2var2CSAsequence.pro   LELCRKCGHYEEKVPTKLDYVPQFLRWLTEWIEDFYREKQNLIDGMERHREECTRKNVESKEGTSYCTNCKEKCKKYCEYVNKWKSEWENQKNK-YNDLYQQE-NKTSSSSKKKSRYDDYVKEFFKNFKG 379
McPFL0030c.pro              LELCRKCGHYEKEVPTKLDYVPQFLRWLTEWIEDLYREKQNLIDDMERHREECTRKNVDSKEGTSYCSTCKDKCKKYCECVKKWKTEWENQENK-YKDLYKKDKRKKSS-AKHASRYDDYVKDFFEKLNE 380
HB3-1var2CSA.pro            LELCRKCGHYEEKVPTKLDYVPQFLRWLTEWIEDLYREEQNLIDDMERHREECTRKNVESNEGASYCNMCKEKHTKYCECVKKWKSEWENQKNK-YTELYEQ---ENETSQKNTSRYDDYVKDFFEKLKG 378
HB3-2var2CSA.pro            LELCRKCGHYEEKVPTKLDYVPQFLRWLTEWIEDLYREEQNLIDDMERHREECTRKNVESNEGASYCNMCKEKHTKYCECVKKWKSEWENQKNK-YTELYEQ---ENETSQKNTSRYDDYVKDFFEKLKG 382
```

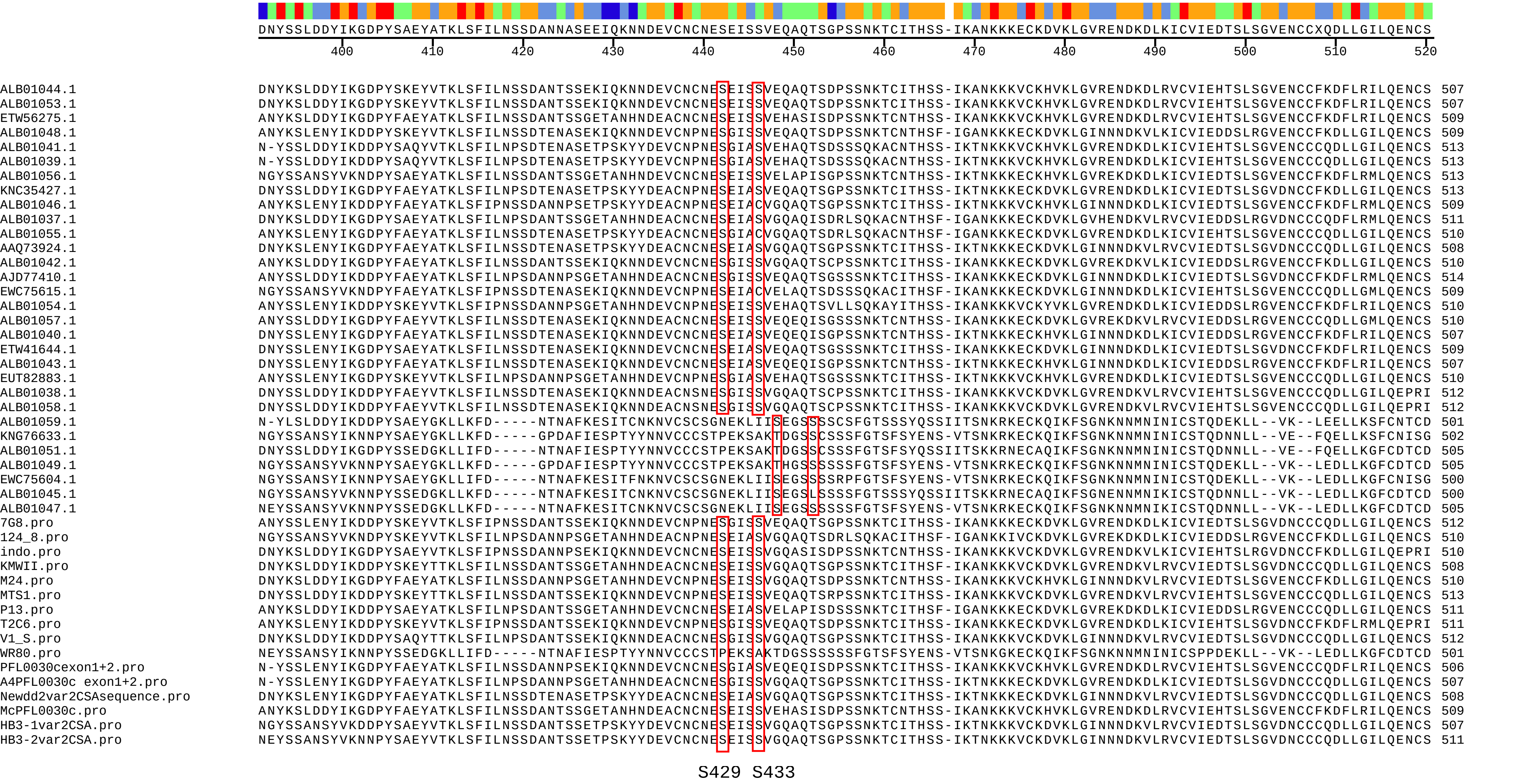
DNYSSLDDYIKGDPYSAEYATKLSFILNSSDANNASEEIQKNNDEVCNCNESEISSVEQAQTSGPSSNKTCITHSS-IKANKKKECKDVKLGVRENDKDLKICVIEDTSLSGVENCCXQDLLGILQENCS
400 410 420 430 440 450 460 470 480 490 500 510 520
ALB01044.1 DNYKSLDDYIKGDPYSKEYVTKLSFILNSSDANTSSEKIQKNNDEVCNCNESEISSVEQAQTSDPSSNKTCITHSS-IKANKKKVCKHVKLGVRENDKDLRVCVIEHTSLSGVENCCFKDFLRILQENCS 507
ALB01053.1 DNYKSLDDYIKGDPYSKEYVTKLSFILNSSDANTSSEKIQKNNDEVCNCNESEISSVEQAQTSDPSSNKTCITHSS-IKANKKKVCKHVKLGVRENDKDLRVCVIEHTSLSGVENCCFKDFLRILQENCS 507
ETW56275.1 ANYKSLDDYIKGDPYFAEYATKLSFILNSSDANTSSGETANHNDEACNCNESEISSVEHASISDPSSNKTCNTHSS-IKANKKKVCKHVKLGVRENDKDLRVCVIEHTSLSGVENCCFKDFLRILQENCS 509
ALB01048.1 ANYKSLENYIKDDPYSKEYVTKLSFILNSSDTENASEKIQKNNDEVCNPNESGISSVEQAQTSDPSSNKTCNTHSF-IGANKKKECKDVKLGINNNDKVLKICVIEDDSLRGVENCCCKDLLGILQENCS 509
ALB01041.1 N-YSSLDDYIKDDPYSAQYVTKLSFILNPSDTENASETPSKYYDEVCNPNESGIASVEHAQTSDSSSQKACNTHSS-IKTNKKKVCKHVKLGVRENDKDLKICVIEHTSLSGVENCCCQDLLGILQENCS 513
ALB01039.1 N-YSSLDDYIKDDPYSAQYVTKLSFILNPSDTENASETPSKYYDEVCNPNESGIASVEHAQTSDSSSQKACNTHSS-IKTNKKKVCKHVKLGVRENDKDLKICVIEHTSLSGVENCCCQDLLGILQENCS 513
ALB01056.1 NGYSSANSYVKNDPYSAEYATKLSFILNSSDANTSSGETANHNDEVCNCNESEISSVELAPISGPSSNKTCNTHSS-IKTNKKKECKHVKLGVREKDKDLKICVIEDTSLSGVENCCFKDFLRMLQENCS 513
KNC35427.1 DNYSSLDDYIKGDPYFAEYATKLSFILNPSDTENASETPSKYYDEACNPNESEIASVEQAQTSGPSSNKTCITHSS-IKTNKKKECKDVKLGVRENDKDLKICVIEDTSLSGVDNCCFKDLLGILQENCS 513
ALB01046.1 ANYKSLENYIKDDPYFAEYATKLSFIPNSSDANNPSETPSKYYDEACNPNESEIACVGQAQTSGPSSNKTCITHSS-IKTNKKKVCKHVKLGINNNDKDLKICVIEDTSLSGVENCCFKDFLRMLQENCS 509
ALB01037.1 DNYKSLDDYIKGDPYSAEYVTKLSFILNPSDANTSSGETANHNDEACNCNESEIASVGQAQISDRLSQKACNTHSF-IGANKKKECKDVKLGVHENDKVLRVCVIEDDSLRGVDNCCCQDFLRMLQENCS 511
ALB01055.1 ANYKSLENYIKGDPYFAEYATKLSFILNSSDTENASETPSKYYDEACNCNESGIACVGQAQTSDRLSQKACNTHSF-IGANKKKECKDVKLGVRENDKDLKICVIEHTSLSGVENCCCQDLLGILQENCS 510
AAQ73924.1 DNYKSLENYIKGDPYFAEYATKLSFILNSSDTENASETPSKYYDEACNCNESEIASVGQAQTSGPSSNKTCITHSS-IKTNKKKECKDVKLGINNNDKVLKICVIEDTSLSGVDNCCCQDLLGILQENCS 508
ALB01042.1 ANYKSLDDYIKGDPYFAEYATKLSFILNSSDANTSSEKIQKNNDEVCNCNESGISSVGQAQTSCPSSNKTCITHSS-IKANKKKECKDVKLGVREKDKVLKICVIEDDSLRGVENCCFKDLLGILQENCS 510
AJD77410.1 ANYSSLDDYIKDDPYFAEYATKLSFILNPSDANNPSGETANHNDEACNCNESGISSVEQAQTSGSSSNKTCITHSS-IKANKKKECKDVKLGINNNDKDLKICVIEDTSLSGVDNCCFKDFLRMLQENCS 514
EWC75615.1 NGYSSANSYVKNDPYFAEYATKLSFIPNSSDTENASEKIQKNNDEVCNPNESEIACVELAQTSDSSSQKACITHSF-IKANKKKECKDVKLGINNNDKDLKICVIEHTSLSGVENCCCQDLLGMLQENCS 509
ALB01054.1 ANYSSLENYIKDDPYSKEYVTKLSFIPNSSDANNPSGETANHNDEVCNCNESEISSVEHAQTSVLLSQKAYITHSS-IKANKKKVCKYVKLGVRENDKDLKICVIEDDSLRGVENCCFKDFLRILQENCS 510
ALB01057.1 ANYSSLDDYIKGDPYFAEYVTKLSFILNSSDTENASEKIQKNNDEACNCNESEISSVEQEQISGSSSNKTCNTHSS-IKANKKKECKDVKLGVREKDKVLRVCVIEDDSLRGVENCCCQDLLGMLQENCS 510
ALB01040.1 DNYSSLENYIKGDPYFAEYATKLSFILNSSDTENASEKIQKNNDEVCNCNESEIASVEQEQISGPSSNKTCNTHSS-IKTNKKKECKHVKLGINNNDKDLKICVIEDDSLRGVENCCFKDFLRILQENCS 507
ETW41644.1 DNYSSLENYIKGDPYSAEYATKLSFILNSSDTENASEKIQKNNDEVCNCNESEIASVEQAQTSGSSSNKTCITHSS-IKANKKKECKDVKLGINNNDKDLKICVIEDTSLSGVDNCCFKDFLRILQENCS 509
ALB01043.1 DNYSSLENYIKGDPYFAEYATKLSFILNSSDTENASEKIQKNNDEVCNCNESEIASVEQEQISGPSSNKTCNTHSS-IKTNKKKECKHVKLGINNNDKDLKICVIEDDSLRGVENCCFKDFLRILQENCS 507
EUT82883.1 ANYSSLDDYIKGDPYSKEYVTKLSFILNPSDANNPSGETANHNDEACNPNESGIASVEHAQTSGSSSNKTCITHSS-IKTNKKKVCKHVKLGVRENDKDLKICVIEDTSLSGVENCCCQDLLGILQENCS 510
ALB01038.1 DNYSSLDDYIKDDPYFAEYVTKLSFILNSSDTENASEKIQKNNDEACNSNESGISSVGQAQTSCPSSNKTCITHSS-IKANKKKVCKDVKLGVRENDKVLRVCVIEHTSLSGVENCCCQDLLGILQEPRI 512
ALB01058.1 DNYSSLDDYIKDDPYFAEYVTKLSFILNSSDTENASEKIQKNNDEACNSNESGISSVGQAQTSCPSSNKTCITHSS-IKANKKKVCKDVKLGVRENDKVLRVCVIEHTSLSGVENCCCQDLLGILQEPRI 512
ALB01059.1 N-YLSLDDYIKDDPYSAEYGKLLKFD-----NTNAFKESITCNKNVCSCSGNEKLIISEGSSSCSFGTSSSYQSSIITSNKRKECKQIKFSGNKNNMNINICSTQDEKLL--VK--LEELLKSFCNTCD 501
KNG76633.1 NGYSSANSYIKNNPYSAEYGKLLKFD-----GPDAFIESPTYYNNVCCCSTPEKSAKTDGSSCSSSFGTSFSYENS-VTSNKRKECKQIKFSGNKNNMNINICSTQDNNLL--VE--FQELLKSFCNISG 502
ALB01051.1 DNYSSLDDYIKGDPYSSEDGKLLIFD-----NTNAFIESPTYYNNVCCCSTPEKSAKTDGSSCSSSFGTSFSYQSSIITSKKRNECAQIKFSGNKNNMNINICSTQDNNLL--VE--FQELLKGFCDTCD 505
ALB01049.1 NGYSSANSYVKNNPYSAEYGKLLKFD-----GPDAFIESPTYYNNVCCCSTPEKSAKTHGSSSSSSFGTSFSYENS-VTSNKRKECKQIKFSGNKNNMNINICSTQDEKLL--VK--LEDLLKGFCDTCD 505
EWC75604.1 NGYSSANSYIKNNPYSAEYGKLLIFD-----NTNAFKESITFNKNVCSCSGNEKLIISEGSSSRPFGTSFSYENS-VTSNKRKECKQIKFSGNKNNMNINICSTQDEKLL--VK--LEDLLKGFCNISG 500
ALB01045.1 NGYSSANSYVKNNPYSSEDGKLLKFD-----NTNAFKESITCNKNVCSCSGNEKLIISEGSLSSSFGTSSSYQSSIITSKKRNECAQIKFSGNENNMNINICSTQDNNLL--VK--LEDLLKGFCDTCD 500
ALB01047.1 NEYSSANSYVKNNPYSSEDGKLLKFD-----NTNAFKESITCNKNVCSCSGNEKLIISEGSSSSSSFGTSFSYENS-VTSNKRKECKQIKFSGNKNNMNIKICSTQDNNLL--VK--LEDLLKGFCDTCD 505
7G8.pro ANYSSLENYIKDDPYSKEYVTKLSFIPNSSDANTSSEKIQKNNDEVCNPNESGISSVEQAQTSGPSSNKTCITHSS-IKANKKKECKDVKLGVRENDKDLKICVIEDTSLSGVDNCCCQDLLGILQENCS 512
124_8.pro NGYSSANSYVKNDPYSKEYVTKLSFILNPSDANNPSGETANHNDEACNPNESEIASVGQAQTSDRLSQKACITHSF-IGANKKIVCKDVKLGVREKDKDLKICVIEDDSLRGVENCCFKDLLGILQENCS 510
indo.pro DNYKSLDDYIKGDPYSAEYVTKLSFIPNSSDANNPSEKIQKNNDEVCNCNESEISSVGQASISDPSSNKTCNTHSS-IKANKKKVCKDVKLGVRENDKVLKICVIEHTSLRGVDNCCFKDLLGILQEPRI 510
KMWII.pro DNYKSLDDYIKDDPYSKEYTTKLSFILNSSDANTSSGETANHNDEACNCNESEISSVGQAQTSGPSSNKTCITHSF-IKANKKKVCKDVKLGVRENDKVLRVCVIEDTSLSGVDNCCCQDLLGILQENCS 508
M24.pro DNYKSLDDYIKGDPYFAEYATKLSFILNSSDANNPSGETANHNDEVCNPNESEISSVGQAQTSDPSSNKTCNTHSS-IKANKKKVCKHVKLGINNNDKVLKICVIEHTSLSGVENCCFKDLLGILQENCS 510
MTS1.pro DNYSSLDDYIKDDPYSKEYTTKLSFILNSSDANTSSEKIQKNNDEVCNPNESEISSVEQAQTSRPSSNKTCITHSS-IKANKKKVCKDVKLGVRENDKVLRVCVIEHTSLSGVENCCCQDLLGILQENCS 513
P13.pro ANYKSLDDYIKDDPYSAEYATKLSFILNPSDANTSSGETANHNDEVCNCNESEIASVELAPISDSSSNKTCITHSF-IGANKKKECKDVKLGVREKDKDLKICVIEDDSLRGVENCCCQDLLGILQENCS 511
T2C6.pro ANYKSLENYIKDDPYSKEYVTKLSFIPNSSDANTSSEKIQKNNDEVCNPNESGISSVEQAQTSDPSSNKTCITHSS-IKANKKKECKDVKLGVRENDKDLKICVIEHTSLSGVDNCCFKDFLRMLQEPRI 511
V1_S.pro DNYKSLDDYIKDDPYSAQYTTKLSFILNPSDANTSSEKIQKNNDEACNCNESGISSVGQAQTSGPSSNKTCITHSS-IKANKKKVCKDVKLGINNNDKVLKICVIEDTSLSGVDNCCCQDLLGILQENCS 512
WR80.pro NEYSSANSYIKNNPYSSEDGKLLIFD-----NTNAFIESPTYYNNVCCCSTPEKSAKTDGSSSSSSFGTSFSYENS-VTSNKGKECKQIKFSGNKNNMNINICSPPDEKLL--VK--LEDLLKGFCDTCD 501
PFL0030cexon1+2.pro N-YSSLENYIKGDPYFAEYATKLSFILNSSDANNPSEKIQKNNDEVCNCNESGIASVEQEQISDPSSNKTCITHSS-IKANKKKVCKHVKLGVRENDKDLRVCVIEHTSLSGVENCCCQDFLRILQENCS 506
A4PFL0030c exon1+2.pro N-YSSLENYIKGDPYFAEYATKLSFILNPSDANNPSGETANHNDEACNCNESGISSVGQAQTSGPSSNKTCITHSS-IKTNKKKECKDVKLGVRENDKDLKICVIEDTSLSGVDNCCCQDLLGILQENCS 507
Newdd2var2CSAsequence.pro DNYKSLENYIKGDPYFAEYATKLSFILNSSDTENASETPSKYYDEACNCNESEIASVGQAQTSGPSSNKTCITHSS-IKTNKKKECKDVKLGINNNDKVLRVCVIEDTSLSGVDNCCCQDLLGILQENCS 508
McPFL0030c.pro ANYKSLDDYIKGDPYFAEYATKLSFILNSSDANTSSGETANHNDEACNCNESEISSVEHASISDPSSNKTCNTHSS-IKANKKKVCKHVKLGVRENDKDLRVCVIEHTSLSGVENCCFKDFLRILQENCS 509
HB3-1var2CSA.pro NGYSSANSYVKDDPYSAEYVTKLSFILNSSDANTSSETPSKYYDEVCNCNESEISSVGQAQTSGPSSNKTCITHSS-IKTNKKKVCKDVKLGINNNDKVLRVCVIEDTSLSGVDNCCCQDLLGILQENCS 507
HB3-2var2CSA.pro NEYSSANSYVKNNPYSAEYVTKLSFILNSSDANTSSETPSKYYDEVCNCNESEISSVGQAQTSGPSSNKTCITHSS-IKTNKKKVCKDVKLGINNNDKVLRVCVIEDTSLSGVDNCCCQDLLGILQENCS 511
S429 S433

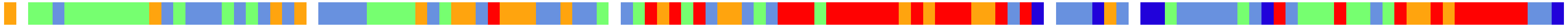

```
                              D-NKRGSSSNGSCDNNNEEACEKNL-EKVLASLTNCYKCDKCKSGTSKKN-KKWIWKKSSGNEEGLQKEYANTIGLPPRTQSLCLG-XCLLEK-VCKTQELKNIDTNSELLKEWIIAAFHEGKNLKTSY-
                                       530       540       550       560       570       580       590       600       610       620       630       640       650

ALB01044.1                    D-NKSGSSSNGSCNNKNQEACEKNL-EKVLASLTNCYKCDKCKSEQSKKNNKNWIWKKSSGKEGGLQKEYANTIGLPPRTQSLCLV-VCLDEK-GKKTQELKNIRTNSELLKEWIIAAFHEGKNLKPS--  632
ALB01053.1                    D-NKSGSSSNGSCNNKNQEACEKNL-EKVLASLTNCYKCDKCKSEQSKKNNKNWIWKKSSGKEGGLQKEYANTIGLPPRTQSLCLV-VCLDEK-GKKTQELKNIRTNSELLKEWIIAAFHEGKNLKPS--  632
ETW56275.1                    D-NKSGSSSNGSCDKNNEEACEKNL-EKVFASLTNCYKCEKCKSEQSKKNNKKWTWRKSSGNKEGLQEEYANTIGLPPRTQSLCLV-VCLDEKEGKKTQELKNIRTNSELLKEWIIAAFHEGKNLKPSHE  636
ALB01048.1                    D-NKSGSSSNGSCDNKNQEACEKNL-EKVLASLTNCYKCDKCKSEQSKKNNNIWIWRKSSGKEGGLQKEYANTIGLPPRTQSLCLV-VCLDEK-GKKTQELKNIRTNSELLKEWIIAAFHEGKNLKPS--  634
ALB01041.1                    D-NKRRSSSNGSCDNKNQDECEKNL-EKVLASLTNCYKCDKCKSGTSTVN-NKWIWKKYSVKEGGLQKEYANTIALPPRTQSLYL--VCLHEK-EGKTQELKNIRTNSELLKEWIIAAFHEGKNLKIS--  636
ALB01039.1                    D-NKRRSSSNGSCDNKNQDECEKNL-EKVLASLTNCYKCDKCKSGTSTVN-NKWIWKKYSVKEGGLQKEYANTIALPPRTQSLYL--VCLHEK-EGKTQELKNIRTNSELLKEWIIAAFHEGKNLKIS--  636
ALB01056.1                    D-NIRGSSSNGSCDKNSEEACEKNL-EKVFASLTNGYKCDKCKSGTSTVN-NKWIWKKYSGKEGGLQKEYANTIALPPRTQSLCLV-VCLDEK-EGKTQELKNIRTNSELLKEWIIAAFHEGKNLKTTY-  638
KNC35427.1                    D-NKRGSSSNDSCNNNNEEACEKNL-DEALASLTNGYKCDKCKSGTSTVN-KKWTWRKSSGNEEGLQKEYANTIGLPPRTQSLCL--VCLHEK-EGKTKH-KTISTNSELLKEWIIAAFHEGKNLKTSH-  636
ALB01046.1                    D-NKRESSSNGSCNNKNQDECQKKL-DEALASLTNGYKCEKCKSEQSKKNKKIWTWKKSSGNKEGLQEEYANTIGLSPRTQSLCL--VCLHEK-EGKTQELKNISTNSELLKEWIIAAFHEGKNLKPS--  633
ALB01037.1                    D-NKRESSSNGSCDKNNEEACEKNL-EKVFASLTNGYKCDKCKSEQSKKNNKNWIWKKYSGNGEGLQKEYANTIGLPPRTHSLYLGNLRKLEN-VCEDVKDINFDTKEKFLAGCLIAAFHEGKNLKTS--  637
ALB01055.1                    DKNQSGSSSNGSCDKNSEEACEKNL-DEALASLTNGYKCEKCKSEQSKKNNKIWTWRKFPGNGEGLQKEYANTIGLSPRTQLLYL--VCLHEK-GKKTQELKNIRTNSELLKEWIIAAFHEGKNLKKR--  635
AAQ73924.1                    D-NKRGSSSNGSCDKNSEEICQKKL-EKVFASLTNGYKCDKCKSGTSRSK-KKWIWKKSSGNEEGLQKEYANTIGLPPRTQSLCL--VCLHEK-EGKTQH-KTISTDSELLKEWIIAAFHEGKNLKTSH-  631
ALB01042.1                    D-NKRESSSNGSCNDKNQDECQKKL-EKVFASLTNCYKCEKCKSGTSR-SKKIWIWKKYSGNGEGLQKEYANTIGLPPRTQSLCLV-VCLDEK-GKKTQELKNIRTNSELLKEWIIAAFHEGKNLKTT--  634
AJD77410.1                    D-NKRGSSSNGSCDKNSEEACEKNL-DEALASLTNGYKCDKCKSGTSTVN-KNWIWKKYSGKEGGLQEEYANTIGLPPRTQSLCLV-VCLHEK-EGKTQELKNISTNSELLKEWIIAAFHEGKNLKPSH-  639
EWC75615.1                    DKNQSGSSSNGSCDKNNEDECQKKL-EKVLASLTNCYKNQKCKSGTSTVN-KNWIWKKYSGTEGGLQKEYANTIALPPRTQSLCL--VCLHEK-EEKTQELKNISTNSELLKEWIIAAFHEGKNLKKRY-  634
ALB01054.1                    D-NKRESSSNGSCNNNNEEACEKNL-DEALASLTNCYKNQKCKSGTSTVNN-KWIWKKSSGKEGGLQKEYANTIALPPRTQSLCLV-VCLDEK-GKKTQELKNISTNSELLKERIIASFHEGKNLKTSHE  635
ALB01057.1                    D-NKSGSSSNDSCDKNNEDECQKKL-EKVFASLTNGYKCEKCKSEQSKKNNNKWIWRKFPGNGEGLQKEYANTIGLPPRTHSLYLGNLPKLEN-VCEDVTDINFDTKEKFLAGCLIAAFHEGKNLKTTY-  637
ALB01040.1                    D-NKRESSSNGSCNNKNQEACEKNL-DEALASLTNCYKCEKCKSGTSTVN-KNWIWKKSSGDEKGLQKEYANTIGLPPRTHSLYLGNLPKLEN-VCEDVTDINFDTKEKFLAGCLIAAFHEGKNLKKRY-  633
ETW41644.1                    D-NKRGSSSNGSCNNNNEEICQKKL-EKVFASLTNGYKCDKCKSGTSTVN-KNWIWKKSSGNGEGLQKEYANTIGLPPRTQSLCL--VCLHEK-EGKTQELKNISTNSELLKEWIIAAFHEGKNLKTSH-  633
ALB01043.1                    D-NKRESSSNGSCNNKNQEACEKNL-DEALASLTNCYKCEKCKSGTSTVN-KNWIWKKSSGDEEGLQEEYANTIGLPPRTHSLYLGNLPKLEN-VCEDVTDINFDTKEKFLAGCLIAAFHEGKNLKKRY-  633
EUT82883.1                    D-NKRRSSSNGSCDNKNQDECEKNL-EKVLASLTNCYKCDKCKSGTSTVN-NKWIWKKYSGKEGGLQKEYANTIGLPPRTQSLYLGNLPKLEN-VCEDVTDINFDTKEKFLAGCLIAAFHEGKNLKTTY-  636
ALB01038.1                    D------------------------------------KNQKCKSEQSKKNNNKWIWKKYSGKEGGLQKEYANTIGLPPRTQSLYLGNLPKLEN-VCKDVTDINFDTKEKFLAGCLIAAFHEGKNLKTTY-  605
ALB01058.1                    D------------------------------------KNQKCKSEQSKKNNNKWIWKKYSGKEGGLQKEYANTIGLPPRTQSLYLGNLPKLEN-VCKDVTDINFDTKEKFLAGCLIAAFHEGKNLKTTY-  605
ALB01059.1                    I--GIG--VEVVCENNCEEQYKKLLPCLEKCTVLNCNECNKTRCKPLKKEQEKWIWKKYSVKEGGLQEEYANTIALPPRTQSLCLVCLHEKEE---KTQELKNISTNSELLKERIIAAFHEGKNLKTTY-  624
KNG76633.1                    V--NTGG-VEVVCENNCEEQYKKLLPSLEKCTILTCKECNKTRCKPLKKQQEKWIWKKFPGNGEGLQKEYANTIGLPPRTQSLCLVCLHEKEG-KTQELKNIS--TNSELLKEWIIAAFHEGKNLKTSH-  626
ALB01051.1                    T--DIG--VEVVCENNCEEQYKKLLPCLEKCTVLNCNECNKTRCKPLKKEQEKWIWKKYSGKEGGLQKEYANTIGLPPRTQSLCLVCLHEKE----GKTQHKTISTNSELLKEWIIDAFHEGKNLKTTY-  627
ALB01049.1                    T--DIG--VEVVCEENCEEQFKKLLPSLEKCTILTCKECNKTRCKPLKKVEEKWIWKKSSGNEKGLQKEYANTIGLPPRTQSLCLVCLHEKE----GKTQHKTISTNSELLKEWIIAAFHEGKNLKTTY-  627
EWC75604.1                    V--NTGG-VEVVCENNCEEQYKKLLPCLEKCTILTCNECNKTRCKPLKKVEEKWIWKKSSGNGEGLQKEYANTIGLPPRTQSLCLVCLHEKE----GKTQHKTISTNSELLKEWIIAAFHEGKNLKKRY-  623
ALB01045.1                    T--GIG--VEVVSENNCEEQYKKLLPSLEKCTVLNCNECNKTRCKPLKKQQEKWIWGKPKQEAAGLQEEYANTIGLPPRTQSLYLGNLRKLEN-VSKGVTDINFDTKEKFLAGCLIAAFHEGKNLKTSH-  625
ALB01047.1                    T--DIG--VEVVSENNCEEQYKKLLPCLEKCTVLNCNECNKTVFKNLKKQQEKWIWGKPKQEAAGLQKEYANTIGLPPRTQSLYLGNLPKLGN-VCKGVTDINFDTKEKFLAGCLIAAFHEGKNLKTTY-  630
7G8.pro                       D-NKRGSSSNDSCDNKNQDECQKKL-DEALESLHNGYKNQKCKSGTSTVN-KKWIWKKSSGNKEGLQKEYANTIGLPPRTQSLYLGNLPKLEN-VSKGVTDIIYDTKEKFLAGCLIVSFHEGKNLKTS--  637
124_8.pro                     D-NKSGSSSNGSCNNKNQDECQKKL-DEALASLHNGYKCDKCKSGTSRSK-KIWTWRKFPGNGEGLQKEYANTIGLPPRTQSLYLGNLRKLEN-VCKGVTDINFDTKEKFLAGCLIAAFHEGKNLKISN-  636
indo.pro                      DKNQSGSSSNGSCDKNSEEACEKNL-EKVLASLTNGYKCDKCKSGTSR-SKKKWIWKKYSGKEGGLQEEYANTIGLPPRTQSLCLV-VCLDEK-EGKTQELKNISTNSELLKEWIIAAFPEGKNLKPS--  635
KMWII.pro                     D-NKRGSSSNGSCNNKNQDECQKKL-EKVFVSLTNGYKCDKCKSGTSTVN-KKWIWKKSSGNEKGLQKEYANTIGLPPRTQSLYLGNLPKLGN-VCEDVTDINFDTKEKFLAGCLIAAFHEGKNLKISH-  634
M24.pro                       D-NKSGSSSNGSCNNKNQEACEKNL-EKVLASLTNCYKCDKCKSGTSTVN-KNWIWKKSSGNKEGLQKEYANTIGLPPRTHSLYLGNLPKLEN-VCEDVKDINFDTKEKFLAGCLIAAFHEGKNLKKRY-  636
MTS1.pro                      D-NKRGSSSNGSCDKNSEEACEKNL-DEALASLTNCYKNQKCKSEQSKKNNNKWIWKKSSGKEGGLQKEYANTIGLPPRTQSLYLGNLPKLEN-VCKGVTDINFDTKEKFLAGCLIAAFHEGKNLKTTY-  640
P13.pro                       D-NKSGSSSNGSCDKNSEDECQKKL-ENVFASLKNGYKCDKCKSGTSTVN-KKWIWRKYSGNGEGLQKEYANTIGLPPRTHSLYL--VCLHEK-EGKTQH-KTISTNSELLKEWIIAAFHEGKNLKTS--  633
T2C6.pro                      DKNQRGSSSNGSCDKNSEEACEKNL-DEALASLTNGYKCDKCKSEQSKKNNNKWIWKKFPGKEGGLQEEYANTIGLPPRTQYLCLV-VCLDEK-EGKTQELKNIRTNSELLKEWIIAAFHEGKNLKTTY-  638
V1_S.pro                      D-NKRGSSSNGSCNNNNEEACEKNL-DEAPASLHNGYKNQKCKSGTSRSK-KKWIWKKSSGNEKGLQEEYANTIGLPPRTQSLCL--VCLHEK-EGKTQH-KTISTNSELLKEWIIAAFHEGKNLKTSH-  635
WR80.pro                      P--DIG--VEVVSEENCEEQYKKLLPSLEKCTILTCKECNKTRCKPLKKVEEKWIWKKSSGNGEGLQKEYANTIGLPPRTQSLYLGNLPKLEN-VCKGVTDINFDTKEKFLAGCLIAAFHEGKNLKTSP-  626
PFL0030cexon1+2.pro           D-NKSGSSSNGSCNNKNQEACEKNL-EKVLASLTNCYKCDKCKSEQSKKNNKNWIWKKSSGKEGGLQKEYANTIGLPPRTQSLCLV-VCLDEK-GKKTQELKNIRTNSELLKEWIIAAFHEGKNLKPS--  631
A4PFL0030c exon1+2.pro        D-NKRGSSSNDSCDNKNQDECQKKL-EKVFASLTNGYKCDKCKSGTSRSK-KKWIWKKSSGNEEGLQEEYANTIGLPPRTQSLYLGNLPKLEN-VCEDVKDINFDTKEKFLAGCLIVSFHEGKNLKKRY-  633
Newdd2var2CSAsequence.pro     D-NKRGSSSNGSCDKNSEEICQKKL-EKVFASLTNGYKCDKCKSGTSRSK-KKWIWKKSSGNEEGLQKEYANTIGLPPRTQSLCL--VCLHEK-EGKTQH-KTISTNSELLKEWIIAAFHEGKNLKTSH-  631
McPFL0030c.pro                D-NKSGSSSNGSCDKNNEEACEKNL-EKVFASLTNCYKCEKCKSEQSKKNNKKWTWRKSSGNKGGLQEEYANTIGLPPRTQSLCLV-VCLDEKEGKKTQELKNIRTNSELLKEWIIAAFHEGKNLKPSHE  636
HB3-1var2CSA.pro              DKNQSGSSSNGSCNNKNQDECQKKL-EKVFASLTNGYKCDKCKSGTSRSK-KKWIWRKSSGNEEGLQKEYANTIGLPPRTQSLYLGNLPKLEN-VCKGVTDIIYDTKEKFLSGCLIAAFHEGKNLKTSH-  634
HB3-2var2CSA.pro              DKNQSGSSSNGSCNNKNQDECQKKL-EKVFASLTNGYKCDKCKSGTSRSK-KKWIWRKSSGNEEGLQKEYANTIGLPPRTQSLYLGNLRKLEN-VCKGVTDINFDTKEKFLAGCLIAAFHEGKNLKTTY-  638
```

```
                            -----PXKKNDDNGKKLCKALKYSFADYGDLIKGTSIWDNEYTKDLELNLQKIFGKLFRKYIKKNXTAEQDTSYSSLDELRESWWNTNKKYIWXAMKHGAGMNGTTCCGD--------------GSVT-
                                      660       670       680       690       700       710       720       730       740       750       760       770       780

ALB01044.1                  -----HEKKNDDNGKKLCKALEYSFADYGDLIKGTSIWDNEYTKDLELNLQKIFGKLFRKYIKKNNTAEQDTSYSSLDELRESWWNTNKKYIWLAMKHGAGMNSTTCCGD--------------GSVT-  741
ALB01053.1                  -----HEKKNDDNGKKLCKALEYSFADYGDLIKGTSIWDNEYTKDLELNLQKIFGKLFRKYIKKNNTAEQDTSYSSLDELRESWWNTNKKYIWLAMKHGAGMNSTTCCGD--------------GSVT-  741
ETW56275.1                  KKNDDNGKKNDDNNSKLCKDLKYSFADYGDLIKGTSIWDNEYTKDLELNLQKIFGKLFRKYIKKNIASDENTLYSSLDELRESWWNTNKKYIWLAMKHGAEMNGTTCNAD--------------GSVT-  751
ALB01048.1                  -----LEKKKGDNNSKLCKDLKYSFADYGDLIKGTSIWDNEYTKNVELNLQNNFGKLFRKYIKKNIASDENTSYSSLDELRESWWNTNKKYIWTAMKHGAGMNGTMCNAD--------------GSVT-  743
ALB01041.1                  -----PQNKNDDNGKKLCKALKYSFADYGDLIKGTSIWDNDFTKDLELNLQKIFGKLFRKYIKKNISTEQDTLYSSLDELRESWWNTNKKYIWTAMKHGAEMNGTTCCGD--------------GSVTG 745
ALB01039.1                  -----PQNKNDDNGKKLCKALKYSFADYGDLIKGTSIWDNDFTKDLELNLQKIFGKLFRKYIKKNISTEQDTLYSSLDELRESWWNTNKKYIWTAMKHGAEMNGTTCCGD--------------GSVTG 745
ALB01056.1                  -----PQNKNAENKKKLCKALKYSFADYGDLIKGTSIWDNDFTKDLELNLQQIFGKLFRKYIKKNNTIVENTSYSSLDELRESWWNTNKKYIWTAMKHGAEMNGTMCNAD--------------GSVTG 747
KNC35427.1                  ------EKKNDDNGKKLCKALEYSFADYGDLIKGTSIWDNEYTKDLELNLQKAFGKLFRKYIKKNNTAEQDTSYSSLDELRESWWNTNKKYIWTAMKHGAEMNGTTCSSGSGDNGDSSITGSSDSGSTT- 759
ALB01046.1                  ----HENKN--DNGKKLCKDLKYSFADYGDLIKGTSIWDNEYTKDLELNLQKIFGKLFRKYIKKNNTIVENTSYSSLDELRESWWNTNKKYIWLAMKHGAGMNSTTCNAD--------------GSVTG 741
ALB01037.1                  -----HEKKKGDNGKKLCKDLKYSFADYGDLIKGTSIWDNEYTKDLELNLQQIFGKLFRKYIKKNNTIVENTSYSSLDELRESWWNTNKKYIWLAMKHGAEMNSTTCNAD--------------GSVT-  746
ALB01055.1                  ----YLEKKKGDNNSKLCKDLKYSFADYGDLIKGTSIWDNDFTKDLELNLQQIFGKLFRKYIKKNNTAEQDTLYSSLDELRESWWNTNKKYIWTAMKHGAGMNSTTCCGD--------------GSVTG 745
AAQ73924.1                  ------EKKNDDNGKKLCKALEYSFADYGDLIKGTSIWDNEYTKDLELNLQKIFGKLFRKYIKKNNTAEQHTSYSSLDELRESWWNTNKKYIWTAMKHGAGMNGTTCS--------------------- 733
ALB01042.1                  ----YPQKNDDNNSKLCKDLKYSFADYGDLIKGTSIWDNDFTKDLELNLQKIFGKLFRKYIKKNNTIVENTSYSSLDELRESWWNTNKKYIWTAMKHGAGMNSTMCNAD--------------GSVTG 744
AJD77410.1                  ------EKKNAENKKKLCKALKYSFADYGDLIKGTSIWDNDFTKDLELNLQKIFGKLFRKYIKKNIASDENTSYSSLDELRESWWNTNKKYIWLAMKHGAGMNGTTCSS-------------------- 742
EWC75615.1                  -----HQNNNSGNKKKLCKALKYSFADYGDLIKGTSIWDNDFTKNLELNLQKIFGKLFRKYIKKNISTEQHTSYSSLDELRESWWNTNKKYIWTAMKHGAGMNGTTCCGD--------------GSVTG 743
ALB01054.1                  KKNADNRKKNADNNSKLCKALKYSFADYGDLIKGTSIWDNEYTKDLELNLQNNFGKLFRKYIKKNNTAEQHTSYSSLDELRESWWNTNKKYIWLAMKHGAGMNGTTCSS-------------------- 745
ALB01057.1                  -----LEKKKGDNGKKLCKALKYSFADYGDLIKGTSIWDNEYTKDLELNLQKIFGKLFRKYIKKNNTAEQHTSYSSLDELRESWWNTNKKYIWLAMKHGAEMNGTMCNAD--------------GSVT-  746
ALB01040.1                  -----PQNKNDDNNSKLCKDLKYSFADYGDLIKGTSIWDNEYTKDLELNLQKAFGKLFGKYIKKNNTAEQDTSYSSLDELRESWWNTNKKYIWLAMKHGTTCSSGTCDNGD--------------GSVT-  743
ETW41644.1                  ------QKKNDDNGKKLCKALEYSFADYGDLIKGTSIWDNEYTKDLELNLQKIFGKLFRKYIKKNNTIVENTSYSSLDELRESWWNTNKKYIWTAMKHGAGMNGTTCSS--G----------------- 737
ALB01043.1                  -----PQNKNDDNNSKLCKDLKYSFADYGDLIKGTSIWDNEYTKDLELNLQKAFGKLFGKYIKKNNTAEQDTSYSSLDELRESWWNTNKKYIWLAMKHGTTCSSGSGDNGD--------------GSVT-  743
EUT82883.1                  -----LEKKNADNNSKLCKALKYSFADYGDLIKGTSIWDNEYTKDLELNLQNNFGKLFRKYIKKNIASDENTLYSSLDELRESWWNTNKKYIWLAMKHGAGMNG-TTCSSG--------------SGDN-  745
ALB01038.1                  -----PQNKNAENKKKLCKDLKYSFADYGDLIKGTSIWDNDFTKDLELNLQKAFGKLFRKYIKKNISTEQHTLYSSLDELRESWWNTNKKYIWTAMKHGAEMNITTCCGD--------------GSVT-  714
ALB01058.1                  -----PQNKNAENKKKLCKDLKYSFADYGDLIKGTSIWDNDFTKDLELNLQKAFGKLFRKYIKKNISTEQHTLYSSLDELRESWWNTNKKYIWTAMKHGAEMNITTCCGD--------------GSVT-  714
ALB01059.1                  -----PQNNNSGNKENLCKALKYSFADYGDLIKGTSIWDNDFTKDLELNLQKAFGKLFRKYIKKNIASDENTSYSSLDELRESWWNTNKKYIWLAMKHGAGMNGTTCCG--------------DGSVT-  733
KNG76633.1                  -----Q-NNNSGNKENLCKALKYSFADYGDLIKGTSIWDNDFTKDLELNLQKIFGKLFRKYIKKNISTEQHTLYSSLDELRESWWNTNKKYIWLAMKHGAGMNSTTCS--------------------- 728
ALB01051.1                  -----PQKKNADNGKKLCKALKYSFADYGDLIKGTSIWDNDFTKDLELNLQKIFGKLFRKYIKKNISTEQHTLYSSLDELRESWWNTNKKYIWTAMKHGAGMNGTTCS--------------------- 730
ALB01049.1                  -----PQNKNDDNNSKLCKALEYSFADYGDLIKGTSIWDNEYTKDLELNLQKAFGKLFRKYIKKNNTAEQHTSYSSLDELRESWWNTNKKYIWTAMKHGAGMNGTTCS--------------------- 730
EWC75604.1                  -----PQNNNSGNKENLCKALKYSFADYGDLIKGTSIWDNDFTKNVELNLQQIFGKLFRKYIKKNISTEQHTSYSSLDELRESWWNTNKKYIWTAMKHGAGMNGTTCS--------------------- 726
ALB01045.1                  -----E-NKNDDNGKKLCKALKYSFADYGDLIKGTSIWDNEYTKDLELNLQKAFGKLFRKYIKKNNTAEQDTSYSSLDELRESWWNTNKKYIWLAMKHGAGMNGTTCCGDGS-----VTGSSDSGSTT- 742
ALB01047.1                  -----P-----QNKKKLCKDLKYSFADYGDLIKGTSIWDNEYTKDLELNLQKAFGKLFRKYIKKNISTEQHTLYSSLDELRESWWNTNKKYIWTAMKHGAEMNSTMCN--------------ADGSVT-  734
7G8.pro                     -----HEKKNDDNGKKLCKALEYSFADYGDLIKGTSIWDNEYTKDLELNLQKAFGKLFRKYIKKNISAEQDTSYSSLDELRESWWNTNKKYIWIAMKHGAGMNGTTCCGDG--------------SSGE-  747
124_8.pro                   ------KKKNDDNGKKLCKDLKYSFADYGDLIKGTSIWDNEYTKDLELNLQKIFGKLFRKYIKKNIASDENTLYSSLDELRESWWNTNKKYIWLAMKHGTTCSSGSGDNGD--------------GSVT-  745
indo.pro                    -----PEKKKGDNGKKLCKDLKYSFADYGDLIKGTSIWDNEYTKDLELNLQKIFGKLFRKYIKKNIASDENTLYSSLDELRESWWNTNKKYIWLAMKHGAGMNSTMCNAD--------------GSVT-  744
KMWII.pro                   ------EKKKGDNGKKLCKALEYSFADYGDLIKGTSIWDNEYTKDLELNLQKAFGKLFGKYIKKNIASDENTSYSSLDELRESWWNTNKKYIWTAMKHGAEMNS-TMCNAD--------------GSVT-  742
M24.pro                     -----PQNKNDDNNSKLCKALEYSFADYGDLIKGTSIWDNEYTKDLELNLQQIFGKLFRKYIKKNISTEQDTLYSSLDELRESWWNTNKKYIWLAMKHGAGMNITTC-CGD--------------GSVT-  745
MTS1.pro                    -----LEKKNDDNGKKLCKALEYSFADYGDLIKGTSIWDNEYTKDLELNLQKAFGKLFRKYIKKNNTAEQDTSYSSLDELRESWWNTNKKYIWTAMKHGAGMNGTTCSS--------------GSGD-  748
P13.pro                     -----HQNNNSGNKKKLCKALKYSFADYGDLIKGTSIWDNDFTKDLELNLQKIFGKLFRKYIKKNIASDENTSYSSLDELRESWWNTNKKYIWTAMKHGAEMNSTMCNGD--------------GSVTG 742
T2C6.pro                    -----PQKKNDDNGKKLCKDLKYSFADYGDLIKGTSIWDNEYTKNVELNLQNNFGKLFRKYIKKNNTAEQDTSYSSLDELRESWWNTNKKYIWLAMKHGAEMNSTTCCGD--------------GSVT-  747
V1_S.pro                    ------EKKNDDNGKKLCKALEYSFADYGDLIKGTSIWDNEYTKDLELNLQKAFGKLFRKYIKKNNTAEQDTSYSSLDELRESWWNTNKKYIWIAMKHGAGMNGTTCS--------------------- 737
WR80.pro                    -----EKKK-GENKRKLCKALEYSFADYGDLIKGTSIWDNEYTKDLELNLQNNFGKLFRKYIKKNISTEQHTSYSSLDELRESWWNTNKKYIWTAMKHGAEMNGTTCSSG---------SGDNGDSSIT- 740
PFL0030cexon1+2.pro         -----HEKKNDDNGKKLCKALEYSFADYGDLIKGTSIWDNEYTKDLELNLQKIFGKLFRKYIKKNNTAEQDTSYSSLDELRESWWNTNKKYIWLAMKHGAGMNSTTCCGD--------------GSVT-  740
A4PFL0030c exon1+2.pro      -----PQNKNSGNKENLCKALEYSFADYGDLIKGTSIWDNEYTKDLELNLQNNFGKLFGKYIKKNNTAEQDTSYSSLDELRESWWNTNKKYIWTAMKHGAEMNITTC-NAD--------------GSVT-  742
Newdd2var2CSAsequence.pro   ------EKKNDDNGKKLCKALEYSFADYGDLIKGTSIWDNEYTKDLELNLQKIFGKLFRKYIKKNNTAEQHTSYSSLDELRESWWNTNKKYIWTAMKHGAGMNGTTCS--------------------- 733
McPFL0030c.pro              KKNDDNGKKNDDNNSKLCKDLKYSFADYGDLIKGTSIWDNEYTKDLELNLQKIFGKLFRKYIKKNIASDENTLYSSLDELRESWWNTNKKYIWLAMKHGAEMNGTTCNAD--------------GSVT-  751
HB3-1var2CSA.pro            ------EKKNDDNGKKLCKALEYSFADYGDLIKGTSIWDNDFTKDLELNLQKIFGKLFRKYIKKNNTAEQDTSYSSLDELRESWWNTNKKYIWTAMKHGAGMNS-TTCSGD--------------GSVT-  742
HB3-2var2CSA.pro            ------PQ----NKKKLCKDLKYSFADYGDLIKGTSIWDNEYTKDLELNLQKAFGKLFRKYIKKNISTEQHTLYSSLDELRESWWNTNKKYIWLAMKHGAGMNS-TTCCGD--------------GSVT-  742
```

```
                             ----------------GSGS----S-------------CDDIPTIDLIPQYLRFLQEWVEHFCKQRQAKVKDVIENCKSCKES------GGTCNSDCKTKCKNECEAYKKFIEEC---TAAG-----G--
                                        790       800       810       820       830       840       850       860       870       880       890       900       910

ALB01044.1                   ----------------GSGS----S-------------CDDIPTIDLIPQYLRFLQEWVEHFCKQRQEKVKPVIENCKSCKES------GGTCNGECKTECKNKCEVYKKFIEECG---TAA----GG-- 823
ALB01053.1                   ----------------GSGS----S-------------CDDIPTIDLIPQYLRFLQEWVEHFCKQRQEKVKPVIENCKSCKES------GGTCNGECKTECKNKCEVYKKFIEELW---TAA----GG-- 823
ETW56275.1                   ----------------GSGS----S-------------CDDIPTIDLIPQYLRFLQEWVEHFCKQRQAKVKDVIENCKSCKES----------GNKCKTECKNKCEAYKKFIENCK---GG-----DG-- 828
ALB01048.1                   ----------------GSGS----S-------------CDDIPTIDLIPQYLRFLQEWVEHFCKQRQEKVKPVIENCKSCKNTSSKTKLGGTCNGECKTECKNKCEAYKTFIEEFR---TAA----GG-- 831
ALB01041.1                   SSDSGS--------TTCSGDNGSIS-------------CDDIPTIDLIPQYLRFLQEWVEHFCKQRQENVNAVIENCNSCKES------GGTCNSDCEKKCKIECEKYKNFIEECNG-TAAG-----G-- 841
ALB01039.1                   SSDSGS--------TTCSGDNGSIS-------------CDDIPTIDLIPQYLRFLQEWVEHFCKQRQENVNAVIENCNSCKES------GGTCNSDCEKKCKIECEKYKNFIEECNG-TAAG-----G-- 841
ALB01056.1                   SSDSGS--------TTCSGDNGSIS-------------CDDMPTIDLIPQYLRFLQEWVEHFCKQRQEKVNAVITNCKSCKES------GGTCGSDCKTKCKGECEAYKTFIEKCNG-TAAE-----G-- 843
KNC35427.1                   ----------------CSGDNGSIS-------------CDDIPTTDFIPQYLRFLQEWVEHFCEQRQAKVKDVINSCNSCNES------GGTCNGECKTKCKDECEKYKKFIEDCN---GG-----DG-- 844
ALB01046.1                   SSDSGSTTCCGDGSVTGSGS----S-------------CDDIPTIDLIPQYLRFLQEWVEHFCEQRQAKVKDVIENCKSCKNTSGERKIGGTCGSDCKTKCKNKCDAYKKFIDGTG---SGGGSRPTG-- 850
ALB01037.1                   ----------------GSGS----S-------------CDDIPTIDLIPQYLRFLQEWVEHFCEQRQAKVNAVITNCKSCKES------GGTCGSDCKTKCKDECEKYKNFIEECR--TAAD-----G-- 828
ALB01055.1                   SSDSGSTTCSGD--N-GS-I----S-------------CDDIPTIDLIPQYLRFLQEWVEHFCKQRQAKVKDVITNCKSCKES------GGTCNSDCKTKCKGECEKYKTFIEHCK---GVGG---TG-- 841
AAQ73924.1                   ----------------CSGD----S-------------SNDMPTIDLIPQYLRFLQEWVEHFCKQRQEKVNAVIENCNSCKES------GGTCNSDCKTECKNKCEAYKEFIEDCK---GGG----TG-- 815
ALB01042.1                   SSDSGSTTCSGD---NGSGS----S-------------CDDIPTIDLIPQYLRFLQEWVEHFCKQRQEKVNAVITNCKSCKES------GGTCNSDCKTECKNKCEAYKNFIEDCK---GGG----TG-- 840
AJD77410.1                   ----------------GSGD----S-------------SNDIPTIDLIPQYLRFLQEWVEHFCEQRQAKVKPVIENCNSCKNTSSKTKIGDTCGNDCKTECKNKCEKYKKFIEECR---TAA----EG-- 830
EWC75615.1                   SSDSGSTTCCGDGSVTGSGS----S-------------CDDIPTIDLIPQYLRFLQEWVEHFCEQRQAKVKDVIKNCNSCKECGG--TCNGECKTECEKKCKGECEAYKKFIEKCN---GGG-----GEG 846
ALB01054.1                   ----------------GSGD----S-------------SNDIPTTDFIPQYLRFLQEWVEHFCKQRQEKVNAVIENCKSCKES----------GNKCKTECEKKCEAYKKFIEKCN---GGAA---EG-- 824
ALB01057.1                   ----------------GSGS----S-------------CDDIPTIDLIPQYLRFLQEWVEHFCEQRQGKVKHVMESCKSCKNTSGERKIGGTCNGDCKTECKNKCDAYKTFIEECR--TAAE-----G-- 834
ALB01040.1                   ----------------GSSD----SGSTTCSGDNGSISCDDMPTIDLIPQYLRFLQEWVEHFCKQRQAKVKDVIKNCNSCKNTSGERIIGGTCGSDCKTKCKVACDAYKKFIEKC--GTAAD-----G-- 844
ETW41644.1                   ----------------DNGD----S-------------SNDIPTTDFIPQYLRFLQEWVEHFCEQRQGKVKHVMESCKSCKNTSSKTKLGGKCGNDCEKKCKDECEKYKTFIEECV---TAA----GG-- 825
ALB01043.1                   ----------------GSSD----SGSTTCSGDNGSISCDDMPTIDLIPQYLRFLQEWVEHFCKQRQAKVKDVIKNCNSCKNTSGERIIGGTCGSDCKTKCKVACDAYKKFIEKC--GTAAD-----G-- 844
EUT82883.1                   ----------------GS-I----S-------------CDDIPTIDLIPQYLRFLQEWVEHFCKQRQAKVKDVIKNCNSCKES----------GNKCKTECKNKCEAYKKFIEEF--CTAAG-----G-- 822
ALB01038.1                   ----------------GSGS----S-------------CDDMPTTDFIPQYLRFLQEWVEHFCKQRQAKVNAVIENCNSCKNTSSKTKLGGKCGSDCEKKCKVACDAYKTFIEECG--TAVG-----G-- 802
ALB01058.1                   ----------------GSGS----S-------------CDDMPTTDFIPQYLRFLQEWVEHFCKQRQAKVNAVIENCNSCKNTSSKTKLGGKCGSDCEKKCKVACDAYKTFIEECG--TAGG-----G-- 802
ALB01059.1                   ----------------GSGS----S-------------CDDIPTIDLIPQYLRFLQEWVEHFCEQRQEKVKHVMESCKSCKEC------GGTCGSDCEKKCK----AYKEFIEKCKG-QAAE-----G-- 812
KNG76633.1                   ----------------CSGD-----------------SSDDIPTIDLIPQYLRFLQEWVEHFCKQRQAKVNAVITNCKSCKES------GNKCKTECKTECKNKCEVYKTFIEKCG--TAAD-----G-- 810
ALB01051.1                   ----------------CSGD----SSS----GENQTNSCDDIPTIDLIPQYLRFLQEWVEHFCEQRQGKVKDVIKNCKSCKECGG--TCNGECKTECKTKCKGECEKYKEFIEECKG-KAAE-----G-- 826
ALB01049.1                   ----------------CSGD----S-------------SNDIPTTDFIPQYLRFLQEWVENFCEQRQAKVKDVINSCNSCKES------GGTCGSDCEKKCKDECEKYKTFIEEFC--TAAG-----G-- 812
EWC75604.1                   ----------------CSGS----S-------------CDDIPTIDLIPQYLRFLQEWVEHFCKQRQEKVNAVITNCKSCKNTSSKTKIGDTCNSDCEKKCKDECEKYKTFIEACG--TAVG-----G-- 814
ALB01045.1                   ----------------CCGD----GSV-----TGSGSSCDDIPTIDLIPQYLRFLQEWVEHFCEQRQAKVKDVIKNCKSCKNTSGERKIGDTCGNDCEKKCKVACDAYKEFIEECR--TAAE-----G-- 838
ALB01047.1                   ----------------GSGS----S-------------CDDIPTIDLIPQYLRFLQEWVEHFCKQRQEKVNAVINSCNSCKNTSSKTKLGGKCGSDCEKKCKIECEKYKKFIEEYV--TAVG-----G-- 822
7G8.pro                      ----------------NQTN----S-------------CDDIPTIDLIPQYLRFLQEWVEHFCEQRQAKVKDVITNCKSCKNTSGERKIGGTCNGECKTKCKNKCEAYKTFIEHC---KGGD-----G-- 834
124_8.pro                    ----------------GSGS----S-------------CDDMSTIDLIPQYLRFLQEWVEHFCKQRQEKVKDVIENCKSCKNTSGERIIGGTCGSDCKTKCKGECDAYKNFIEE---CKRGD-----G-- 832
indo.pro                     ----------------GSGS----S-------------CDDMPTIDLIPQYLRFLQEWVEHFCKQRQEKVKPVIENCNSCKNTSSERKIGGTCNSDCKTECKNKCEVYKKFIEDCK----------GGDG 830
KMWII.pro                    ----------------GSGS----S-------------CDDIPTTDFIPQYLRFLQEWVEHFCKQRQEKVNAVIENCNSCKNTSGERKIGGTCNGDCKTECKNKCEAYKNFIED---CKGGD-----G-- 829
M24.pro                      ----------------GSGS----S-------------CDDIPTIDLIPQYLRFLQEWVEHFCKQRQEKVKDVINSCNSCKNTSSKTKLGDTCNSDCEKKCKIECEKYKKFIEEC--RTAVG-----G-- 833
MTS1.pro                     ------------------------S-------------SNDIPTTDFIPQYLRFLQEWVENFCEQRQAKVKDVIENCNSCKNTSGERKIGDTCNSDCEKKCKDECEKYKKFIEDC---KGGD-----G-- 831
P13.pro                      SSDSGS--------TTCSGDNGSIS-------------CDDIPTIDLIPQYLRFLQEWVEHFCKQRQEKVKDVITNCKSCKES------GDTCNSDCEKKCKNKCEAYKKFIEERR--TAAQ-----G-- 837
T2C6.pro                     ----------------GSGS----S-------------CDDIPTIDLIPQYLRFLQEWVEHFCKQRQAKVKDVITNCNSCKES------GNKCKTECKNKCKDECEKYKKFIEACG---TAV----GGTG 830
V1_S.pro                     ----------------CSGD----S-------------SNDMPTIDLIPQYLRFLQEWVEHFCEQRQAKVKDVITNCKSCKES------GNKCKTECKTKCKDECEKYKTFIEDCN---GGG----TG-- 819
WR80.pro                     ----------------GSSD----SGSTTCSGDNGSISCDDIPTIDFIPQYLRFLQEWVEHFCKQRQAKVNAVITNCKSCKES------GDTCNSDCEKKCKIECEKYKTFIEECG--TAAG-----G-- 835
PFL0030cexon1+2.pro          ----------------GSGS----S-------------CDDIPTIDLIPQYLRFLQEWVEHFCKQRQEKVKPVIENCKSCKES------GGTCNGECKTECKNKCEVYKKFIEDCK---GGD----G--- 821
A4PFL0030c exon1+2.pro       ----------------GSGS----S-------------CDDIPTIDLIPQYLRFLQEWVENFCEQRQAKVKDVITNCKSCKES------GNKCKTECKTKCKDECEKYKKFIEAC--GTAGGG---IG-- 826
Newdd2var2CSAsequence.pro    ----------------CSGD----S-------------SNDMPTIDLIPQYLRFLQEWVEHFCKQRQEKVNAVIENCNSCKES------GGTCNSDCKTECKNKCEAYKEFIEDCK---GGG----TG-- 815
McPFL0030c.pro               ----------------GSGS----S-------------CDDIPTIDLIPQYLRFLQEWVEHFCKQRQAKVKDVIENCKSCKES----------GNKCKTECKNKCEAYKKFIENCK---GG-----DG-- 828
HB3-1var2CSA.pro             ----------------GSGS----S-------------CDDMPTIDLIPQYLRFLQEWVEHFCKQRQEKVKDVITNCNSCKECG--DTCNGECKTECKTKCKGECEKYKNFIEEC-NGTADG-----G-- 829
HB3-2var2CSA.pro             ----------------GSGS----S-------------CDDIPTIDLIPQYLRFLQEWVEHFCKQRQEKVNAVIENCNSCKECG--DTCNGECKTECEKKCKIECEKYKTFIEEC--VTAVG-----G-- 828
```

```
                              TAGSSWSKRWDQIYKRYSKYIEDAKRNRKAGTKNC--------GPSSTTNAAXSTT--------ENKCVQSDIDSFFKHLIDIGLTTPSSYLSIVLDDNICGEDKAPWTTYTTYTT--------------
                                       920       930       940       950       960       970       980       990      1000      1010      1020      1030      1040

ALB01044.1                    TAGSPWSKRWDQIYKRYSKHIEDAKRNRKAGTKNC--------GPSSTTNAAEN------------KCVQSDIDSFFKHLIDIGLTTPSSYLSIVLDDNICGADKAPWTTYTTYTTT------------- 920
ALB01053.1                    TAGSPWSKRWDQIYKRYSKHIEDAKRNRKAGTKNC--------GPSSTTNAAEN------------KCVQSDIDSFFKHLIDIGLTTPSSYLSIVLDDNICGADKAPWTTYTTYTTT------------- 920
ETW56275.1                    TAGSSWVKRWDQIYMRYSKYIEDAKRNRKAGTKNC--------GPSSITNVSASTD--------ENKCVQSDIDSFFKHLIDIGLTTPSSYLSIVLDDNICGDDKAPWTTYTTYTTYTTYTTYT------ 936
ALB01048.1                    TAGSPWSKRWDQIYKMYSKHIEDAKRNRKAGTKNC--------GITTGTISGESSGATSGVTTTESKCVQSDIDSFFKHLIDIGLTTPSSYLSIVLDENNCGEDNAPWTTYTTYTTK------------- 940
ALB01041.1                    TSGSSWSKRWDQIYKRYSKYIEDAKRNRKAGTKNCGITTGTISGESSGATSGVTTT--------ENKCVQSDIDSFFKHLIDIGLTTPSSYLSIVLDDNICGEDKAPWTTYTTYTT-------------- 949
ALB01039.1                    TSGSSWSKRWDQIYKRYSKYIEDAKRNRKAGTKNCGITTGTISGESSGATSGVTTT--------ENKCVQSDIDSFFKHLIDIGLTTPSSYLSIVLDDNICGEDKAPWTTYTTYTT-------------- 949
ALB01056.1                    TSGSPWSKRWYQIYMRYSKYIEDAKRNRKAGTKNC--------GTSSGATSGVTTT--------ENKCVQSDIDSFFKHLIDIGLTTPSSYLSIVLDDNICGADKAPWTTYTTYTT-------------- 943
KNC35427.1                    TAGSSWVKRWDQIYKRYSKHIEDAKRNRKAGTKNC--------GPSSITNAAASTD--------ENKCVQSDVDSFFKHLIDIGLTTPSSYLSIVLDENSCGDDKAPWTTYTTYT--------------- 943
ALB01046.1                    TAGSSWSKRWDQIYMRYSKYIEDAKRNRKAGTKSCGITTGTISGESSGANSGVTTT--------ESKCVQSDIDSFFKHLIDIGLTTPSSYLSNVLDENNCGEDKAPWTTYTTYTTT------------- 959
ALB01037.1                    TAGSSWSKRWDQIYKMYSKHIEDAKRNRKAGTKNCGITTGTISGESSGANSGVTTT--------ENKCVQSDIDSFFKHLIDIGLTTPSSYLSIVLDENNCGEDKAPWTTYTTYT--------------- 935
ALB01055.1                    TAGSSWVKRWYQIYMRYSKYIEDAKRNRKAGTKSC--------GTSSGANSGVTTT--------ESKCVQSDIDSFFKHLIDIGLTTPSSYLSIVLDENICGDDKAPWTTYTTYTT-------------- 941
AAQ73924.1                    TAGSPWSKRWDQIYKRYSKHIEDAKRNRKAGTKNC--------GTSSTTNAAASTD--------ENKCVQSDVDSFFKHLIDIGLTTPSSYLSNVLDDNICGADKAPWTTYTTYT--------------- 914
ALB01042.1                    TAGSSWVKRWYQIYMRYSKYIEDAKRNRKAGTKSC--------GTSSGANSGVTTT--------ENKCVQSDIDSFFKHLIDIGLTTPSSYLSIVLDENICGADKAPWTTYTTYTTYTTYTT-------- 946
AJD77410.1                    TAGSSWVKRWDQIYKRYSKHIEDAKRNRKAGTKNC--------GPSSTTNAAES------------KCVQSDIDSFFKHLIDIGLTTPSSYLSIVLDENNCGEDNAPWTTYTTYT--------------- 925
EWC75615.1                    TSGSSWSKRWDQIYMRYSKYIEDAKRNRKAGTKNC--------GTSSTTNAAEN------------KCVQSDIDSFFKHLIDIGLTTPSSYLSIVLDENICGEDKAPWTTYTTYT--------------- 942
ALB01054.1                    TSGSSWSKRWDQIYMRYSKYIEDAKRNRKAGTKNC--------GPSSGANSGVTTT--------ENKCVQSDIDSFFKHLIDIGLTTPSSYLSIVLDENICGADNAPWTTYTTYT--------------- 923
ALB01057.1                    TAESSWSKRWDQIYKRYSKYIEDAKRNRKAGTKNCGITTGTISGESSGANSGVTTT--------ENKCVQSDIDSFFKHLIDIGLTTPSSYLSIVLDDNICGEDNAPWTTYTTYT--------------- 941
ALB01040.1                    TAGSSWSKRWDQIYKMYSKHIEDAKRNRKAGTKNC--------GPSSTTNVSVSTD--------ENKCVQSDIDSFFKHLIDIGLTTPSSYLSIVLDENICGEDKAPWTTYTTYT--------------- 943
ETW41644.1                    TAGSPWSKRWDQIYKRYSKYIEDAKRNRKAGTKNC--------GTSSTTNAAASTA--------ESKCVQSDIDSFFKHLIDIGLTTPSSYLSIVLDENNCGEDKAPWTTYTTYTTYTTYT--------- 930
ALB01043.1                    TAGSSWSKRWDQIYKMYSKHIEDAKRNRKAGTKNC--------GPSSTTNVSVSTD--------ENKCVQSDIDSFFKHLIDIGLTTPSSYLSIVLDENICGEDKAPWTTYTTYT--------------- 943
EUT82883.1                    TSGSPWSKRWDQIYKMYSKHIEDAKRNRKAGTKNC--------GTSSTTST----A--------ESKCVQSDIDSFFKHLIDIGLTTPSSYLSIVLDENNCGDDKAPWTTYTTYTT-------------- 918
ALB01038.1                    TAGSSWVKRWDQIYKRYSKHIEDAKRNRKAGTKNCGTSSTTN--------AAASTA--------ENKCVQSDIDSFFKHLIDIGLTTPSSYLSIVLDDNNCGEDKAPWTTYTTYT--------------- 901
ALB01058.1                    TAGSSWVKRWDQIYKRYSKHIEDAKRNRKAGTKNCGTSSTTN--------AAASTA--------ENKCVQSDIDSFFKHLIDIGLTTPSSYLSIVLDDNNCGEDKAPWTTYTTYT--------------- 901
ALB01059.1                    TSGSSWSKRWDQIYKRYSKYIEDAKRNRKAGTKNC--------GPSSTTNAAASTT--------ENKCVQSDIDSFFKHLIDIGLTTPSSYLSIVLDDNICGADKAPWTTYTTYTT-------------- 912
KNG76633.1                    TAGSSWSKRWDQIYNMYSKHIEDAKRNRKAGTKNC--------GTSSTTN----AA--------ESKCVQSDIDSFFKHLIDIGLTTPSSYLSIVLDENICGDDKAPWTTYTTYTT-------------- 906
ALB01051.1                    TSGSSWVKRWDQIYKRYSKYIEDAKRNRKAGTKSC--------GTSSTTN----AA--------ENKCVQSDIDSFFKHLIDIGLTTPSSYLSIVLDDNICGADNAPWTTYTTYTT-------------- 922
ALB01049.1                    TSGSPWSKRWDQIYKRYSKDIEDAKRNRKAGTKNC--------GTS--------TA--------ENKCVQSDIDSFFKHLIDIGLTTPSSYLSIVLDENNCGDDKAPWTTYTTYTTT------------- 905
EWC75604.1                    TAGSPWSKRWDQIYKRYSKHIEDAKRNRKAGTKNC--------GPSSTTNAAASTD--------ENKCVQSDIDSFFKHLIDIGLTTPSSYLSIVLDENNCGDDKAPWTTYTTYTTT------------- 915
ALB01045.1                    TAGSSWVKRWDQIYKRYSKYIEDAKRNRKAGTKNC--------GPSSTTN----AA--------ESKCVQSDIDSFFKHLIDIGLTTPSSYLSIVLDENNCGEDNAPWTTYTTYTTT------------- 935
ALB01047.1                    TSGSPWSKRWDQIYKRYSKYIEDAKRNRKAGTKSC--------GTSSTTNAAASTA--------ESKCVQSDIDSFFKHLIDIGLTTPSSYLSIVLDDNICGADNAPWTTYTTYTTT------------- 923
7G8.pro                       TAGSSWVKRWDQIYKRYSKHIEDAKRNRKAGTKSCGTST----------------A--------ENKCVQSDIDSFFKHLIDIGLTTPSSYLSIVLDENNCGEDKAPWTTYT------------------ 922
124_8.pro                     TAGSPWSKRWDQIYMRYSKYIEDAKRNRKAGTKNC--------GTSSTTNA----A--------ENKCVQSDIDSFFKHLIDIGLTTPSSYLSIVLDENICGDDKAPWTTYTTYTT-------------- 928
indo.pro                      TAGSSWSKRWDQIYKRYSKYIEDAKRNRKAGTKNC--------GPSSTTNAAEN------------KCVQSDIDSFFKHLIDIGLTTPSSYLSTVLDDNICGEDNAPWTTYTTYTTT------------- 928
KMWII.pro                     TAGSSWVKRWDQIYKRYSKHIEDAKRNRKAGTKSC--------GPSSITNASVSTD--------ENKCVQSDIDSFFKHLIDIGLTTPSSYLSIVLDENNCGEDNAPWTTYTTYTT-------------- 929
M24.pro                       TAGSSWSKRWDQIYKMYSKHIEDAKRNRKAGTKNCGITTGTISGESSGANSGVTTT--------ENKCVQSDIDSFFKHLIDIGLTTPSSYLSIVLDDNICGDDKAPWTTYTTYTTYT------------ 943
MTS1.pro                      TAGSSWVKRWDQIYKRYSKHIEDAKRNRKAGTKNCGITTGTISGESSGATSGVTTT--------ENKCVQSDIDSFFKHLIDIGLTTPSSYLSNVLDDNICGEDNAPWTTYTTY---------------- 937
P13.pro                       TAESSWVKRWDQIYMRYSKYIEDAKRNRKAGTKSC--------GPSSTTNAAASTA--------ENKCVQSDIDSFFKHLIDIGLTTPSSYLSIVLDDNICGADNAPWTTYTTYTTT------------- 938
T2C6.pro                      TAGSPWSKRWDQIYKRYSKHIEDAKRNRKAGTKNC--------GPSSTTNAAEN------------KCVQSDIDSFFKHLIDIGLTTPSSYLSIVLDDNICGADKAPWTTYTTYTT-------------- 927
V1_S.pro                      TAGSSWVKRWDQIYKRYSKHIEDAKRNRKAGTKNC--------GPSSITNAAASTD--------ENKCVQSDIDSFFKHLIDIGLTTPSSYLSNVLDENSCGDDKAPWTTYTTYT--------------- 918
WR80.pro                      TAGSPWSKRWDQIYKRYSKYIEDAKRNRKAGTKNC--------GPSSTT----STA--------ESKCVQSDIDSFFKHLIDIGLTTPSSYLSIVLDDNICGADKAPWTTYTTYTTT------------- 932
PFL0030cexon1+2.pro           TAGSSWVKRWDQIYKRYSKYIEDAKRNRKAGTKNC--------GPSSTTNAAEN------------KCVQSDIDSFFKHLIDIGLTTPSSYLSIVLDDNICGADKAPWTTYTTYTTT------------- 918
A4PFL0030c exon1+2.pro        TAGSPWSKRWDQIYKRYSKHIEDAKRNRKAGTKNC--------GTSSTTNAAASTD--------ENKCVQSDIDSFFKHLIDIGLTTPSSYLSNVLDDNICGADKAPWTTYTTYTT-------------- 926
Newdd2var2CSAsequence.pro     TAGSPWSKRWDQIYKRYSKHIEDAKRNRKAGTKNC--------GTSSTTNAAASTD--------ENKCVQSDVDSFFKHLIDIGLTTPSSYLSNVLDDNICGADKAPWTTYTTYT--------------- 914
McPFL0030c.pro                TAGSSWVKRWDQIYMRYSKYIEDAKRNRKAGTKNC--------GPSSITNVSASTD--------ENKCVQSDIDSFFKHLIDIGLTTPSSYLSIVLDDNICGDDKAPWTTYTTYTTYTTYTTYTTYTTYT 941
HB3-1var2CSA.pro              TSGSSWSKRWDQIYKRYSKYIEDAKRNRKAGTKNC--------GTSSTTNAAASTT--------ENKCVQSDIDSFFKHLIDIGLTTPSSYLSNVLDDNICGEDKAPWTTYTTYT--------------- 928
HB3-2var2CSA.pro              TSGSPWSKRWDQIYKRYSKYIEDAKRNRKAGTKNCGITTGTISGESSGANSGVTTT--------ENKCVQSDIDSFFKHLIDIGLTTPSSYLSIVLDDNICGADNAPWTTYTTYTTYT------------ 938
```

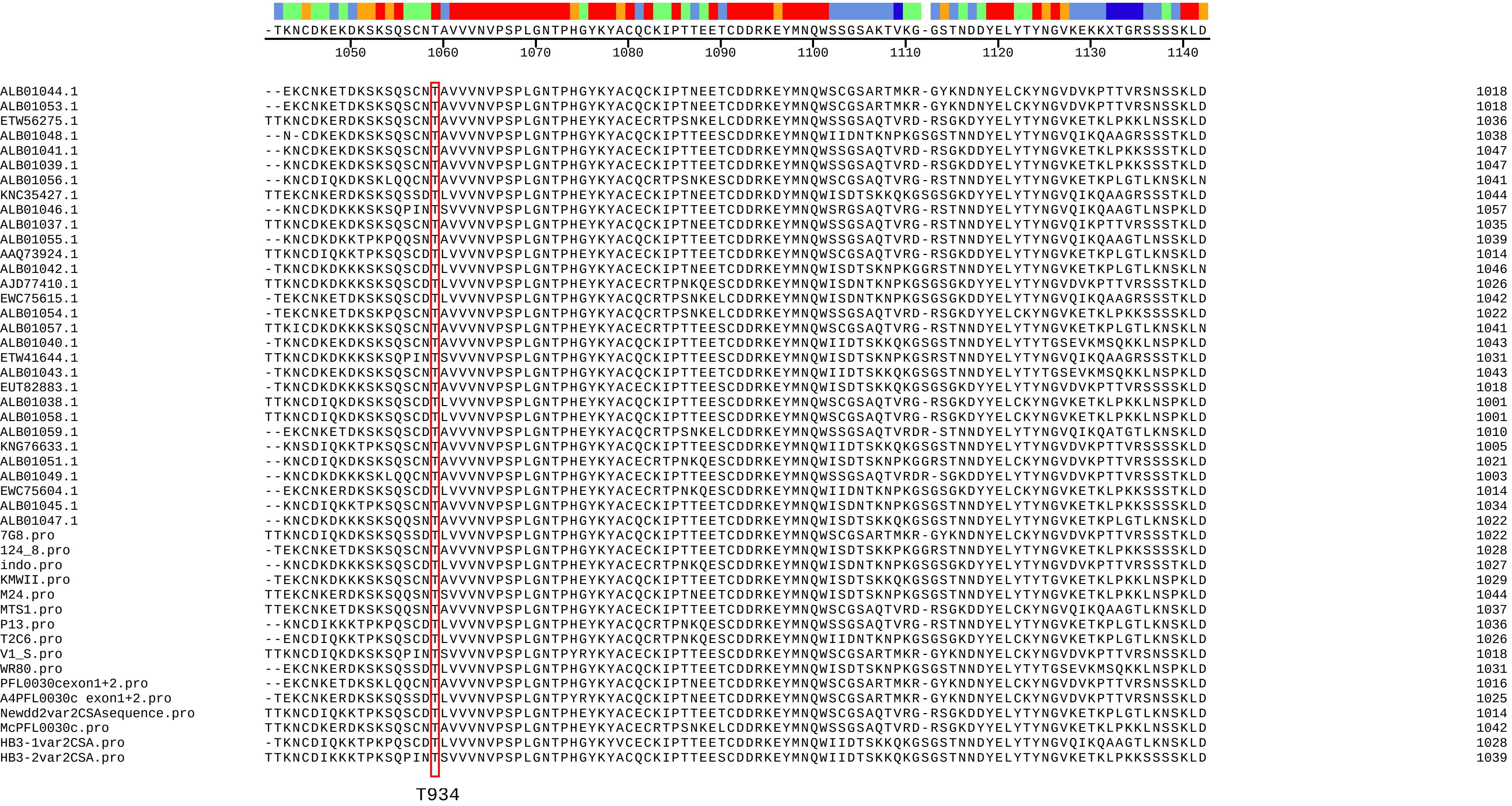

-TKNCDKEKDKSKSQSCNTAVVVNVPSPLGNTPHGYKYACQCKIPTTEETCDDRKEYMNQWSSGSAKTVKG-GSTNDDYELYTYNGVKEKKXTGRSSSSKLD
1050 1060 1070 1080 1090 1100 1110 1120 1130 1140
ALB01044.1 --EKCNKETDKSKSQSCNTAVVVNVPSPLGNTPHGYKYACQCKIPTNEETCDDRKEYMNQWSCGSARTMKR-GYKNDNYELCKYNGVDVKPTTVRSNSSKLD 1018
ALB01053.1 --EKCNKETDKSKSQSCNTAVVVNVPSPLGNTPHGYKYACQCKIPTNEETCDDRKEYMNQWSCGSARTMKR-GYKNDNYELCKYNGVDVKPTTVRSNSSKLD 1018
ETW56275.1 TTKNCDKERDKSKSQSCNTAVVVNVPSPLGNTPHEYKYACECRTPSNKELCDDRKEYMNQWSSGSAQTVRD-RSGKDYYELYTYNGVKETKLPKKLNSSKLD 1036
ALB01048.1 --N-CDKEKDKSKSQSCNTAVVVNVPSPLGNTPHGYKYACQCKIPTTEESCDDRKEYMNQWIIDNTKNPKGSGSTNNDYELYTYNGVQIKQAAGRSSSTKLD 1038
ALB01041.1 --KNCDKEKDKSKSQSCNTAVVVNVPSPLGNTPHGYKYACECKIPTTEETCDDRKEYMNQWSSGSAQTVRD-RSGKDDYELYTYNGVKETKLPKKSSSTKLD 1047
ALB01039.1 --KNCDKEKDKSKSQSCNTAVVVNVPSPLGNTPHGYKYACECKIPTTEETCDDRKEYMNQWSSGSAQTVRD-RSGKDDYELYTYNGVKETKLPKKSSSTKLD 1047
ALB01056.1 --KNCDIQKDKSKLQQCNTAVVVNVPSPLGNTPHGYKYACQCRTPSNKESCDDRKEYMNQWSCGSAQTVRG-RSTNNDYELYTYNGVKETKPLGTLKNSKLN 1041
KNC35427.1 TTEKCNKERDKSKSQSSDTLVVVNVPSPLGNTPHEYKYACECKIPTNEETCDDRKDYMNQWISDTSKKQKGSGSGKDYYELYTYNGVQIKQAAGRSSSTKLD 1044
ALB01046.1 --KNCDKDKKKSKSQPINTSVVVNVPSPLGNTPHGYKYACECKIPTTEETCDDRKEYMNQWSRGSAQTVRG-RSTNNDYELYTYNGVQIKQAAGTLNSPKLD 1057
ALB01037.1 TTKNCDKEKDKSKSQSCNTAVVVNVPSPLGNTPHEYKYACQCKIPTNEETCDDRKEYMNQWSSGSAQTVRG-RSTNNDYELYTYNGVQIKPTTVRSSSTKLD 1035
ALB01055.1 --KNCDKDKKTPKPQQSNTAVVVNVPSPLGNTPHGYKYACQCKIPTTEETCDDRKEYMNQWSSGSAQTVRD-RSTNNDYELYTYNGVQIKQAAGTLNSSKLD 1039
AAQ73924.1 TTKNCDIQKKTPKSQSCDTLVVVNVPSPLGNTPHEYKYACECKIPTTEETCDDRKEYMNQWSCGSAQTVRG-RSGKDDYELYTYNGVKETKPLGTLKNSKLD 1014
ALB01042.1 -TKNCDKDKKKSKSQSCDTLVVVNVPSPLGNTPHGYKYACECKIPTNEETCDDRKEYMNQWISDTSKNPKGGRSTNNDYELYTYNGVKETKPLGTLKNSKLN 1046
AJD77410.1 TTKNCDKDKKKSKSQSCDTLVVVNVPSPLGNTPHEYKYACECRTPNKQESCDDRKEYMNQWISDNTKNPKGSGSGKDYYELYTYNGVDVKPTTVRSSSTKLD 1026
EWC75615.1 -TEKCNKETDKSKSQSCDTLVVVNVPSPLGNTPHGYKYACQCRTPSNKELCDDRKEYMNQWISDNTKNPKGSGSGKDDYELYTYNGVQIKQAAGRSSSTKLD 1042
ALB01054.1 -TEKCNKETDKSKPQSCNTAVVVNVPSPLGNTPHGYKYACQCRTPSNKELCDDRKEYMNQWSSGSAQTVRD-RSGKDYYELCKYNGVKETKLPKKSSSSKLD 1022
ALB01057.1 TTKICDKDKKKSKSQSCNTAVVVNVPSPLGNTPHEYKYACECRTPTTEESCDDRKEYMNQWSCGSAQTVRG-RSTNNDYELYTYNGVKETKPLGTLKNSKLN 1041
ALB01040.1 -TKNCDKEKDKSKSQSCNTAVVVNVPSPLGNTPHGYKYACQCKIPTTEETCDDRKEYMNQWIIDTSKKQKGSGSTNNDYELYTYTGSEVKMSQKKLNSPKLD 1043
ETW41644.1 TTKNCDKDKKKSKSQPINTSVVVNVPSPLGNTPHGYKYACQCKIPTTEESCDDRKEYMNQWISDTSKNPKGSRSTNNDYELYTYNGVQIKQAAGRSSSTKLD 1031
ALB01043.1 -TKNCDKEKDKSKSQSCNTAVVVNVPSPLGNTPHGYKYACQCKIPTTEETCDDRKEYMNQWIIDTSKKQKGSGSTNNDYELYTYTGSEVKMSQKKLNSPKLD 1043
EUT82883.1 -TKNCDKDKKKSKSQSCNTAVVVNVPSPLGNTPHGYKYACECKIPTTEESCDDRKEYMNQWISDTSKKQKGSGSGKDYYELYTYNGVDVKPTTVRSSSSKLD 1018
ALB01038.1 TTKNCDIQKDKSKSQSCDTLVVVNVPSPLGNTPHEYKYACECKIPTTEESCDDRKEYMNQWSCGSAQTVRG-RSGKDYYELCKYNGVKETKLPKKLNSPKLD 1001
ALB01058.1 TTKNCDIQKDKSKSQSCDTLVVVNVPSPLGNTPHEYKYACQCKIPTTEESCDDRKEYMNQWSCGSAQTVRG-RSGKDYYELCKYNGVKETKLPKKLNSPKLD 1001
ALB01059.1 --EKCNKETDKSKSQSCDTAVVVNVPSPLGNTPHEYKYACQCRTPSNKELCDDRKEYMNQWSSGSAQTVRDR-STNNDYELYTYNGVQIKQATGTLKNSKLD 1010
KNG76633.1 --KNSDIQKKTPKSQSCNTAVVVNVPSPLGNTPHGYKYACQCKIPTTEESCDDRKEYMNQWIIDTSKKQKGSGSTNNDYELYTYNGVDVKPTTVRSSSSKLD 1005
ALB01051.1 --KNCDIQKDKSKSQSCNTAVVVNVPSPLGNTPHEYKYACECRTPNKQESCDDRKEYMNQWISDTSKNPKGGRSTNNDYELCKYNGVDVKPTTVRSSSSKLD 1021
ALB01049.1 --KNCDKDKKKSKLQQCNTAVVVNVPSPLGNTPHGYKYACECKIPTTEESCDDRKEYMNQWSSGSAQTVRDR-SGKDDYELYTYNGVDVKPTTVRSSSTKLD 1003
EWC75604.1 --EKCNKERDKSKSQSCDTLVVVNVPSPLGNTPHEYKYACECRTPNKQESCDDRKEYMNQWIIDNTKNPKGSGSGKDYYELCKYNGVKETKLPKKSSSTKLD 1014
ALB01045.1 --KNCDIQKKTPKSQSCNTAVVVNVPSPLGNTPHGYKYACECKIPTTEETCDDRKEYMNQWISDNTKNPKGSGSTNNDYELYTYNGVKETKLPKKSSSSKLD 1034
ALB01047.1 --KNCDKDKKKSKSQQSNTAVVVNVPSPLGNTPHGYKYACQCKIPTTEETCDDRKEYMNQWISDTSKKQKGSGSTNNDYELYTYNGVKETKPLGTLKNSKLD 1022
7G8.pro TTKNCDIQKDKSKSQSSDTLVVVNVPSPLGNTPHGYKYACQCKIPTTEETCDDRKEYMNQWSCGSARTMKR-GYKNDNYELCKYNGVDVKPTTVRSSSTKLD 1022
124_8.pro -TEKCNKETDKSKSQSCNTAVVVNVPSPLGNTPHGYKYACECKIPTTEETCDDRKEYMNQWISDTSKKPKGGRSTNNDYELYTYNGVKETKLPKKSSSSKLD 1028
indo.pro --KNCDKDKKKSKSQSCDTLVVVNVPSPLGNTPHEYKYACECRTPNKQESCDDRKEYMNQWISDNTKNPKGSGSGKDYYELYTYNGVDVKPTTVRSSSTKLD 1027
KMWII.pro -TEKCNKDKKKSKSQSCNTAVVVNVPSPLGNTPHEYKYACQCKIPTTEETCDDRKEYMNQWISDTSKKQKGSGSTNNDYELYTYTGVKETKLPKKLNSPKLD 1029
M24.pro TTEKCNKERDKSKSQQSNTSVVVNVPSPLGNTPHGYKYACQCKIPTNEETCDDRKEYMNQWISDTSKNPKGSGSTNNDYELYTYNGVKETKLPKKLNSPKLD 1044
MTS1.pro TTEKCNKETDKSKSQQSNTAVVVNVPSPLGNTPHGYKYACECKIPTTEETCDDRKEYMNQWSCGSAQTVRD-RSGKDDYELCKYNGVQIKQAAGTLKNSKLD 1037
P13.pro --KNCDIKKKTPKPQSCDTLVVVNVPSPLGNTPHEYKYACQCRTPNKQESCDDRKEYMNQWSSGSAQTVRG-RSTNNDYELYTYNGVKETKPLGTLKNSKLD 1036
T2C6.pro --ENCDIQKKTPKSQSCDTLVVVNVPSPLGNTPHGYKYACQCRTPNKQESCDDRKEYMNQWIIDNTKNPKGSGSGKDYYELCKYNGVKETKPLGTLKNSKLD 1026
V1_S.pro TTKNCDIQKDKSKSQPINTSVVVNVPSPLGNTPYRYKYACECKIPTTEESCDDRKEYMNQWSCGSARTMKR-GYKNDNYELCKYNGVDVKPTTVRSNSSKLD 1018
WR80.pro --EKCNKERDKSKSQSSDTLVVVNVPSPLGNTPHGYKYACQCKIPTTEETCDDRKEYMNQWISDTSKNPKGSGSTNNDYELYTYTGSEVKMSQKKLNSPKLD 1031
PFL0030cexon1+2.pro --EKCNKETDKSKLQQCNTAVVVNVPSPLGNTPHGYKYACQCKIPTNEETCDDRKEYMNQWSCGSARTMKR-GYKNDNYELCKYNGVDVKPTTVRSNSSKLD 1016
A4PFL0030c exon1+2.pro -TEKCNKERDKSKSQSSDTLVVVNVPSPLGNTPYRYKYACQCKIPTNEETCDDRKEYMNQWSCGSARTMKR-GYKNDNYELCKYNGVDVKPTTVRSNSSKLD 1025
Newdd2var2CSAsequence.pro TTKNCDIQKKTPKSQSCDTLVVVNVPSPLGNTPHEYKYACECKIPTTEETCDDRKEYMNQWSCGSAQTVRG-RSGKDDYELYTYNGVKETKPLGTLKNSKLD 1014
McPFL0030c.pro TTKNCDKERDKSKSQSCNTAVVVNVPSPLGNTPHEYKYACECRTPSNKELCDDRKEYMNQWSSGSAQTVRD-RSGKDYYELYTYNGVKETKLPKKLNSSKLD 1042
HB3-1var2CSA.pro -TKNCDIQKKTPKPQSCDTLVVVNVPSPLGNTPHGYKYVCECKIPTTEETCDDRKEYMNQWIIDTSKKQKGSGSTNNDYELYTYNGVQIKQAAGTLKNSKLD 1028
HB3-2var2CSA.pro TTKNCDIKKKTPKSQPINTSVVVNVPSPLGNTPHGYKYACQCKIPTTEESCDDRKEYMNQWIIDTSKKQKGSGSTNNDYELYTYNGVKETKLPKKSSSSKLD 1039
T934
